# Distinct patterns of social contagion under risk and ambiguity

**DOI:** 10.1038/s44271-026-00452-5

**Published:** 2026-04-10

**Authors:** Deng Pan, Qingtian Mi, Jiaxin Zhang, Lusha Zhu, Jian Li

**Affiliations:** 1 School of Psychological and Cognitive Sciences and Beijing Key Laboratory of Behavior and Mental Health, Peking University, Beijing, China; 2 Department of Experimental Psychology, University of Oxford, Oxford, UK; 3 Peking-Tsinghua Center for Life Sciences, Peking University, Beijing, China; 4 IDG/McGovern Institute for Brain Research, Peking University, Beijing, China; 5 Key Laboratory of Machine Perception, Ministry of Education, Peking University, Beijing, China

**Keywords:** Human behaviour, Human behaviour

## Abstract

Our decisions and attitudes are profoundly shaped by social influence. Studies showed that individuals often align their behaviors with peers under uncertainty, a phenomenon known as the “social contagion” effect. Yet it remains unclear how the social contagion effect in behavioral preferences is influenced by disparate others (e.g., conservative vs. adventurous) and modulated by different forms of uncertainty (e.g., risk vs. ambiguity). To address these questions, we conducted two independent experiments (*N*_1_ = 40 and *N*_2_ = 56) to investigate and directly compare the social contagion effect of risk and ambiguity preferences. We found that participants shifted both their risk and ambiguity preferences towards observees after observing their decisions. Intriguingly, participants aligned their risk preferences more toward risk-averse than risk-seeking observees, revealing an asymmetry in risk preference update. In contrast, we did not find reliable evidence for an asymmetric social contagion effect in ambiguity preference. Results from both experiments were fully replicated in a preregistered replication study (*N*_1_ = 61 and *N*_2_ = 62). Together, our findings highlight distinct patterns of social influence on risk and ambiguity preferences, implicating the subtle interplay between social information processing and decision-making under uncertainty.

## Abstract (web summary)

Two experiments with preregistered replications show that observing others shifts both risk and ambiguity preferences. Risk contagion is asymmetric, with stronger alignment toward risk-averse others, whereas ambiguity contagion is more symmetric.

## Introduction

Humans are, by nature, social animals. Our decisions and attitudes are profoundly intertwined with the social influence of individuals around us^1^. Research shows that individuals often adapt their behaviors to align with peers in various scenarios, from financial choices to social decisions^2–7^. This phenomenon, characterized as “social contagion” or “herding”, often manifests in the presence of uncertainty^8^ and spans across different age groups^9,10^. Despite extensive evidence for a robust and widespread effect of social contagion, several open questions remain. First, it is unclear how individuals modify their behavioral preferences in response to peers with opposing preferences in decisions under uncertainty (e.g., conservative vs. adventurous). Second, it also remains to be answered whether the pattern of social contagion effect is universal across different types of uncertainty (e.g., risk vs. ambiguity). Answers to these questions would shed light on the cognitive mechanisms underlying social influence biases and help to elucidate how behavioral polarization may arise from mere social observation.

A growing body of literature in both non-social and social domains suggests that people tend to integrate new information into their beliefs and decisions in a biased manner^11,12^. Specifically, in the non-social domain, prior studies have shown that individuals are biased toward information that either carries positive valence or confirms their prior beliefs, across a wide range of decision-making contexts—from elementary reward learning to high-level belief updating^13–17^. For example, one classic study reported that individuals more readily update their beliefs with “good news” (e.g., lower disease rate for the public) compared to “bad news” (e.g., higher disease rate)^18^. Similarly, in the social domain, although relevant studies are relatively few, existing evidence points to a conservative bias in integrating social information. One recent study, for example, found that in an observational learning task, participants weighed others’ conservative choices more heavily than impulsive ones when making their own decisions^19^. Together, these findings suggest that a biased information integration process may be rooted in human cognition^11,12^. However, it remains unclear whether such biases extend to the social contagion of behavioral preferences toward uncertainty, elicited by mere social observation.

Individuals’ predispositions of uncertainty tolerance greatly shape their decisions under uncertainty. Decades of research have identified two separable preferences toward uncertainty: risk and ambiguity preferences^20^, guiding decisions under different uncertainty contexts. In a risk context, the probabilities of potential outcomes are known (e.g., a coin toss), and decisions are believed to be driven by one’s risk preference^21^. In contrast, in the ambiguity context, the probability distribution of the outcomes is unknown (e.g., guessing the color of the drawn bead from an opaque bag with uncertain number of beads with different colors), and decisions are jointly shaped by both risk and ambiguity preferences^20,22^. Empirical studies showed that an individual’s risk and ambiguity preferences are independent and are likely associated with different psychological and neural substrates^23–26^. However, little is known about the differences between risk and ambiguity preferences in social domains. Specifically, it remains unclear whether social contagion operates consistently across different types of uncertainties or distinctly due to their fundamental difference. This question is particularly relevant in today’s world, where pervasive exposure to social media and the actions of others often lead to mimicking the behavior of social media consumers^27^.

To address the above questions, we performed two independent experiments in the decision contexts of risk and ambiguity, respectively, with separate groups of participants. In each experiment, to measure the social contagion effect induced by social observation, we asked participants to either make their own decisions or observe others’ decisions in matched uncertain contexts. Each participant would observe decisions made by two other individuals with opposing preferences relative to the participant’s own preference (i.e., conservative vs. adventurous). Moreover, previous studies have shown that an individual’s behavioral preferences are influenced by the decision frame (i.e., framing effect), where people prefer safer choices when the options are framed as gains compared to losses^28–30^, in both risk and ambiguity contexts^31,32^. In the current study, we also introduced two different decision frames (i.e., gain and loss) in both experiments to control for the influence of the framing effect on social contagion.

We hypothesize that observing others’ choices would prompt participants to converge toward peer preferences under both risk and ambiguity, eliciting social contagion effects. Based on prior evidence for conservative biases in social information integration, we further hypothesize that social contagion would be asymmetric for risk preferences, favoring alignment with risk-averse over risk-seeking behavior. In contrast, ambiguity contagion would show a comparatively weaker or no systematic asymmetry. Together, these hypotheses allow a direct comparison of how social influence operates across distinct forms of uncertainty.

## Methods

### Participants

#### Experiment 1

Experiment 1 included 40 participants after excluding 5 who showed extreme choice patterns (e.g., never chose gamble options across the whole experiment) or predicted observees’ choices poorly (i.e., the prediction accuracy failed to exceed the 50% chance level after observational learning in at least one session). 19 participants were randomly allocated to the *Gain* decision frame (11 females and 8 males; age:19.10 ± 1.44 y, mean ± SD), whereas the other 21 were in the *Loss* decision frame (9 females and 12 males; 19.05 ± 1.58 y). The sample size was determined a priori using G*Power^33^. Based on a one-sample t-test about the contagion effect in the previous study^5^, we calculated a required minimum of 15 participants per condition, assuming a power of 0.8 and a significant level of *α* = 0.05.

#### Experiment 2

Experiment 2 included 56 participants after excluding 6 from the subsequent analyses because of their extreme choice patterns or ill performance in the prediction phases (similar to Exp. 1). 28 participants were randomly assigned to the *Gain* decision frame (15 females and 13 males; 20.78 ± 2.40 y) and the other 28 participants were in the *Loss* decision frame (17 females and 11 males; 21.43 ± 2.11 y).

#### Participant recruitment

Participants were recruited from the psychological experiments subject pool at Peking University via web advertisements. All participants were right-handed and reported having normal or corrected-to-normal eye vision, no colorblindness, and no history of neurological or psychiatric illnesses. Participant biological sex (female/male) was recorded based on self-report and was balanced across experimental conditions as far as possible during recruitment. Participants’ ages ranged from 18 to 35 years old. Participants’ data on socioeconomic status (SES), communities of descent, or ethnicity were not collected. All studies were approved by the Ethics Committee of the School of Psychological and Cognitive Sciences at Peking University. Informed consent was obtained from all participants before the experiment.

### Experimental task

#### Experiment 1

**Fig. 1:**
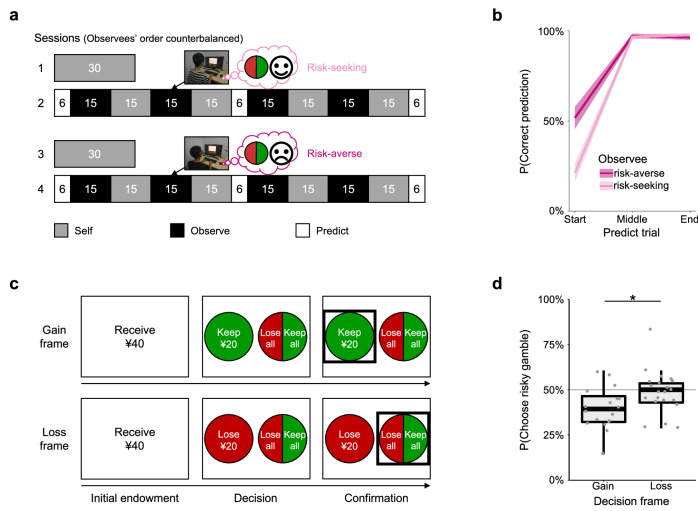
Task design and sanity-check results of Experiment 1. **a** Overall schedule. The color of each mini-block indicates trial types, and the digit on each mini-block denotes the number of trials contained. There are two observees: one in Session 2 is risk-seeking (choosing more gamble options), and another in Session 4 is risk-averse (choosing more sure options) or vice versa. **b** Prediction accuracy in *Predict* trials. The proportion of correct predictions in *Predict* trials increased as the time progressed in both Session 2 and 4 (mean ± SEM across participants) for both the risk-averse and risk-seeking observees (separately plotted). **c** Timeline of an example trial. The player (participants/observees) first received an initial amount of money (duration 2 s) and then made a self-paced decision between a certain and a gamble option. The sure option was to keep/lose part of the endowment with certainty in the Gain/Loss frame, respectively. The gamble option was represented as a pie chart with a probability of keeping (green) or losing (red) all the initial endowment. Once the player made a response, a frame highlighted the chosen option as a confirmation of the decision (duration 1 s). **d** Baseline of participants’ risk attitude in Session 1. Each gray dot indicates one individual’s proportion of choosing the gamble option, and the box plot depicts the distribution among the group. The gray line in the middle indicates a hypothetical risk-neutral agent’s proportion of choosing the gamble. There are 40 participants (*N* = 19 in the Gain frame and *N* = 21 in the Loss frame) in the original Exp. 1. ***p < 0.001; **p < 0.01; *p < 0.05; n.s., not significant.

Our task was adapted from previous studies of the social contagion effect of risk preference and studies of the framing effect^5,30^. It consisted of three different trial types with a fixed schedule (Fig. 1a). In particular, participants were instructed to (1) make decisions for themselves in *Self* trials, (2) observe the decisions made by another person (“observee”) in the previous experiment in *Observe* trials, and (3) predict the observee’s decisions in *Predict* trials. The whole experiment consisted of four sessions. Sessions 2 and 4 involved all three types of trials in a repeated mini-block manner, and Sessions 1 and 3 included only *Self* trials. Here, Sessions 1 and 3 were designed to measure participants’ baseline risk preferences before observation, which would be further compared with their risk preferences in Sessions 2 and 4, respectively, to measure the change in risk preference after observing other’s decisions (i.e., the risk contagion effect). Each block began with a text presentation and a photo indicating the trial type: *Self* blocks: “make your own choice”; *Observe* blocks: “observe their choices” with a photo of the observee; *Predict* blocks: “predict their choices” with a photo of the observee.

Two observees were distinguished by different photos of two males presented in the instruction phase (Fig. 1a). The photos were taken from the back to minimize the potential effects of their appearance. The association between the photo and the observee’s risk preference was randomized across participants. Notably, participants were informed that the observees were as innocent as them and did not have any further knowledge about the task. The observees’ photos were taken in the same experiment room. Besides, the experimenter also took a photo of the participant from the back at the beginning of the experiment and told them that future subjects might observe the record of their choices. By doing so, we tried to convince participants that the choices they observed were made by real people, and this was debriefed after the experiment.

In *Self* trials, participants were first shown an initial amount of money (endowment) they would receive in that trial (e.g., “Receive 40”). Then, a sure and a gamble option were randomly presented on the left or right side of the screen. The sure option indicates that participants could keep (*Gain* frame) or lose (*Loss* frame) a subset of the initial amount (e.g., 20 in Fig. 1c) for sure. In contrast, the gamble option indicates that participants had a certain probability of keeping or losing all the endowment they initially received (please see *Design of experimental stimuli* section for the option design). The probability of keeping and losing all the initial amount in the gamble option was depicted in a pie chart with green and red colors, respectively. Participants had no time pressure to make a choice by pressing one of the two buttons (i.e., the left and right arrow buttons) corresponding to the left and right options with their right hand. Once a decision had been made, the chosen option was highlighted by a frame for 1 s (Confirmation phase; Fig. 1c) and then moved on to the subsequent trial. During the experiment, no feedback concerning trial outcomes was provided to exclude the potential effects of learning from trial feedback. Notably, the only difference between the *Loss* frame and the *Gain* frame was how the sure option was presented; other than that, everything was kept identical.

In *Observe* trials, participants were instructed that other participants’ choices were displayed on the computer screen. Similar to the *Self* trial, the observee’s two options were shown in the decision phase for 2-4 s, and the observee’s actual choice was automatically revealed in the ensuing Confirmation phase. The choice made by the observee was shown on the screen until the participants pressed the space button, providing participants sufficient time for observation. The risk-averse observee chose the gamble options around 25% of the time (simulated by power utility function with *α* = 0.68; see *Data Analyses* for details), and the risk-seeking observee chose the gamble options around 75% of the time (simulated by power utility function with *α* = 1.87).

*Predict* trials were introduced to confirm that the participants learned the observee’s behavioral patterns, and the number of *Predict* trials was smaller than the other two trial types. In *Predict* trials, we instructed participants to predict what the observee would choose as accurately as possible, as their prediction accuracy partially determined their final payoffs. To minimize the differences with *Self* trials, the timeline and the visual display of *Predict* trials were the same as *Self* trials, except that their goals in *Self* and *Predict* trials were different. In addition, no actual choice of the observee was revealed in each trial to prevent further learning from the feedback.

#### The payment of the participants

Participants were paid a show-up fee (20 RMB, ≈ 3 dollars) and an extra fee based on their choices in the experiment. Specifically, at the end of the experiment, we paid participants an additional bonus, including the average amount of money participants obtained from three randomly selected *Self* trials (range 0-50 RMB, ≈ 0-7 dollars) and the reward obtained in one randomly selected *Predict* trial (10 RMB, ≈ 1.5 dollars if the prediction is correct otherwise 0). Therefore, participants were incentivized to learn the observee’s behavioral patterns.

#### Design of the experimental stimuli

In total, we designed 28 different pairs of sure and gamble options in Exp. 1 (Fig. S1, upper panel). The 28 different pairs could be divided into three subsets with the value of sure options (*V*_sure_) as 30, 20, and 10, respectively (corresponding to the left, middle, and right subplots in Fig. S1 upper panel). For each value of the sure options, the combinations of initial endowment and the probability of keeping all the initial endowment in the gamble option were represented as different dots. Take the dot circled in red in the upper panel of Fig. S1 as an example: this dot indicates a trial where the initial endowment is 30 (i.e., value in the *y*-axis), the sure option’s value is 10, and the probability of keeping all the initial endowment in the gamble option is 0.5 (i.e., value in the *x*-axis). Please see also Supplementary Table 1 for all the combinations.

The expected value of the gamble options was distributed on different sides of the indifference curve indicated by the value of the sure option, so that the expected value of the gamble option in some trials is larger than the value of the sure option, while not in some other trials. There are also some trials on the indifference curve. In these trials, the expected value of the gamble option equals the value of the sure option. These trials were also used as the *Predict* trials in Sessions 2 and 4. Notably, there are two gamble options with 0 and 1 probability of keeping all the initial endowment. These two pairs were used as the catch trials to detect whether participants paid attention and chose rationally. The two gamble options with certain probability were repeated once, resulting in a total of 30 trials in Exp. 1 (i.e., the number written on the bar indicating Session 1 in Fig. 1a).

#### Choice patterns of observees

Sessions 2 and 4 showed two different simulated observees for *Observe/Predict* trials. One for Session 2 was risk-averse (choosing gamble options at the odds of only around 25%), and another one for Session 4 was risk-seeking (choosing gamble options at the odds of around 75%) or vice versa (counterbalanced across participants). These parameters were adapted from the previous studies and modified to fit the current study. The choice patterns of the two observees are illustrated in Fig. S1. See also Supplementary Table 1 for the observees’ choices of all the combinations.

#### Experiment 2

The task of Exp. 2 was adapted from Exp. 1 and modified to study the ambiguity contagion effect. Both risky and ambiguous gambles were included, as in the previous studies^23,26^. A risky gamble allows participants to know the probability of either losing or keeping all the initial amount received at the beginning of each trial. In contrast, an ambiguous gamble’s winning probability is made unknown to the participant by covering part of it with a gray mask (Fig. 3c). The covered area indicates the ambiguity level of an ambiguous gamble. Consistent with the classic design, the objective probability in each ambiguous gamble is 50%. The timeline of each trial was the same as Exp. 1, but the overall schedule was modified slightly to allow participants to learn the observees’ risk preference at the beginning of Sessions 2 and 4, before they started to learn the observee’s ambiguity preference. We expected participants to differentiate between risk preference and ambiguity preference through observation.

#### Design of the experimental stimuli

In Exp. 2, we designed 30 unique pairs of sure and gamble options, with half involving ambiguous gambles (Fig. S2, upper panel) and the other half featuring risky gambles (Fig. S2, lower panel). The 30 pairs could be divided into three subsets with the value of sure options (*V*_sure_) as 30, 20, and 10, respectively (corresponding to the left, middle, and right subplots in Fig. S2). Similar to Exp. 1, each value of the sure options was paired with various combinations of reward magnitude (initial endowment) and the ambiguity or risk (probability) of keeping the full initial endowment in the gamble option (Fig. S2). Take the dot circled in red in the upper panel of Fig. S2 as an example: this dot indicates a trial where the initial endowment is 30 (i.e., value in the *y*-axis), the value of the sure option is 10, and the ambiguity level of the gamble option is 0.5 (i.e., value in the *x*-axis). See also Supplementary Table 2 for all the combinations. In some of the 30 pairs, the expected value of the gamble option equals the value of the sure option, if the agent is risk-neutral and ambiguity-neutral (i.e., gray dots on the indifference curve in Fig. S2). We selected 6 out of these pairs for the *Predict* trials (3 in ambiguous contexts and 3 in risky contexts).

#### Choice patterns of observees

In Sessions 2 and 4, two different observees were generated for *Observe/Predict* trials. One was extremely ambiguity-averse but slightly risk-seeking (*α* = 1.1 and *β* = −0.83 in Gilboa & Schmeidler’s Maxmin utility model; see *Data Analyses* for details), while another was extremely ambiguity-seeking but slightly risk-averse (*α* = 0.9 and *β* = 0.83). We compared multiple simulations with various parameter sets and identified the two selected ones that could make observees distinguishable but not too extreme or unreal. The choice patterns of the two observees are illustrated in Fig. S3. The order of these two observees appearing in Sessions 2 and 4 was counterbalanced across participants. Except for the settings mentioned above, other experimental details were kept the same as in Exp. 1. See also Supplementary Table 2 for the observees’ choices of all the combinations.

### Data analyses

Data were analyzed using MATLAB (2017; The Mathworks, Natick, MA) and R (R Development Core Team, 2008). We used *t*-tests, analysis of variance (ANOVA), and regression analyses in the current study, and the data met the assumptions required for these statistical tests.

#### Experiment 1

We used two different approaches to estimate participants’ risk preferences, and they yielded highly consistent results. The model-based approach uses a power utility function. Specifically, each option’s utility is constructed by:

$$U=p \cdot {r}^{\alpha }$$

where *p* denotes the reward probability, and *r* indicates the reward magnitude. *α* is a free parameter that captures the risk preference: *α* equals 1 if risk-neutral and less/greater than 1 if risk-averse/risk-seeking. The utility of each option is then used to determine the probability of making a safe or risky choice through a SoftMax function. Specifically, the participant’s probability of choosing the gamble against the sure option is captured by:

$$q\left({gamble}\right)=\frac{1}{1+{e}^{-\gamma \left(U\left({gamble}\right)-\,U\left({sure}\right)\right)}}$$

Here, γ is a free parameter reflecting the degree of stochasticity in the choice. We estimated the parameter values of α and γ by fitting this model to the participant’s actual choice data using the method of maximum likelihood estimation (see *Model estimation* section).

In a model-free approach, participants’ risk preferences are defined as the proportion of choosing a gamble option relative to the (hypothetical) proportion accepted by a risk-neutral agent^5^: Risk-averse/seeking if relative gamble choices are less/greater than 0. Apart from this difference in the dependent measures, all other procedures are identical across model-free and model-based approaches.

#### Experiment 2

Two different approaches were used to estimate participants’ ambiguity preferences, providing consistent results. First, we employed a model-based approach to estimate participants’ risk and ambiguity preferences simultaneously by implementing Gilboa & Schmeidler’s maxmin utility decision rule^34^. Specifically, the utility function of each gamble option is captured by:

$$U=(p+\beta \times \frac{A}{2})\times {r}^{\alpha }$$

Let *p* be the objective probability (0.5 for ambiguous gambles), *r* be the reward magnitude of the gamble option, and *A* be the ambiguity level (0 for risky gambles). $\alpha$ and *β* are two free parameters, indicating the risk and ambiguity preferences, respectively. People who are ambiguity-averse (*β* < 0) pessimistically believe that the area of gamble under the occluder contains more red than green (thus more likely to lose all the initial amount than keep it), while those who are ambiguity-seeking (*β* > 0) optimistically believe the opposite. The participants’ probability of choosing the gamble against the sure option is determined by the utility of the sure and gamble options, and is modeled by a SoftMax function as in Exp. 1 (explained above).

Second, we used a hybrid model-free approach where participants’ ambiguity preferences are defined as the proportion of choosing a gamble option relative to the (hypothetical) proportion accepted by an ambiguity-neutral (*β*_*hypothetical*_ = 0) agent with the same risk preference as the participant (*α*_*hypothetical*_ = *α*_*self*_). In this approach, positive and negative values indicate ambiguity-seeking and ambiguity-averse preferences, respectively. Apart from this difference in the dependent measures, all other procedures are identical across model-free and model-based approaches.

#### Model estimation

We used the maximum likelihood estimation (MLE) method to estimate each individual’s parameters in each session. The log-likelihood function is written as follows:

$$log\, {{likelihood}} = 	 {\sum}_{t=1}^{T}{Choose}{{{\rm{\_}}}}{gambl}{e}_{t} \cdot \log \left(q{\left({gamble}\right)}_{t}\right) \\ 	 +(1-{Choose}{{{\rm{\_}}}}{gambl}{e}_{t}) \cdot \log \left(1-q{\left({gamble}\right)}_{t}\right)$$

The trial number is indicated by *t*, and *T* represents the total number of trials that differs from sessions. ${Choose\_gambl}{e}_{t}$ = 1 if the participant chose the gamble option on trial *t*; otherwise, ${Choose\_gambl}{e}_{t}\,$= 0. $q{\left({gamble}\right)}_{t}$ is the probability of choosing the gamble option generated from a SoftMax function on trial *t*.

The overall log-likelihood is computed as the sum of the log-likelihoods across all trials. Initial parameter values are randomly sampled as follows: (i) risk preference *α* is drawn from a truncated normal distribution ($\mu$ = 1, *σ* = 0.5, *α* $\ge 0$); (ii) ambiguity preference *β* is drawn from a normal distribution ($\mu$ = 0, *σ* = 0.5); and (iii) inverse temperature *γ* is drawn from a truncated normal distribution ($\mu$ = 0, *σ* = 0.5, *γ* $\ge 0$). We randomly sampled the initial value for each parameter in each estimation and repeated this procedure 1000 times to identify the optimal parameter set with the highest log-likelihood. The randomization of the initial value of parameters could help to avoid the local minimum in model fitting.

### Participants in the replication study

#### Experiment 1

The replication of Exp. 1 included 61 participants after excluding 8 who met our pre-registered exclusion criteria (see “Pre-registered exclusion criteria for replication study”), which is the same as in the original experiment. 31 participants were randomly allocated to the *Gain* decision frame (15 females and 16 males; 22.45 ± 2.46 y), whereas the other 30 were in the *Loss* decision frame (14 females and 16 males; 21.30 ± 2.56 y). We first calculated the effect size of the asymmetric risk contagion effect observed in the original Exp. 1: Cohen’s *d* = 0.9031. Based on this, we then used G*Power to estimate that the minimum number of participants required per condition is 10 in total, assuming a statistical power of 0.8 and *α* = 0.05. Although this analysis suggests a relatively small sample size, we planned to collect a larger sample due to concerns over replication reliability in studies with small sample sizes. Specifically, we aimed to include at least 30 valid participants per condition after applying the exclusion criteria. The sample size for the Replication of Exp. 1 was pre-registered (https://osf.io/f4wuy). The date of preregistration is May 19, 2025.

#### Experiment 2

The replication of Exp. 2 included 62 participants after excluding 7 who met our pre-registered exclusion criteria (see “Pre-registered exclusion criteria for replication study”), which is the same as in the original experiment. 32 participants were randomly allocated to the *Gain* decision frame (15 females and 17 males; 21.84 ± 3.28 y), whereas the other 30 were in the *Loss* decision frame (16 females and 14 males; 22.40 ± 2.22 y). In the original Exp. 2, we found no reliable evidence for the asymmetric ambiguity contagion effect, with participants adjusting their ambiguity preferences similarly toward both ambiguity-seeking and ambiguity-averse observees. To ensure that the absence of a statistically significant asymmetry was not due to insufficient statistical power, we calculated the effect size of the asymmetric ambiguity contagion effect (if it exists) observed in the original Exp. 2 (effect size: Cohen’s *d* = 0.228). A sensitivity analysis (G*Power, two-tailed *t*-test, *α* = 0.05, power = 0.8) revealed that detecting an effect of this small magnitude would require approximately 154 participants per condition—a sample size beyond practical feasibility. To further confirm this absence of asymmetric effect, we plan to recruit at least 30 valid participants per condition in the Replication of Exp. 2, after exclusions. Importantly, this sample size ensures sensitivity to asymmetry comparable in magnitude to the asymmetric risk contagion effect observed in Exp. 1. By recruiting this sample, we aimed to replicate the symmetric ambiguity contagion effect while rigorously testing the robustness of the null asymmetry finding. The sample size for the Replication of Exp. 2 was pre-registered (https://osf.io/f4wuy). The date of preregistration is May 19, 2025.

### Experimental task in the replication study

#### Pre-registered exclusion criteria for replication study

Participants were excluded from the whole data analyses if they met any of the following: (1) low prediction accuracy in *Predict* trials after the observation in at least one session (threshold = 50% chance level), (2) extremely biased or invariant choices, indicating a lack of meaningful engagement (e.g., always choosing gamble or always choosing sure option), (3) explicit disbelief in the experimental setting or suspicion about the manipulation, as revealed during debriefing, and (4) failed to pass the attention check (i.e., catch trials). Moreover, for the calculation of the contagion ratio (defined as the contagion effect divided by the preference distance between the participants and the observee), participants with zero or negative distance toward the observee (same preference as the observee or even more extreme) were excluded from the analyses. All exclusion criteria were pre-registered (https://osf.io/f4wuy).

#### Modifications in the replication study

The design of the replication study remained the same as the original study except for the following three modifications. First, to enhance ecological validity and avoid potential demand effects, we used recorded behavior from two actual human participants instead of using simulated observees as in the original study. Specifically, the observed choices in the replication study were drawn from pre-recorded choices of participants in our earlier experiments, selected from Sessions 1 and 3 in which participants completed the task individually (the “*Self*” trials) and therefore could be used as observees’ choices in the replication study. We selected data from participants whose behavior closely matched the intended observee’s preferences we designed earlier. Second, to protect the observees’ privacy, we replaced the original back-view photographs with cartoon icons representing the observees. These cartoon photos can also mitigate the influence of observees’ gender on the contagion effect. Third, we included a new trial-type judgment task at the end of each mini-block to help rule out the possibility that the observed behavioral changes are due to participants’ confusion between *Self* and *Predict* trials, rather than genuine social contagion effects. Specifically, participants needed to answer a multiple-choice question asking them to indicate which type of block they had just completed—Self, Observe, or Predict—by selecting one of these three options. Participants demonstrated near-perfect accuracy in identifying the task they had just completed (risk contagion experiment: 99.4%; ambiguity contagion experiment: 99.8%), indicating that they could reliably distinguish between task types. All these modifications were pre-registered (https://osf.io/f4wuy).

### Data analyses in the replication study

The same data analysis methods were used in the replication study as in the original study, unless otherwise specified.

### Reporting summary

Further information on research design is available in the Nature Portfolio Reporting Summary linked to this article.

## Results

### Experiment 1: Risk contagion effect

#### Task design and sanity-check results of Experiment 1

The first experiment (Exp. 1) aimed to investigate whether and how an individual’s risk preference was affected by observing others’ decisions (Fig. 1a). To this end, a group of participants (*N* = 40), in alternating trials, were instructed to either make risky choices for themselves or observe the choices of others made in similar contexts where they were both presented with a sure option and a risky gamble. Specifically, each participant completed four task sessions: In Sessions 1 and 3, participants only made their own decisions (*Self* trials); In Sessions 2 and 4, in addition to *Self* trials, participants also predicted (*Predict* trials) another agent’s decisions after observing the agent’s choices (*Observe* trials) in a mini block-wise manner (Fig. 1a). Following previous studies^5^, we generated two distinct sets of agents’ (i.e., observees) choices (i.e., risk-averse and risk-seeking) for participants to observe and predict, but informed participants that real people made these choices. Specifically, one observee was risk-averse, favoring the sure option in general, while the other was risk-seeking, preferring the risky gamble (see “Methods” and Fig. S1 for details). The session order of these two observees (Sessions 2 and 4) was counterbalanced across participants. Specifically, half of the participants observed a risk-seeking observee in Session 2 and a risk-averse observee in Session 4, while the other half observed a risk-averse observee in Session 2 and risk-seeking observee in Session 4. As expected, participants successfully learned the observees’ decision patterns over time (*F*(1,39) = 489.5, *p* < 0.001, ${\eta }_{p}^{2}$ = 0.93, 95% confidence interval (CI) = [0.89, 1.00]; Fig. 1b), as indicated by increased prediction accuracy after observation.

In each trial, irrespective of the trial type, a sure option and a gamble option would always be presented after an initial endowment. By manipulating the way the sure option was presented, we introduced two decision frames: in the *Gain* frame, participants were shown an option to keep part of their initial endowment, while in the *Loss* frame, the option involved losing part of the endowment. The gamble option remained constant across both frames (Fig. 1c; see Methods for details). Participants were randomly assigned to either the *Gain* or the *Loss* frame. We first analyzed participants’ choices in Session 1, where their preferences were unaffected by observations. We found that participants in the *Gain* frame initially chose risky gambles less than those in the *Loss* frame (independent two-sample *t*-test: *t*(37.855) = −2.626, *p* = 0.012, Cohen’s *d* = −0.83, 95% CI = [−1.47, −0.18]; Fig. 1d), consistent with the “framing effect” reported previously^28,30,35,36^.

#### The social contagion effect of risk preference

Next, we investigated whether and how participants’ risk preferences were affected by observing decisions made by the observees. We quantified individuals’ risk preferences in each session using a model-based approach where the risk preference is represented by the parameter *α* in the power utility function: *α* equals 1 indicates risk neutrality, while values greater or less than 1 indicate risk-seeking or risk-averse, respectively (see “Methods” for more details). This model predicted 84.95% of participants’ choices (vs chance: 50%) and there was no statistically significant correlation between the estimated risk preference and the inverse temperature—which captures choice stochasticity, with higher values indicating more deterministic, value-sensitive choices—under the design of the current study (Pearson’s *r* = −0.069, *p* = 0.647, 95% CI = [−0.353, 0.225]; also see Fig. S13). We also used a model-free approach, where risk preference was directly measured as the proportion of gambles chosen by the participant relative to that chosen by a risk-neutral agent, with values higher or lower than zero indicating risk-seeking or risk-averse behavior. As expected, these two approaches generated highly consistent results (*r* = 0.963, *p* < 0.001, 95% CI = [0.950, 0.973]; Fig. S4c). Therefore, we only presented results derived from the model-based approach in the main text (see also Fig. S4 for model-free results).

**Fig. 2:**
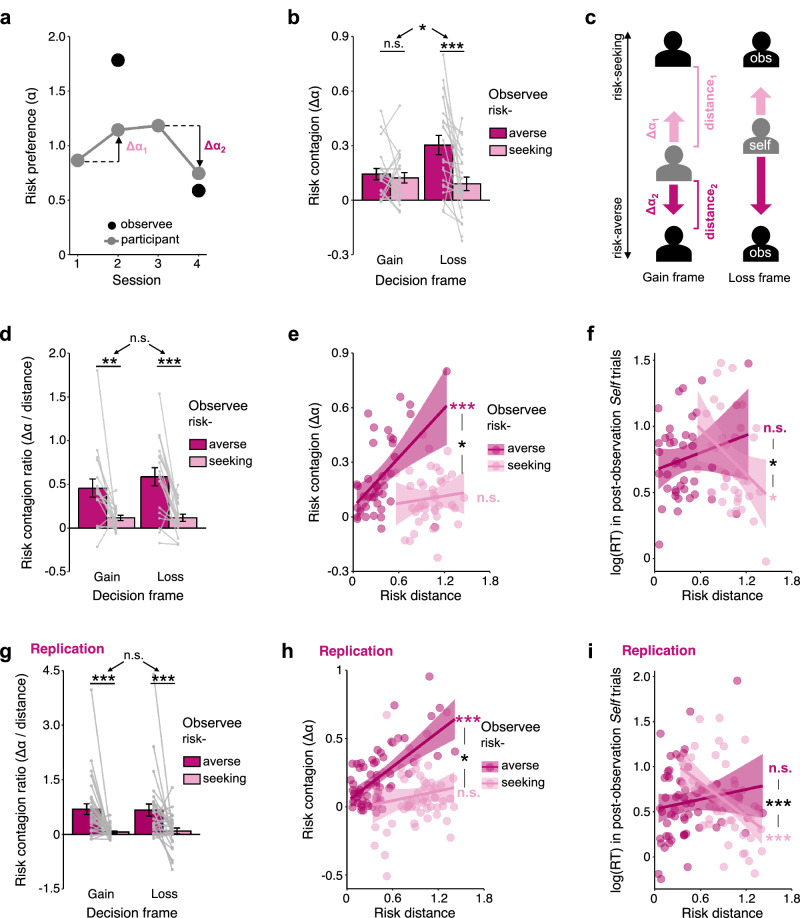
Risk contagion effect. **a** A representative example of the risk contagion effect. The gray line denotes the participant’s risk preference changing across sessions, and the two black dots indicate the two observees’ risk preferences in Sessions 2 and 4, respectively. The risk preference change ($\triangle \alpha$) between pre-/post-observation indicates the degree of risk contagion: positive when the participant conformed to the observee, and negative demonstrated anti-conformity. The participant shown in the example is from the Gain frame. **b** The mean degree of risk contagion as a function of the decision frame and the observee’s risk preference. Gray dots linked by a line represent the risk contagion of each participant when observing risk-averse (magenta) and risk-seeking (pink) observees. Error bars represent the SEM. **c** An illustration of the relationship between risk distance and risk contagion in both Gain and Loss frames. Gray and dark people icons represent the participants and the observees, respectively. The length of thick arrows colored magenta/pink represents the degree of risk-averse/risk-seeking contagion. The risk distance indicates the distance from the participant’s risk preference to the observees’. In the Gain frame, participants are generally closer to risk-averse than risk-seeking observees, yet the risk contagion toward either side is not significantly different. In the Loss frame, participants’ distance toward the risk-averse observee is larger, and the risk-averse contagion also increases proportionally. **d** Risk contagion ratio (defined as the ratio of risk contagion to risk distance) is plotted as a function of the decision frame and observees’ risk preference. Gray dots linked by a line represent the risk contagion ratio of each participant when observing risk-averse (magenta) and risk-seeking (pink) observees. Error bars represent the SEM. **e** Risk contagion as a function of risk distance. **f** The log-transformed reaction time in post-observation self-trials, as a function of risk distance. Fitted regression lines are plotted, and the shaded area around the fitted line reflects 95% confidence intervals (CIs). **g–i** The replication results for (**d**, **e**, **f**), respectively, derived from the replication sample. In (**e**, **f**, **h**, **i**), each magenta/pink point represents a participant when observing risk-averse/risk-seeking observee, respectively. There are 40 participants in the original Exp. 1 and 61 participants in the replication Exp. 1. ***p < 0.001; **p < 0.01; *p < 0.05; n.s., not significant.

By comparing individuals’ risk preferences before and after viewing the observees’ choices, we found that participants shifted their risk preferences toward the observee (i.e., “risk contagion effect”; see Fig. 2a for a representative example). Here, we defined the degree of “risk contagion” ($\Delta \alpha$) as the change in risk preference from pre- to post-observation (i.e., from Session 1 to 2, or from Session 3 to 4), quantifying observees’ influence on participants. Participants were influenced by both risk-seeking and risk-averse observees, with the mean degree of risk contagion significantly above zero (risk-averse: *t*(39) = 6.278, *p* < 0.001, Cohen’s *d* = 0.99, 95% CI = [0.61, 1.37]; risk-seeking: *t*(39) = 4.354, *p* < 0.001, Cohen’s *d* = 0.69, 95% CI = [0.34, 1.03]). Further analysis through mixed ANOVA revealed a significantly stronger contagion effect toward risk-averse observees than risk-seeking ones (*F*(1,38) = 9.843, *p* = 0.003, ${\eta }_{p}^{2}$ = 0.21, 95% CI = [0.05, 1.00]; Fig. 2b). Furthermore, the contagion effect was modulated by the decision frame (interaction: *F*(1,38) = 5.492, *p* = 0.024, ${\eta }_{p}^{2}$ = 0.12, 95% CI = [0.01, 1.00]), though the decision frame alone had no statistically significant influence (*F*(1,38) = 3.330, *p* = 0.076, ${\eta }_{p}^{2}$ = 0.08, 95% CI = [0.00, 1.00]). In the *Gain* frame, there was no statistically significant difference in the risk contagion effect between observees’ risk preference types (post hoc *t*-test: *t*(38) = 0.464, *p* = 0.645, Cohen’s *d* = 0.08, 95% CI = [–0.24, 0.39]), whereas, in the *Loss* frame, the risk contagion effect was more prominent following the observation of a risk-averse compared to a risk-seeking observee (post hoc *t*-test: *t*(38) = 3.888, *p* < 0.001, Cohen’s *d* = 0.63, 95% CI = [0.28, 0.98]; Fig. 2b).

Additional control analyses were performed to exclude other accounts for the observed shifts in risk preference. We tested whether participants simply became more risk-neutral by observing the observees’ choices and found no significant evidence indicating the shift towards risk neutrality across sessions (Fig. S10a). The degree of contagion was not significantly correlated with the proportion of correct predictions in *Predict* trials (Fig. S11a), implying that the contagion effect was not primarily triggered by predicting the observees’ choices.

#### Asymmetry in risk contagion

The above results revealed that participants exhibited stronger risk contagion when observing a risk-averse agent than when observing a risk-seeking agent, only in the *Loss* frame. One possible explanation for this effect could be the distance between the observees’ risk preferences and the participants’ initial risk preferences (“risk distance” for short hereafter, Fig. 2c), with the latter being impacted by the decision frame. To measure and control for the potential influence of the risk distance on the observed risk contagion, we calculated the “risk contagion ratio”, as the risk contagion divided by risk distance, to reflect the change in risk preference relative to the gap left for change. A larger risk contagion ratio may indicate a stronger influence of observation on the participant’s risk preference. To ensure that the risk contagion ratio followed the normal distribution, extreme values falling outside of three standard deviations were excluded, leaving 37 participants in total. An ANOVA analysis revealed a consistent asymmetric risk contagion effect in both the gain and loss frames: subjects were more aligned with risk-averse observees than with risk-seeking ones (*F*(1,35) = 29.980, *p* < 0.001, ${\eta }_{p}^{2}$ = 0.46, 95% CI = [0.26, 1.00]; Fig. 2d). Neither the main effect of the decision frame (*F*(1,35) = 0.651, *p* = 0.425, ${\eta }_{p}^{2}$ < 0.01, 95% CI = [0.00, 1.00]) nor the frame $\times$ preference interaction effect (*F*(1,35) = 0.767, *p* = 0.387, ${\eta }_{p}^{2}$ < 0.01, 95% CI = [0.00, 1.00]) was statistically significant. These results were also replicated with the model-free approach (Fig. S4e) and were robust in both genders (Fig. S14c).

Moreover, we performed linear regression analyses to directly investigate the relationship between risk contagion and risk distance. This regression modeled risk contagion as a function of risk distance separately for the risk-seeking and risk-averse observee while pooling data from both decision frames. In line with the contagion ratio analysis, the regression slope for the risk-averse observee was also significantly steeper than that for the risk-seeking observee (regression coefficients: *coef*_risk-averse_ = 0.453 ± 0.106, 95% CI = [0.245, 0.661], *p* < 0.001; *coef*_risk-seeking_ = 0.068 ± 0.135, 95% CI = [−0.197, 0.333], *p* = 0.613; *coef*_risk-averse_ – *coef*_risk-seeking_ = 0.384 ± 0.171, 95% CI = [0.049, 0.719], *p* = 0.028; Fig. 2e). Similar results were also observed separately for the Gain and the Loss frames (Fig. S12a). Notably, although the regression analysis—which captures group-level tendencies—differs conceptually from the individual-level contagion ratio analysis and could, in principle, yield different results (Fig. S5), both converged on the same conclusion: people have a tendency to learn to become more risk-averse rather than risk-seeking, pointing out the unique attraction of conservative attitudes toward risk.

To further explore the cognitive mechanisms behind risk contagion asymmetry, we also analyzed reaction time (RT) data of post-observation trials, focusing on how participants’ responses were influenced by their preference distance to the observee. We found that participants who observed a risk-seeking observee responded faster as the observer-observee risk preference distance increased (*coef*_risk-seeking_ = −0.575 ± 0.266, 95% CI = [−1.096, −0.054], *p* = 0.037); however, no statistically significant effect was found after the risk-averse observations (*coef*_risk-averse_ = 0.221 ± 0.191, 95% CI = [−0.153, 0.595], *p* = 0.254). Therefore, both the risk contagion behaviors and RTs (*coef*_risk-averse_ – *coef*_risk-seeking_ = 0.795 ± 0.324, 95% CI = [0.160, 1.430], *p* = 0.016; Fig. 2f) showed significant asymmetric contagion effects on the relative risk preference of the observees.

#### Replication of asymmetry in risk contagion

We replicated the asymmetric risk contagion effect with another independent cohort of participants in a preregistered study (*N* = 61, see “Methods” for details). ANOVA analysis on contagion ratio with the replication sample again revealed a consistent asymmetric risk contagion effect in both the gain and loss frames: subjects were more aligned with risk-averse than with risk-seeking observees (*F*(1,59) = 26.408, *p* < 0.001, ${\eta }_{p}^{2}\,$= 0.31, 95% CI = [0.16, 1.00]; Fig. 2g). Neither the main effect of the decision frame (*F*(1,59) = 0.000, *p* = 0.983, ${\eta }_{p}^{2}$ < 0.01, 95% CI = [0.00, 1.00]) nor the frame $\times$ preference interaction effect (*F*(1,59) = 0.048, *p* = 0.828, ${\eta }_{p}^{2}$ < 0.01, 95% CI = [0.00, 1.00]) was statistically significant. In line with the contagion ratio analysis, linear regression analyses showed the regression slope for the risk-averse observee was also significantly steeper than that for the risk-seeking observee (regression coefficients: *coef*_risk-averse_ = 0.426 ± 0.070, 95% CI = [0.289, 0.563], *p* < 0.001; *coef*_risk-seeking_ = 0.118 ± 0.105, 95% CI = [−0.088, 0.324], *p* = 0.226; *coef*_risk-averse_ – *coef*_risk-seeking_ = 0.308 ± 0.124, 95% CI = [0.065, 0.551], *p* = 0.015; Fig. 2h). We also replicated the asymmetric effects found in RT results. Participants who observed a risk-seeking observee responded faster as the observer-observee risk preference distance increased, but not after the risk-averse observations (*coef*_risk-seeking_ = -0.628 ± 0.161, 95% CI = [−0.943, −0.313], *p* < 0.001; *coef*_risk-averse_ = 0.183 ± 0.169, 95% CI = [−0.148, 0.514], *p* = 0.283; *coef*_risk-averse_ – *coef*_risk-seeking_ = 0.813 ± 0.237, 95% CI = [0.348, 1.278], *p* < 0.001; Fig. 2i). These replication results confirmed the robustness of the asymmetric risk contagion effect observed in the original experiment.

### Experiment 2: Ambiguity contagion effect

#### Task design of Experiment 2

Next, we investigated whether and how an individual’s ambiguity preference was affected after observing others’ decisions in an ambiguous decision context (i.e., the ambiguity contagion effect). Previous studies have suggested that people’s attitudes toward ambiguity and risk are separable at both the behavioral and neural levels^23,26^. We thus expected that the ambiguity contagion effect might reveal a pattern different from the risk contagion effect.

**Fig. 3:**
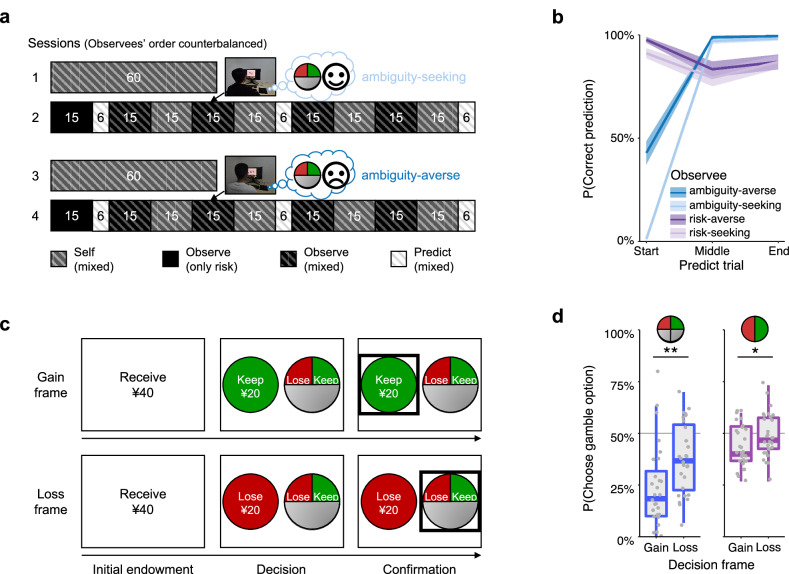
Task design and behavioral results of Experiment 2. **a** Overall schedule of Exp. 2. The schedule closely follows that of Exp. 1 (Fig. 1a), with two notable adjustments: (i) Sessions 1 and 3 were extended to 60 trials to include both risk and ambiguity decision trials, and (ii) at the start of Sessions 2 and 4, we introduced 15 Observe trials focusing exclusively on risk decisions. **b** Results in the Predict trials. The proportion of correct predictions in Predict trials is plotted as a function of time (mean ± SEM across participants): plotted separately for ambiguity-averse (also slightly risk-seeking) and ambiguity-seeking observees (also slightly risk-averse). Data from Sessions 2 and 4 are collapsed. **c** Timeline of an example trial in decision contexts under ambiguity. In the example, part of the gamble option is covered by a gray mask to create ambiguity. The occluder area denotes the ambiguity level (50% in this case). Notably, the gamble options include both ambiguous and risky gambles (no occluder, ambiguity level = 0%). **d** Baseline probability of choosing ambiguous (left) and risky (right) gambles in Session 1. Each gray dot indicates one individual, and the box plot depicts the distribution among the respective groups. There are 56 participants (*N* = 28 in the Gain frame and *N* = 28 in the Loss frame) in the original Exp. 2. ***p < 0.001; **p < 0.01; *p < 0.05; n.s., not significant.

To test the above hypothesis, in Exp. 2, we recruited a new group of participants (*N* = 56) to play a gambling task similar to Exp. 1 but in an ambiguous choice context (Fig. 3a). Participants also had to choose between a sure option and an uncertain gamble. However, the gamble options in Exp. 2 were either risky or ambiguous. While a risky gamble revealed the probability of the outcome, an ambiguous gamble’s exact probability was covered with a gray mask (Fig. 3c), the area of which indicated the ambiguity level. Unbeknownst to the participants, the underlying probability in each ambiguous gamble was fixed at 50% (see “Methods” for more details).

Again, two types of observees were generated (ambiguity-averse and ambiguity-seeking, see details in “Methods” and Fig. S3). Since both risk and ambiguity preferences drive the observees’ choices, it was essential to ensure that participants could clearly distinguish between them. To this end, the ambiguity-averse observee was designed to be slightly risk-seeking while the ambiguity-seeking observee was slightly risk-averse. Besides, participants were exposed to the observees’ choices with only risky gambles at the beginning of Sessions 2 and 4. This exposure could familiarize participants with the observees’ risk preferences, enabling more precise differentiation between risk and ambiguity preferences in later *Observe* trials. As a result, in the first *Predict* block, they successfully learned the observees’ risk decisions (observed) but failed to predict their ambiguity decisions (not observed). As the session progressed, participants quickly and accurately learned the observees’ ambiguity preferences and kept their prediction accuracy close to 100% (Fig. 3b).

In line with Exp. 1, separate groups of subjects played in the *Gain* and *Loss* frames. We found that in Session 1 (before observation), people favored sure options over ambiguous gambles in both decision frames, and more notably so in the *Gain* frame than in the *Loss* frame (independent two-sample *t*-test: *t*(53.101) = –2.818, *p* = 0.007, Cohen’s *d* = –0.75, 95% CI = [−1.29, −0.21]; Fig. 3d left), consistent with previous studies^32^. They also responded more quickly in the *Gain* frame than in the *Loss* frame (*t*(51.104) = −2.380, *p* = 0.021, Cohen’s *d* = −0.67, 95% CI = [−1.28, −0.10]). Besides, for risk decisions, people opted for sure options over risky gambles more frequently in the *Gain* frame than in the *Loss* frame (*t*(53.522) = −2.050, *p* = 0.045, Cohen’s *d* = −0.55, 95% CI = [−1.08, −0.01]; Fig. 3d right), consistent with results from Exp. 1.

#### The social contagion effect of ambiguity preference

Since people’s decisions in the ambiguous context relied jointly on their preferences toward risk and ambiguity, the proportion of choosing ambiguous gambles does not directly reflect their ambiguity preference. Therefore, we simultaneously estimated each individual’s risk and ambiguity preference by using a power utility function that considers the effect of ambiguity on the perceived winning probability, based on Gilboa & Schmeidler’s maxmin expected utility model. This method has been successfully used to separate an individual’s risk and ambiguity preferences in previous studies^26,34^. In particular, the utility of each option is assessed by:

$$U = \left(p+\beta \times \frac{A}{2}\right)\times {r}^{\alpha }$$

where *p* is the objective probability (0.5 for ambiguous gambles), *r* is the reward magnitude, and *A* represents the ambiguity level (0 for risky gambles). Here, *α* and *β* are free parameters reflecting risk and ambiguity preferences, respectively. Ambiguity-seeking individuals (*β* > 0) optimistically believe that the area under the mask of an ambiguous gamble contains more winning color, while ambiguity-averse individuals (*β* < 0) pessimistically believe the opposite. As in previous studies^23,26^, there was no statistically significant correlation between the estimated parameters *α* and *β* in the first session (*r* = −0.22, *p* = 0.089, 95% CI = [−0.44, 0.03]; Fig. S7a; see also Fig. S13 for parameter recovery analysis). Under the current design, 85.71% of participants’ choices in the ambiguous decision contexts across all sessions could be accurately predicted by the maxmin expected utility model.

**Fig. 4:**
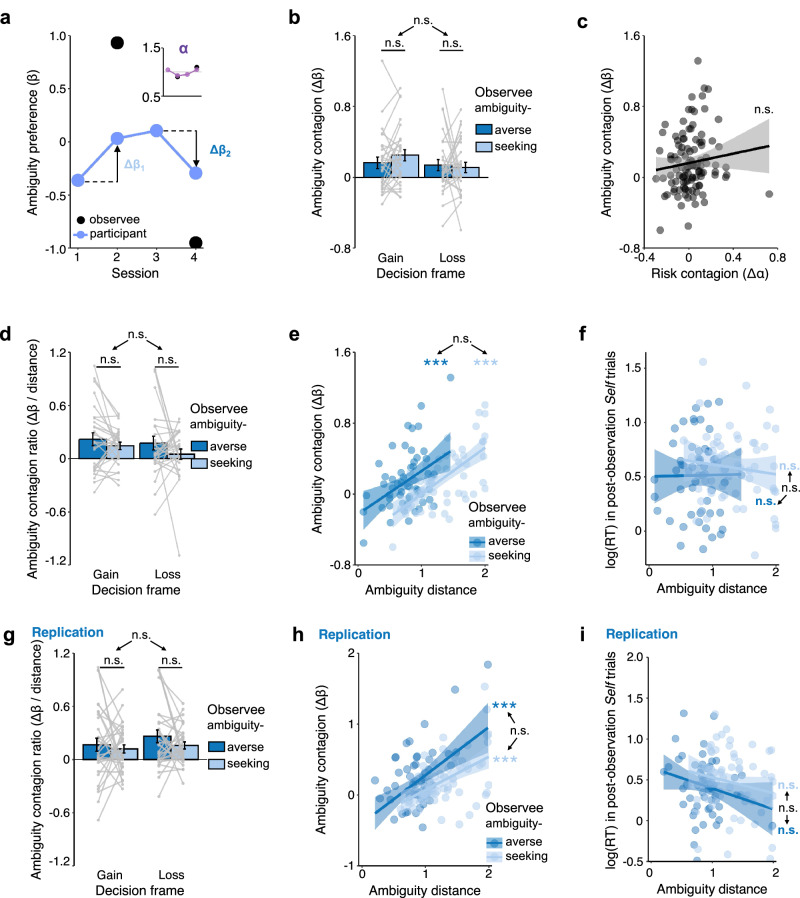
Ambiguity contagion effect. **a** A representative example of the ambiguity contagion effect. The light blue line denotes the participant’s ambiguity preference changing across sessions, and the two dark dots indicate the observees’ ambiguity preference in Session 2 and 4, respectively. The ambiguity preference change ($\Delta \beta$) between pre- and post-observation indicates the degree of ambiguity contagion. The inset shows the same participant’s risk preference changing across sessions. **b** Degree of ambiguity contagion as a function of decision frame and observees’ ambiguity-preference type (mean ± SEM). Gray dots linked by a line represent the ambiguity contagion of each participant when observing ambiguity-averse and ambiguity-seeking observees. **c** The relationship between ambiguity contagion and risk contagion. Each gray dot represents one type of contagion (observing the ambiguity-seeking or averse observee) of each participant. **d** Degree of ambiguity contagion ratio as a function of the decision frame and the observee’s ambiguity-preference type (mean ± SEM). Gray dots linked by a line represent the ambiguity contagion ratio of each participant when observing ambiguity-averse and ambiguity-seeking observees. **e** Ambiguity contagion as a function of ambiguity distance. **f** The log-transformed reaction time in post-observation Self trials, as a function of ambiguity distance. Fitted regression lines are plotted, and the shaded area around the fitted lines reflects 95% CIs. **g**–**i** The replication results for panels (**d**, **e**, **f**), respectively, derived from the replication sample. In (**e**, **f**, **h**, **i**), each dark-blue/light-blue point represents a participant when observing the ambiguity-averse/ambiguity-seeking observee, respectively. There are 56 participants in the original Exp. 2 and 62 participants in the replication Exp. 2. ***p < 0.001; **p < 0.01; *p < 0.05; n.s., not significant.

Upon comparing individuals’ ambiguity preferences before and after observing others, we found that participants shifted their ambiguity preferences towards the observees, yielding an ambiguity contagion effect (see Fig. 4a for a representative example). Again, the degree of ambiguity contagion ($\Delta \beta$) was defined as the change in ambiguity preference from pre- to post-observation session. The mean degree of ambiguity contagion was significantly positive in both decision frames (Gain: *t*(27) = 4.104, *p* < 0.001, Cohen’s *d* = 0.78, 95% CI = [0.35, 1.19]; Loss: *t*(27) = 2.896, *p* = 0.007, Cohen’s *d* = 0.55, 95% CI = [0.15, 0.94]), with no statistically significant differences between each other (independent two-sample *t*-test: *t*(51.004) = −1.451, *p* = 0.159, Cohen’s *d* = –0.39, 95% CI = [−0.92, 0.14]). Despite not being the focus of Exp. 2, we still observed participants’ risk preferences exhibited small post-observation changes (see Fig. 4a, inset), measured as total risk contagion as in Exp. 1. Importantly, there was no statistically significant correlation between the observed ambiguity contagion effect and the risk contagion effect across participants (*r* = 0.117, *p* = 0.220, 95% CI = [−0.07, 0.30]; Fig. 4c), indicating that the newly identified ambiguity contagion was not a consequence of the risk contagion.

Different from the pattern of risk contagion shown in Exp. 1 (Fig. 2b), there was no statistically significant modulation of degree of ambiguity contagion by either the observee’s ambiguity preference (*F*(1,54) = 0.336, *p* = 0.565, ${\eta }_{p}^{2}$ < 0.01, 95% CI = [0.00, 1.00]), the decision frame (*F*(1,54) = 1.277, *p* = 0.263, ${\eta }_{p}^{2}$ < 0.01, 95% CI = [0.00, 1.00]), or the interaction between the two variables (*F*(1,54) = 1.223, *p* = 0.274, ${\eta }_{p}^{2}$ < 0.01, 95% CI = [0.00, 1.00]; Fig. 4b). Nonetheless, consistent with Exp. 1, a similar pattern with respect to the risk contagion effect was observed in Exp. 2, where the risk contagion effect was and only was significant when observing the risk-averse observee in the *Loss* frame (Fig. S8a). Notably, the total effect size of the risk contagion effect in Exp. 2 was much smaller than that in Exp. 1, because the observees’ risk preferences by design did not deviate much from risk neutral. Besides, despite not being the focus of Exp. 2, we still replicated a similar pattern of asymmetric risk contagion, with a smaller but significant effect (Fig. S8c), where participants showed a stronger contagion ratio when observing risk-averse observees than risk-seeking ones. Although risk preferences changed little here, linear regressions still showed that risk distance exerted a more significant influence on risk-averse than risk-seeking contagion (Fig. S8b), consistent with Exp. 1. Together, these findings showed that participants’ risk and ambiguity preferences change independently, implying that they might be associated with distinct psychological bases. Moreover, such intrinsic differences could be explicitly revealed and amplified by observing others’ decisions in similar decision contexts.

We also replicated the identified ambiguity contagion effect using a hybrid model-free approach. Specifically, we first estimated participants’ risk preferences using their choices on pure risky gambles. Then, we used the estimated risk preference to simulate each individual’s hypothetical choices for ambiguous gambles, assuming they were ambiguity neutral. The hybrid model-free ambiguity preference was defined as the actual proportion of ambiguous gambles accepted relative to their hypothetical ambiguity-neutral choices. As expected, ambiguity preferences from model-based and hybrid model-free approaches were strongly correlated (*r* = 0.691, *p* < 0.001, 95% CI = [0.62, 0.75]; Fig. S7b), with consistent contagion patterns observed across both methods (Fig. S7c).

Control analyses revealed that participants did not simply become more ambiguity-neutral after observation (Fig. S10b). Neither risk nor ambiguity contagion effect was correlated with participants’ prediction performances (Fig. S11b). In sum, results from Exp. 2 suggested that social influences could change an individual’s ambiguity preference, and such contagion effect was consistent across different conditions.

#### No reliable evidence for asymmetry in ambiguity contagion

Again, we reassessed the ambiguity contagion effect while controlling for the distance of ambiguity preferences between participants and observees. Like Exp. 1, we calculated the ambiguity contagion ratio by dividing the change of ambiguity preference ($\Delta \beta$) by ambiguity preference distance. Again, extreme ratio values falling outside of three standard deviations were excluded to ensure a normal distribution, leaving 54 participants in total. However, different from the patterns of risk contagion ratio observed in Exp. 1, an ANOVA analysis on ambiguity contagion ratio showed that there was no statistically significant modulation of ambiguity contagion ratio by either the observee’s ambiguity preference (*F*(1,52) = 2.760, *p* = 0.103, ${\eta }_{p}^{2}$ = 0.05, 95% CI = [0.00, 1.00]), the decision frame (*F*(1,52) = 1.022, *p* = 0.317, ${\eta }_{p}^{2}$ < 0.01, 95% CI = [0.00, 1.00]), or the interaction of these two variables (*F*(1,52) = 0.184, *p* = 0.670, ${\eta }_{p}^{2}$ < 0.01, 95% CI = [0.00, 1.00]; Fig. 4d). These results indicate no reliable evidence for asymmetry in the influence of observees’ ambiguity preferences on participants’ ambiguity contagion (Bayes Factor, BF_10_ = 0.55).

In addition, results from the linear regression analyses, which modeled ambiguity contagion as a function of ambiguity distance across participants, also supported no statistically significant difference in the influence of ambiguity distance on ambiguity contagion when observing ambiguity-averse and ambiguity-seeking agents. Specifically, the ambiguity contagion effect was positively correlated with ambiguity distance, no matter when the observee was ambiguity-averse or ambiguity-seeking (*coef*_ambiguity-averse_ = 0.486 ± 0.135, 95% CI = [0.221, 0.751], *p* < 0.001; *coef*_ambiguity-seeking_ = 0.522 ± 0.082, 95% CI = [0.361, 0.683], *p* < 0.001; Fig. 4e). However, unlike the risky condition, the reaction time in the ambiguity condition did not change significantly with the ambiguity distance after observing either ambiguity-averse or ambiguity-seeking observees (*coef*_ambiguity-averse_ = 0.013 ± 0.168, 95% CI = [−0.316, 0.342], *p* = 0.941; *coef*_ambiguity-seeking_ = –0.090 ± 0.112, 95% CI = [−0.309, 0.129], *p* = 0.423; Fig. 4f).

#### Replication study of ambiguity contagion effect

We replicated the observed ambiguity contagion effect with another independent sample of participants in a preregistered study (*N* = 62, see “Methods”" for details). ANOVA analysis on contagion ratio with the replication sample again revealed no statistically significant asymmetry in ambiguity contagion effect across both the Gain and Loss frames. Specifically, there was no statistically significant modulation of ambiguity contagion ratio by either the observee’s ambiguity preference (*F*(1,60) = 1.699, *p* = 0.197, ${\eta }_{p}^{2}$ = 0.03, 95% CI = [0.00, 1.00]), the decision frame (*F*(1,60) = 1.142, *p* = 0.290, ${\eta }_{p}^{2}$ < 0.01, 95% CI = [0.00, 1.00]), or the interaction of these two variables (*F*(1,60) = 0.244, *p* = 0.623, ${\eta }_{p}^{2}$ < 0.01, 95% CI = [0.00, 1.00]; Fig. 4g). These results again show no reliable evidence for asymmetry in the influence of observees’ ambiguity preferences on participants’ ambiguity contagion (Bayes Factor, BF_10_ = 0.31). In line with the contagion ratio analysis, linear regression analyses showed the ambiguity contagion effect was positively correlated with ambiguity preference distance for both ambiguity-averse and ambiguity-seeking observees yet without asymmetry (*coef*_ambiguity-averse_= 0.678 ± 0.156, 95% CI = [0.372, 0.984], *p* < 0.001; *coef*_ambiguity-seeking_ = 0.469 ± 0.106, 95% CI = [0.261, 0.677], *p* < 0.001; *coef*_ambiguity-averse_ – *coef*_ambiguity-seeking_ = 0.209 ± 0.186, 95% CI = [−0.156, 0.574], *p* = 0.263; Fig. 4h). We also replicated the RT results by showing again that RT did not significantly change with the ambiguity distance when the observee was either ambiguity-averse or ambiguity-seeking (*coef*_ambiguity-averse_ = −0.257 ± 0.140, 95% CI = [−0.531, 0.017], *p* = 0.084; *coef*_ambiguity-seeking_ = –0.186 ± 0.130, 95% CI = [−0.441, 0.069], *p* = 0.141; *coef*_ambiguity-averse_ – *coef*_ambiguity-seeking_ = 0.071 ± 0.196, 95% CI = [−0.313, 0.455], *p* = 0.756; Fig. 4i).

### Comparing contagion effects of risk and ambiguity preferences across experiments

**Fig. 5:**
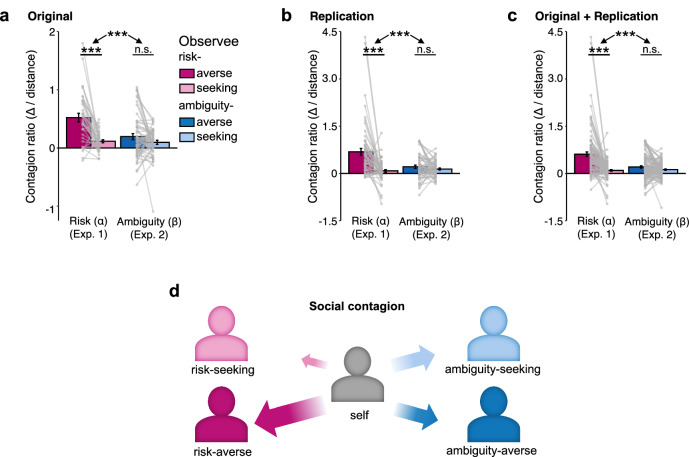
Asymmetric risk and more symmetric ambiguity contagion effects across two experiments. **a** Model-based contagion ratio of risk preference (*α* in Exp. 1) and ambiguity-preference (*β* in Exp. 2) as a function of the observees’ risk/ambiguity preferences (mean ± SEM). Gray dots linked by a line represent the risk/ambiguity contagion of each participant when observing risk-averse/seeking and ambiguity-averse/seeking observees. The uppermost asterisk indicates that the interaction effect between uncertainty type (risk/ambiguity) and observees’ uncertainty preference is significant. **b** The replication results from the replication sample. **c** The results after pooling the data from both original and replication studies. **d** Summary illustration of an asymmetric risk contagion effect (stronger risk-averse contagion than risk-seeking contagion) and a more symmetric ambiguity contagion effect (no significant difference between ambiguity-averse and ambiguity-seeking contagion). There are 96 participants in the original study and 123 participants in the replication study. ***p < 0.001; **p < 0.01; *p < 0.05; n.s. not significant.

In sum, we observed asymmetric social influence on risk contagion and more symmetric social influence on ambiguity contagion across two separate experiments. To draw a more comprehensive conclusion, we used the contagion ratio as a unified measure to compare the social contagion effect across the two experiments. We investigated the impact of uncertainty type (risk/ambiguity; between-group factor) and the observee’s preference type (averse/seeking; within-group factor) on the contagion ratio, pooled across decision frames. An ANOVA analysis showed that the contagion ratio was significantly more prominent in the context of risk than ambiguity (*F*(1,94) = 8.66, *p* = 0.004, ${\eta }_{p}^{2}$ = 0.08, 95% CI = [0.02, 1.00]) and when the observee was uncertainty-averse than uncertainty-seeking (*F*(1,94) = 23.45, *p* < 0.001, ${\eta }_{p}^{2}$ = 0.20, 95% CI = [0.09, 1.00]). The interaction of uncertainty type and the observee’s preference type was significant (*F*(1,94) = 9.51, *p* = 0.002, ${\eta }_{p}^{2}$ = 0.09, 95% CI = [0.02, 1.00]), where the contagion ratio of the risk-averse observee was significantly larger than risk-seeking (post hoc *t*-test: *t*(94) = 5.516, *p* < 0.001, Cohen’s *d* = 0.57, 95% CI = [0.35, 0.79]), but ambiguity-averse and ambiguity-seeking contagion ratio showed no statistically significant difference (post hoc *t*-test: *t*(94) = 1.593, *p* = 0.115, Cohen’s *d* = 0.16, 95% CI = [−0.04, 0.37], Bayes Factor, BF_10_ = 0.55; Fig. 5a).

The same analysis on our replication sample (*N* = 123) generated consistent results of asymmetric risk contagion and more symmetric ambiguity contagion effects (uncertainty context (risk > ambiguity): *F*(1,121) = 9.715, *p* = 0.002, ${\eta }_{p}^{2}$ = 0.07, 95% CI = [0.02, 1.00]; observee type (averse > seeking): *F*(1,121) = 27.12, *p* < 0.001, ${\eta }_{p}^{2}$ = 0.18, 95% CI = [0.09, 1.00]; interaction: *F*(1,121) = 16.80, *p* < 0.001, ${\eta }_{p}^{2}$ = 0.12, 95% CI = [0.05, 1.00]; post hoc *t*-test in risk context (averse > seeking): *t*(121) = 6.577, *p* < 0.001, Cohen’s *d* = 0.60, 95% CI = [0.40, 0.79]; post hoc *t*-test in ambiguity context (averse > seeking): *t*(121) = 0.810, *p* = 0.419, Cohen’s *d* = 0.07, 95% CI = [−0.10, 0.25], Bayes Factor, BF_10_ = 0.31; Fig. 5b). After pooling the data from both the original and replication studies (*N* = 219), the result pattern remained the same (uncertainty context (risk > ambiguity): *F*(1,217) = 18.38, *p* < 0.001, ${\eta }_{p}^{2}$ = 0.08, 95% CI = [0.03, 1.00]; observee type (averse > seeking): *F*(1,217) = 47.41, *p* < 0.001, ${\eta }_{p}^{2}$ = 0.18, 95% CI = [0.11, 1.00]; interaction: *F*(1,217) = 26.43, *p* < 0.001, ${\eta }_{p}^{2}$ = 0.11, 95% CI = [0.05, 1.00]; post hoc *t*-test in risk context (averse > seeking): *t*(217) = 8.464, *p* < 0.001, Cohen’s *d* = 0.57, 95% CI = [0.43, 0.72]; post hoc *t*-test in ambiguity context (averse > seeking): *t*(217) = 1.486, *p* = 0.139, Cohen’s *d* = 0.10, 95% CI = [−0.03, 0.23], Bayes Factor, BF_10_ = 0.86; Fig. 5c), demonstrating the robustness of the results.

In summary, as illustrated in Fig. 5d, people were more likely to be influenced to become more conservative rather than adventurous when the probability of uncertainty is clear (risk), while they were equally susceptible to others’ conservative and adventurous attitudes when the probability of uncertainty is ambiguous (ambiguity).

## Discussion

Across two experiments, we examined how observing others’ decisions influenced individuals’ risk (Exp. 1) and ambiguity (Exp. 2) preferences, respectively. Our findings revealed that participants changed their risk and ambiguity preferences to align with others, demonstrating significant social contagion effects pertaining both uncertainty preferences. These effects were also robust across decision frames (gain/loss) and observees’ uncertainty preferences (seeking/averse). Notably, the patterns of the contagion effect differed between the two uncertainty contexts. Ambiguity contagion was less asymmetric, with participants equally influenced by ambiguity-seeking and ambiguity-averse observees, whereas risk contagion was asymmetric, aligning stronger with risk-averse partners. Importantly, we replicated these findings in a preregistered replication study with an independent group of participants. These results may indicate distinct cognitive mechanisms of social influence on decision-making under different types of uncertainty.

Uncertainty preference plays a crucial role in guiding decisions, and our findings underscore its adaptive malleability to social influence. Consistent with the previous research^5^, our results showed that individuals’ risk preferences are susceptible to the influence of others’ behavior, irrespective of the decision contexts. Interestingly, we found a stronger contagion effect induced by observing risk-averse than risk-seeking choices in both gain and loss decision frames. Such an asymmetric social influence on risk preferences may reflect an intrinsic bias towards risk aversion. Risk aversion has been consistently identified in previous research^28,37,38^, and is thought to confer evolutionary advantages by keeping individuals from imminent dangers^39–42^. Despite being evolutionarily beneficial, risk-averse decisions often involve greater deliberation and inhibitory control, engaging higher-order cognitive processes^43,44^. Consistent with this account, reaction times decreased with increasing observer–observee distance after observing risk-seeking—but not risk-averse—choices, suggesting faster disengagement from risk-seeking social information but continued deliberative integration of risk-averse information. Accordingly, the asymmetric contagion effect may arise because risk-averse social information continues to elicit greater deliberative integration, leading to stronger alignment.

This study also revealed a similar social contagion effect on ambiguity preference. However, the contagion effect on ambiguity preferences is less asymmetric, in stark contrast to the risk contagion effect. We speculate that this difference might be due to the way people process information with different forms of uncertainty. Previous studies showed that people tend to explore more when faced with insufficient information to reduce uncertainty, but exploit the superior option when they possess sufficient information^45–47^. Here, in decisions under ambiguity, the probabilities of outcomes are unknown, compelling participants to sample information from others’ behavior. In the reinforcement learning paradigms, an exogenous bonus is usually assigned to the unknown option to encourage exploration to account for the actual human behaviors^48,49^. It is thus possible that the ostensible comparable update in the ambiguity preference was partly driven by subjects’ innate propensity to exploration, rather than a stronger inclination to learn from specific types of observees. Future studies should test this hypothesis by linking the exploration-exploitation trade-off to risk vs. ambiguity contagion and examining their potentially distinct neural mechanisms.

This study provides insights into the intrinsic differences between risk and ambiguity preferences, addressing a key challenge in comparing these two fundamental forms of uncertainty. Although previous research has indicated that risk and ambiguity preferences are behaviorally separable and associated with different neural substrates^23,24,26^, direct comparisons have been difficult due to differences in their measurement scales. Here, we provided an alternative avenue to tackle this challenge by perturbing one’s risk/ambiguity preference with social influence and focusing on the changes in risk/ambiguity preference relative to the baseline preference. This approach circumvents scale differences and enables a direct comparison. The identified asymmetric risk contagion and less asymmetric ambiguity contagion in the current study underscore the efficacy of this method, revealing distinct uncertainty preference update patterns in observational learning. Furthermore, a preregistered replication study using recorded choices from real participants yielded similar effects, consistent with previous studies that suggest social contagion operates similarly regardless of whether the source is a human or an algorithm^5,50^.

Previous studies have shown that individuals are strongly influenced by others perceived as similar, such as those who share common hobbies, preferences or perceptual biases^15,51^. This theory would predict participants adjusting their behaviors more closely to observees with similar behavioral preferences, i.e., a confirmation bias. However, our findings do not fully support this explanation. Participants tended to shift their preferences more towards individuals with dissimilar preferences (Fig. S6b and S9b), consistent with previous studies^5^. We propose that this asymmetric contagion effect in risk preference may reflect a conservative bias. This interpretation aligns with Ihssen et al., who found that participants in an observational learning task gave greater weight to others’ cautious choices than to impulsive ones when forming their own decisions^19^. Importantly, this conservative bias did not extend to ambiguity preferences in our study, suggesting a fundamental difference in how risk and ambiguity preferences are socially transmitted.

The exact cognitive mechanisms behind social contagion under uncertainty have been actively studied by various theoretical frameworks. For example, Chung et al.^6^ suggested that social contagion explicitly increased the perceived utility of others’ choices and led to social conformity behavior. In contrast, Suzuki et al.^5^ emphasized that observing others’ choices modified individuals’ uncertainty preferences through implicit observational learning. Other studies, however, suggest that participants have two copies of behavioral preferences along the executed-modeled dimension rather than the self-other dimension, and adaptively apply these preferences in a goal-directed manner^52^. The risk contagion effect can thus be viewed as the coactivation of both executed and modeled risk preferences.

The contagion effect may also arise from an integrated neural process. Recent neuroimaging studies link risk processing to the orchestrated activities in the dorsomedial prefrontal cortex (dmPFC), anterior insula, and ventral striatum^53^, while belief updating about others’ risk preferences involves brain areas such as the dorsolateral prefrontal cortex (dlPFC), with dlPFC-ventral striatum coupling linked to the risk contagion magnitude^5^. In contrast, studies on ambiguity decision-making emphasized the importance of the dlPFC and the orbitofrontal cortex (OFC) in representing ambiguity^23,26,53,54^. The difference in the neural representations of risk and ambiguity may drive distinct contagion patterns, while the activities in the overlapping brain areas may relate to social information integration in general.

Understanding social contagion will ultimately improve well-being and promote societal stability. In the current social media era, adolescents are especially vulnerable to social influence^9,10^. Our finding that people align more with risk-averse others suggests a potential avenue to deter high risk-taking behavior in adolescents by exposing them to more cautious peers. Furthermore, because contagion biases toward conservativeness appear specific to the risk context, transparent communication of risk information may be particularly important in domains such as financial markets^55^.

### Limitations

First, although we observed robust asymmetry in risk contagion, evidence regarding asymmetry in ambiguity contagion was inconclusive: Bayesian analyses provided indecisive support for the null hypothesis, and the observed effect size suggests that any asymmetry in ambiguity contagion, if it exists, may be small. Accordingly, the present findings highlight the difference of social influence across different types of uncertainty, with risk contagion showing stronger asymmetry than ambiguity contagion. Future work should further clarify the magnitude and boundary conditions of ambiguity contagion.

Second, our study adopted a one-way observation experimental design, and may not capture the full complexity of social influence, as real-life scenarios often involve bidirectional social communication. Bidirectional social interactions with reciprocal feedback, as shown by Mahmoodi et al., could involve mutual behavioral adjustment, and Kuroda et al. demonstrated that such dynamics could yield convergence toward stable, shared norms^51,56^. Incorporating such two-way paradigms in future research could increase the ecological validity of the social contagion effect of uncertainty preferences and shed light on how reciprocal influence shapes group decision-making and collective intelligence^57,58^. Finally, due to the experimental design, the present study was unable to capture social contagion effects in loss aversion, another important component influencing decisions under uncertainty. Future studies could directly examine how social contagion interacts with loss aversion by incorporating mixed gambles that allow independent estimation of both uncertainty (risk and ambiguity) and valence (loss aversion) preferences^29,59,60^.

In summary, our results revealed a risk-aversion bias in the contagion of risk preference, which was in stark contrast to the more symmetric contagion effect of ambiguity preference. These results highlight the nuanced interaction between social influence and uncertainty processing, offering insights that could inform interventions targeting cognitive biases in decision-making contexts.

## Supplementary information

Transparent Peer Review file

Supplemental Information

Reporting Summary

## Data Availability

The behavioral data collected in this work are available at https://osf.io/x2uwg/.

## Code availability

The code used to analyze the data in this work is available at https://osf.io/x2uwg/.
