# Supplementary material for: Distinct patterns of social contagion under risk and ambiguity
Source: Commun Psychol. 2026 Apr 10;4:94. doi: 10.1038/s44271-026-00452-5 (PMC13260373; doi:10.1038/s44271-026-00452-5)
Supplement: Supplementary file 2 — Supplemental Information [file 44271_2026_452_MOESM2_ESM.pdf]

Supplementary Figures

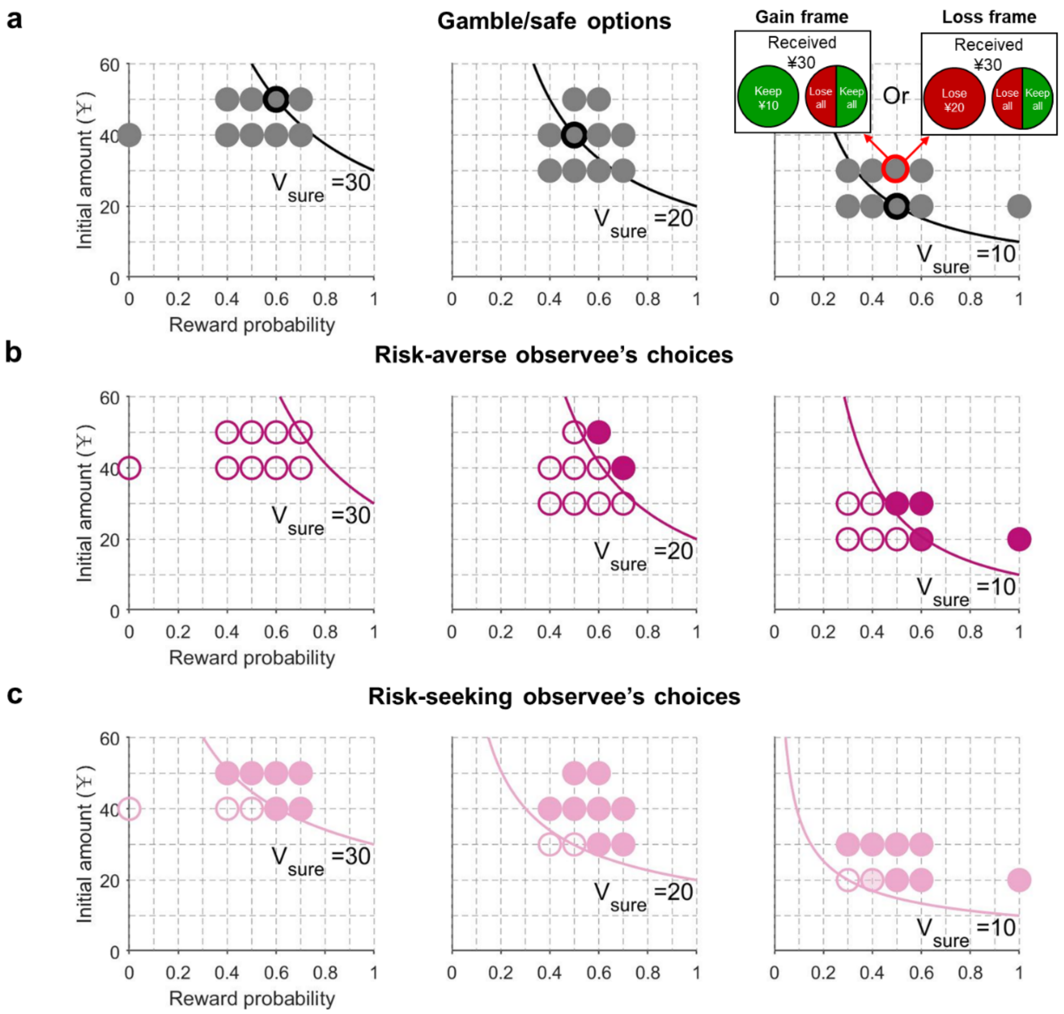

Figure S1. Options presented in Experiment 1 (Exp. 1) and the choice patterns of the two observees. (**a**) Three different types of sure options and sets of gambles that occurred with the sure options in *Self* trials are drawn into three graphs from left to right. V_{sure} indicates the value of reward participants will eventually get if they choose the sure option, regardless of the decision frame; values used in the experiment are 10, 20, and 30. Each point denotes one gamble characterized by the probability and the magnitude of the reward. The solid lines indicate the indifference curves, on which the utility of the gamble is equal to that of the sure option under the risk-neutral preference. To avoid the distortion of subjective probability proposed in Prospect Theory, we didn't use any probabilities smaller than 30%, except for the gambles used in two catch trials (P = 0 or 1). Dots with black edges denote gambles located on the indifference curve of their sure option counterpart and are presented twice in a random order for each block in *Predict* trials to examine the participants' learning performance about the Observees' risk-preference efficiently. **(b-c)** Choice patterns of the risk-averse and risk-seeking observees. The options presented in *Observe* trials are identical to those in *Self* trials. The solid lines represent the subjective

indifference curves for risk-averse observee (**b**) and risk-seeking observee (**c**), respectively. The graded color of each dot represents the probability that the observee chose the gamble (the filled color indicates 1; the transparent indicates 0). The risk-averse observee chose around 25% of gambles, while the risk-seeking observee chose around 75% of gambles. Also see Supplementary Table 1 for details.

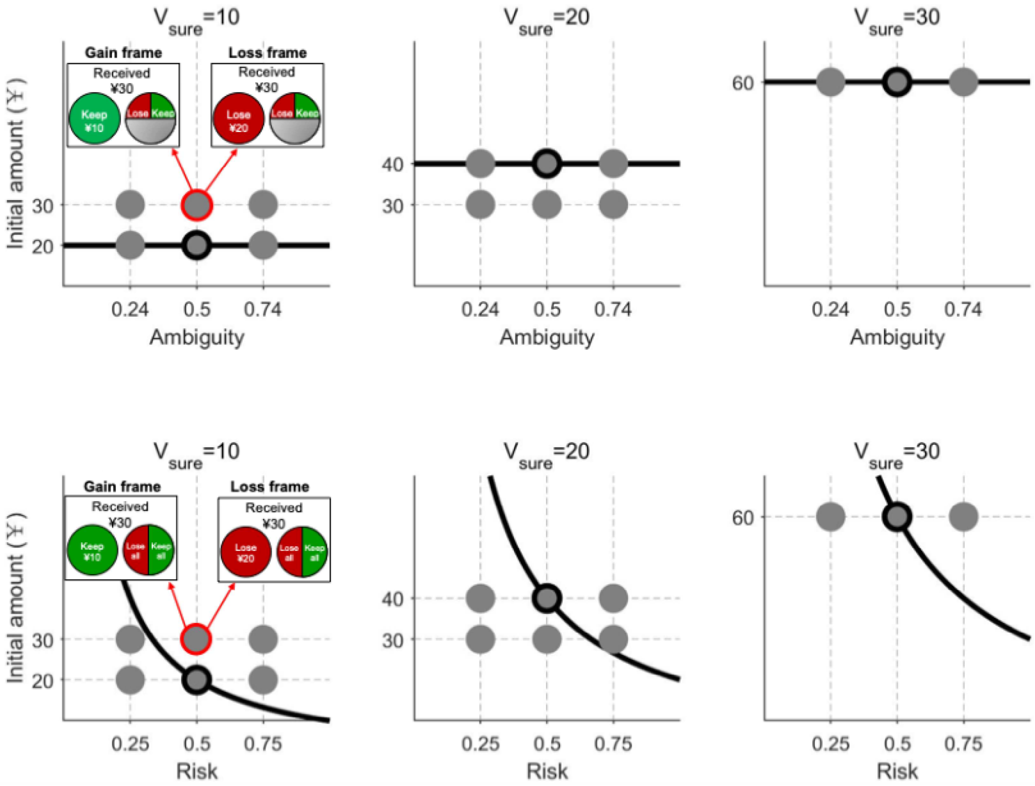

Figure S2. Options presented in Experiment 2 (Exp. 2). Three types of sure options and gambles occurred with the sure options in *Self* trials. The upper and lower panels show the pairs of sure options with ambiguous and risky gambles, respectively. V_{sure} indicates the value of reward participants will eventually get if they choose the sure option, regardless of the decision frame; values used in the experiment are 10, 20, and 30. Each point denotes one gamble characterized by the reward's probability and magnitude (Initial amount). The solid line indicates the indifference curve, on which the utility of the gamble is equal to that of the sure option under the ambiguity-neutral and risk-neutral preference. The indifference curves in the top panels are a flat line because the underlying probability of a gamble option is always 0.5 regardless of its ambiguity level. Dots with black edges denote gambles located on the indifference curve of their sure option counterpart and are presented twice in a random order for each block in *Predict* trials to examine the participants' learning performance about the Observees' risk- and ambiguity-preferences efficiently. Also see Supplementary Table 2 for details.

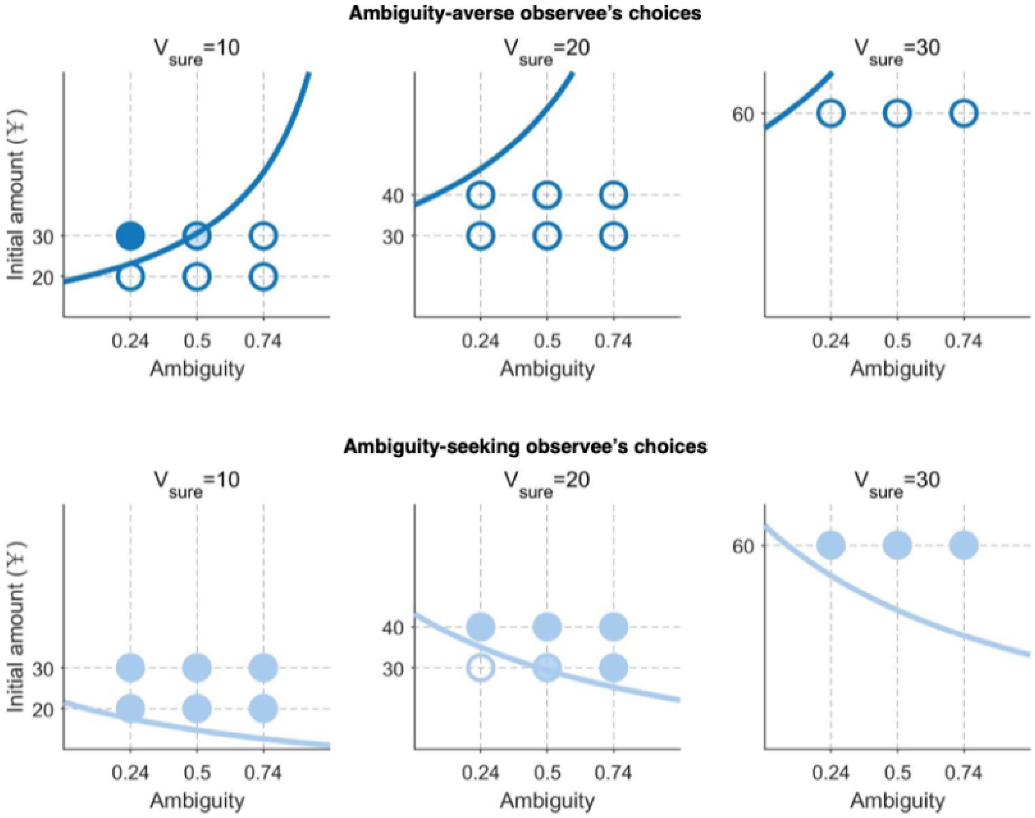

Figure S3. The observees' choice patterns in Exp. 2. The options presented in *Observe* trials are identical to those in *Self* trials. Choice patterns of the ambiguity-averse (chose less ambiguous gambles) and ambiguity-seeking (chose more ambiguous gambles) observees in ambiguous gambles are shown in the upper (blue) and lower (light blue) panels, respectively. Notably, the ambiguity-averse observee is also slightly risk-seeking, and the ambiguity-seeking observee is also slightly risk-averse. Each dot's graded transparency represents the probability that the observee chooses the gamble (1 for opaque; 0 for transparent). The solid line indicates the indifference curve, on which the utility of the gamble is equal to that of the sure option under the specific preference of an observee. Dots above the solid line are chosen by the observees, while the ones below it are unchosen. Also see Supplementary Table 2 for details.

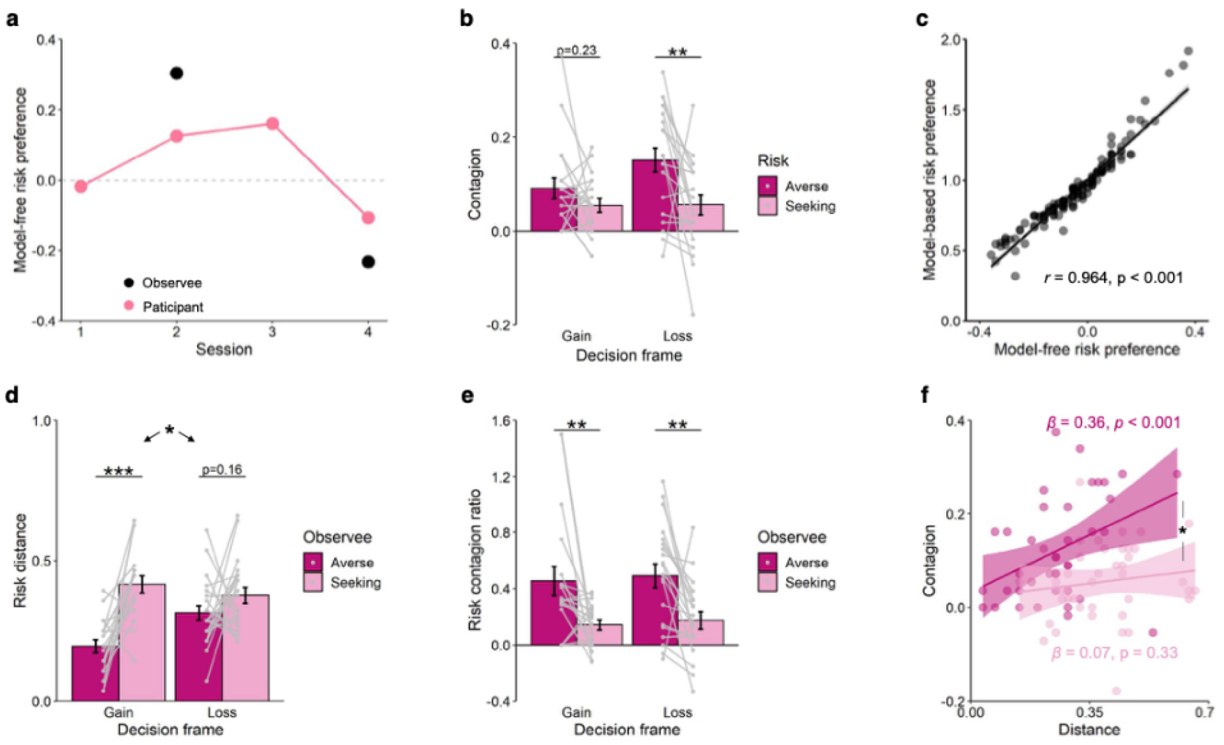

Figure S4. Model-free analysis of risk contagion effect. (**a**) A representative example of the risk contagion effect. The pink line denotes the participant's model-free risk preference (the proportion of choosing the gamble option relative to that chosen by a risk-neutral agent) changing across sessions. The two black dots indicate the observees' preference in Sessions 2 and 4. The participant shown in the current example is from the Gain frame. This example showed a clear pattern of risk contagion effect toward risk-averse and risk-seeking observees. (**b, d-e**) Mean model-free risk contagion (**b**), model-free risk distance between participants and observees (**d**), and model-free risk contagion ratio (**e**) as functions of decision frame and observees' risk-preference type. Mean ± SEM across participants within each group. Gray dots linked by a line represent each participant's data when observing risk-averse (magenta) and risk-seeking (pink) observees. These results are consistent with model-based analysis in Figure 2. (**c**) Scatter plot of participants' model-based risk preferences against the model-free risk preferences. Individuals' risk preferences are consistent between model-free and model-based measurements. The black line is fitted using a linear regression model (the shaded area indicates the 95% confidence interval). In (**e**), an ANOVA analysis revealed a consistent asymmetric risk contagion effect in both the gain and loss frames: subjects were more aligned with risk-averse observees than with risk-seeking ones ($F(1,37) = 17.257$, $p < 0.001$, $\eta_p^2 = 0.32$, 95% CI = [0.13, 1.00]). Neither the main effect of the decision frame ($F(1,37) = 0.218$, $p = 0.644$, $\eta_p^2 < 0.01$, 95% CI = [0.00, 1.00]) nor the frame × preference interaction effect ($F(1,37) = 0.001$, $p = 0.970$, $\eta_p^2 < 0.01$, 95% CI = [0.00, 1.00]) was statistically significant. (**f**) Model-free risk contagion effect as a function of risk distance. Each magenta/pink point represents a participant when observing risk-averse/risk-seeking observees. Fitted regression

lines are plotted, and the shaded areas around the fitted lines reflect the 95% CIs. The degree of contagion effect was also significantly correlated with the risk distance when the observee was risk-averse ($coef_{\text{risk-averse}}$ = 0.363 ± 0.101, $p < 0.001$), but not when the observee was risk-seeking ($coef_{\text{risk-averse}}$ = 0.070 ± 0.072, $p = 0.338$). ***$p < 0.001$; **$p < 0.01$; *$p < 0.05$; n.s., not significant.

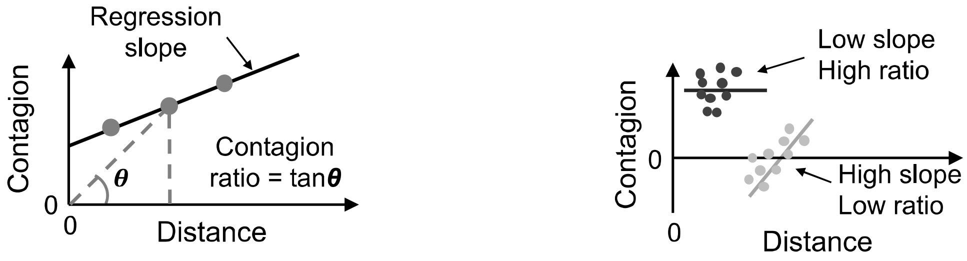

Figure S5. An illustration of contagion ratio analysis and regression analysis. Left panel: The contagion ratio measures individual-level contagion divided by distance (tanθ); The regression slope reflects the group-level tendency of how contagion is related to distance. Right panel: some made-up scenarios when two methods generate different results. If the individual-level contagion is large relative to the distance for most participants, the averaged contagion ratio will be high (dark gray dots). But, at the same time, it is possible that contagion does not increase with distance, so the regression slope is low (dark gray line). Another scenario is when the averaged contagion ratio is low (light gray dots), but the contagion still increases with distance at the group level (high slope, light gray line).

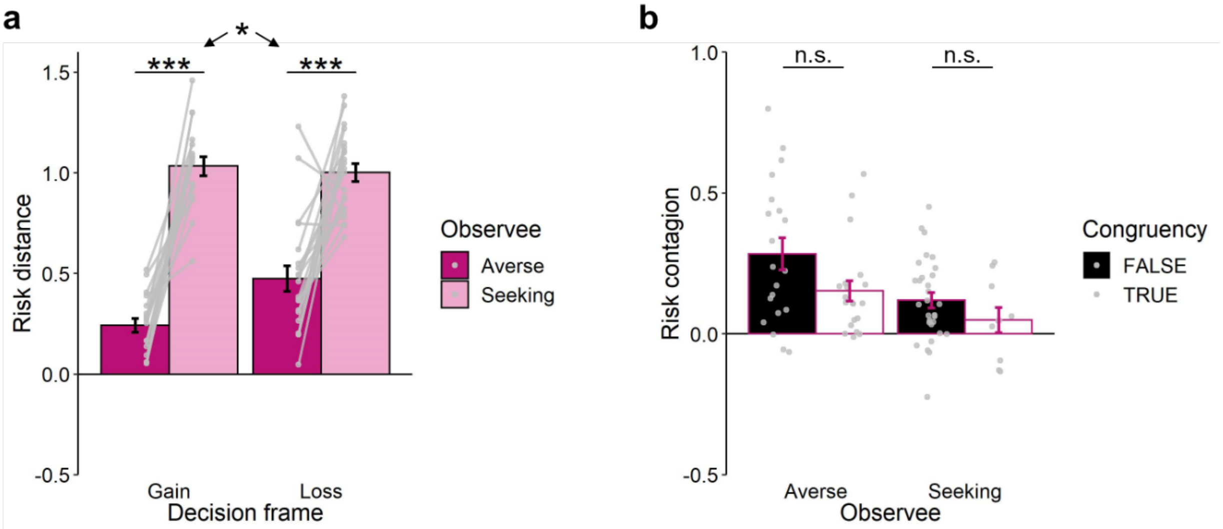

Figure S6. (**a**) Model-based risk distance between participants' and observees' risk preference as a function of decision frame and observees' risk-preference type (Mean ± SEM across participants within each group). Gray dots linked by a line represent the risk distance of each participant when observing risk-averse (magenta) and risk-seeking (pink) observees. ANOVA analysis revealed the main effect of the observee's risk preference type on risk distance ($F(1,38) = 131.06$, $p < 0.001$, $\eta_p^2 = 0.78$, 95% CI = [0.67, 1.00]) and the interaction effect ($F(1,38) = 5.39$, $p = 0.026$, $\eta_p^2 = 0.12$, 95% CI = [0.01, 1.00]) were significant. Post hoc analyses further revealed that participants had a smaller risk distance towards risk-averse than risk-seeking obervees in both the *Gain* (post hoc t-test: $t(38) = -9.572$, $p < 0.001$, Cohen's $d = -1.55$, 95% CI = [-2.02, -1.08]) and *Loss* frames (post hoc t-test: $t(38) = -6.695$, $p < 0.001$, Cohen's $d = -1.09$, 95% CI = [-1.48, -0.68]). (**b**) Risk contagion as a function of observees' risk-preference type and the congruency between the participant and the observee (Mean ± SEM across participants within each group). Gray dots represent the risk contagion of each participant when their risk preference is either congruent (white) or incongruent (black) with the observees. ANOVA analysis revealed neither the main effect of the congruency between the participants' and the observees' risk preference ($F(1,38) = 1.22$, $p = 0.276$, $\eta_p^2 = 0.03$, 95% CI = [0.00, 1.00]) nor the interaction effect ($F(1,38) = 0.08$, $p = 0.784$, $\eta_p^2 < 0.01$, 95% CI = [0.00, 1.00]) was significant. Post hoc analyses using the Wilcoxon rank sum test, given the unequal distribution of the data, revealed no effects of congruency when the observee was either risk-averse ($W = 255.5$, $p = 0.081$) or risk-seeking ($W= 237.5$, $p = 0.075$). ***$p < 0.001$; **$p < 0.01$; *$p < 0.05$; n.s., not significant.

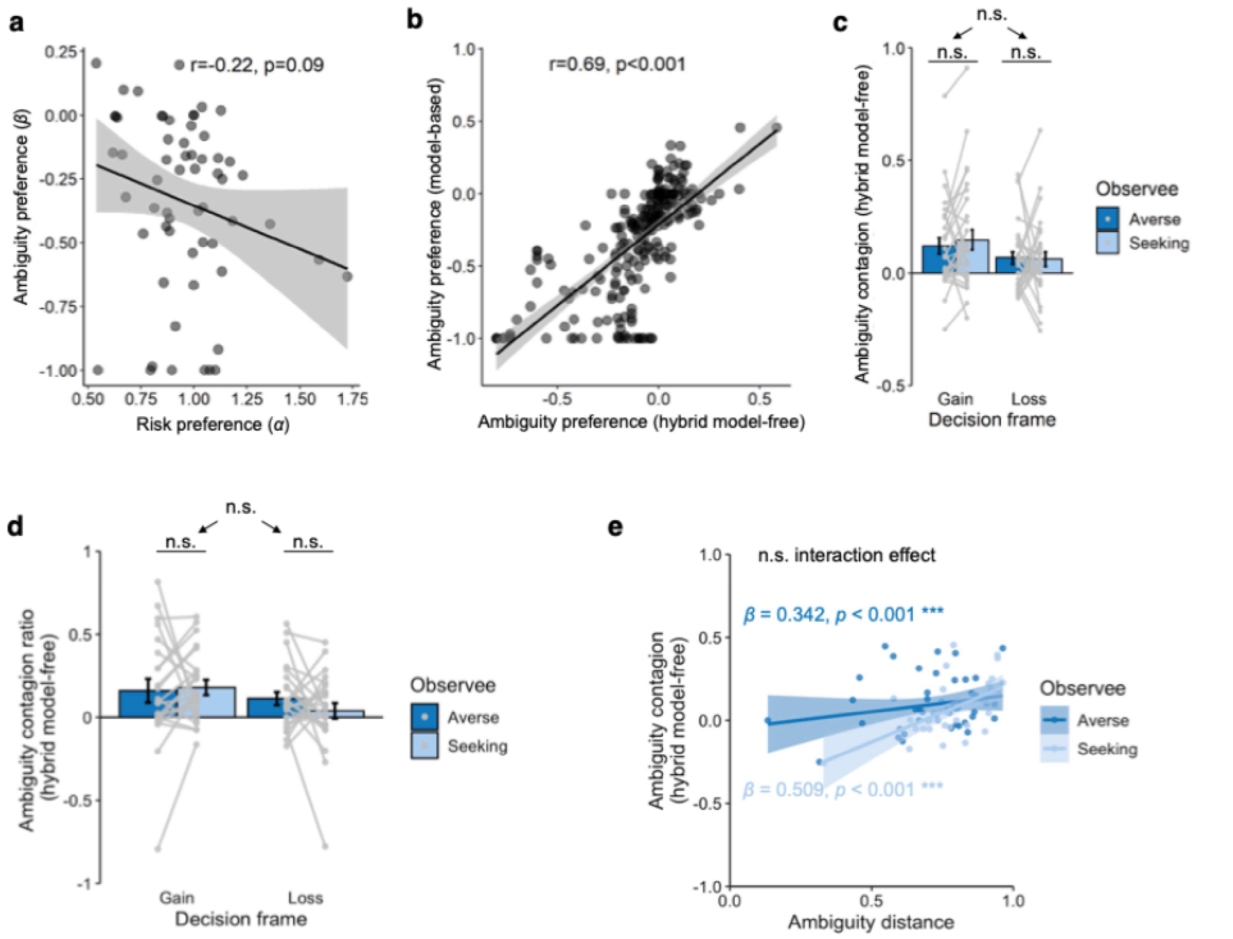

Figure S7. (**a**) Scatter plot of participants' estimated β (ambiguity preference) against α (risk preference) from the maxmin utility function in the first session. There is no significant correlation between individuals' risk and ambiguity preference, suggesting that the two preferences can be separated from each other. (**b**) Scatter plot of participants' model-based ambiguity preferences against the hybrid-model-free ambiguity preferences. The black line is fitted using a linear regression model (shaded area indicated the 95% CI). (**c**) Degree of hybrid-model-free ambiguity contagion as a function of decision frame and observees' ambiguity-preference type (mean ± SEM across participants within each group). Gray dots linked by a line represent the ambiguity contagion of each participant when observing ambiguity-averse and ambiguity-seeking observees. (**d**) Degree of hybrid-model-free ambiguity contagion ratio as a function of decision frame and observees' ambiguity-preference type. Extreme ratio values falling outside of three standard deviations were excluded to ensure a normal distribution. An ANOVA analysis on ambiguity contagion ratio showed that neither the observee's ambiguity preference ($F(1,46) = 0.449$, $p = 0.506$, $\eta_p^2 < 0.01$, 95% CI = [0.00, 1.00]), the decision frame ($F(1,46) = 2.618$, $p = 0.113$, $\eta_p^2 = 0.05$, 95% CI = [0.00, 1.00]), nor the interaction of these two variables affected the ambiguity contagion ratio ($F(1,46) = 1.114$, $p = 0.297$, $\eta_p^2 = 0.02$, 95% CI = [0.00, 1.00]), suggesting no reliable evidence for an asymmetric influence of observees' ambiguity preferences on participants'

ambiguity contagion. (**e**) Regression analysis with hybrid model-free ambiguity contagion effect as a function of ambiguity distance. Each blue/light-blue point represents a participant when observing ambiguity-averse/ambiguity-seeking observees. Fitted regression lines are plotted, and the shaded areas around the fitted lines reflect the 95% CIs. Specifically, the ambiguity contagion effect was positively correlated with ambiguity distance no matter when the observee was ambiguity-averse or ambiguity-seeking ($coef_{\text{ambiguity-averse}} = 0.342 \pm 0.095$, $p < 0.001$; $coef_{\text{ambiguity-seeking}} = 0.509 \pm 0.099$, $p < 0.001$; with no significant interaction effect between ambiguity distance and the observee's ambiguity-preference type: $coef_{\text{ambiguity-averse}} - coef_{\text{ambiguity-seeking}} = -0.168 \pm 0.138$, $p = 0.226$), indicating no reliable evidence for an asymmetric effect. ***$p < 0.001$; **$p < 0.01$; *$p < 0.05$; n.s., not significant.

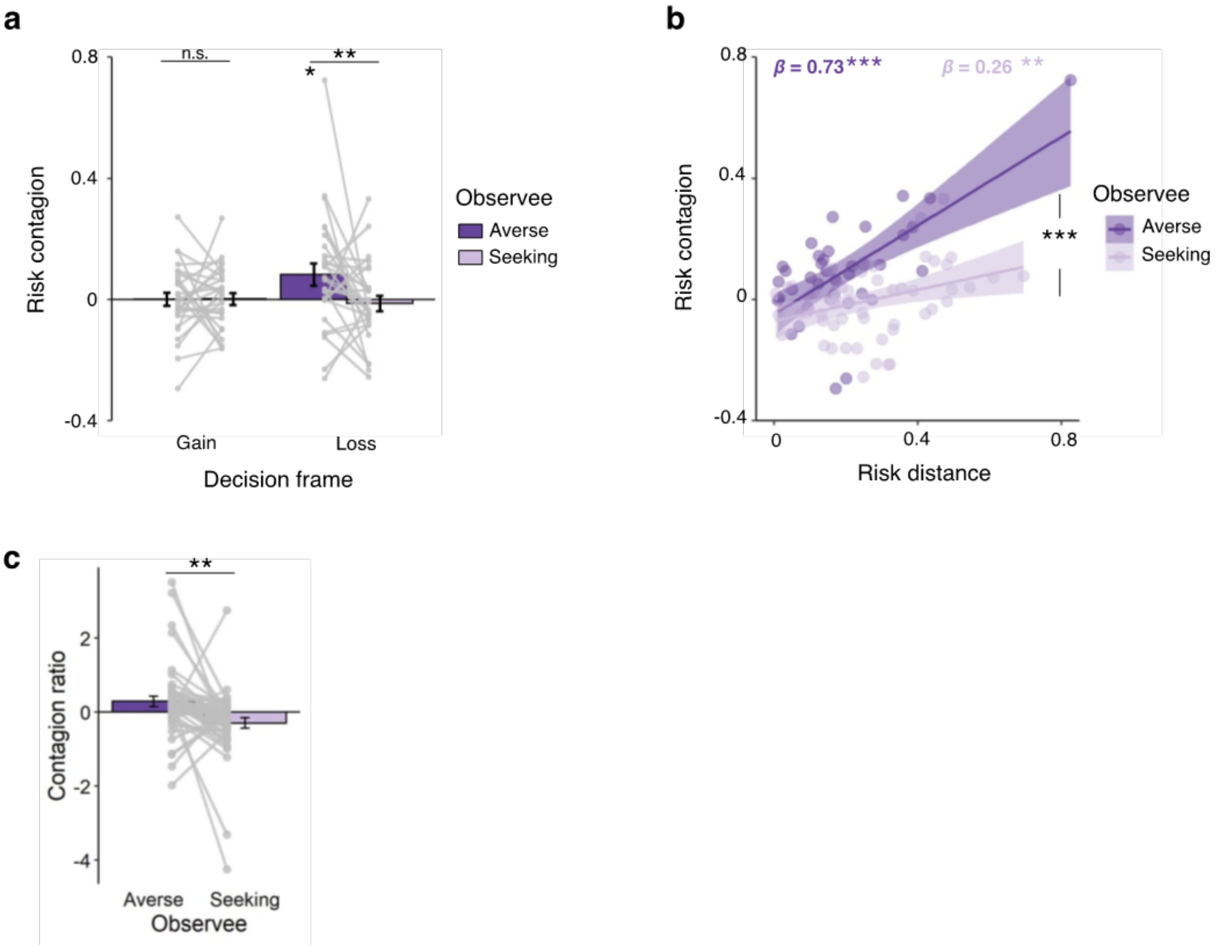

Figure S8. Risk contagion results in Exp. 2. (**a**) Degree of risk contagion as a function of decision frame and observees' risk-preference type (mean ± SEM across participants within each group). Gray dots linked by a line represent the risk contagion of each participant when observing risk-averse and risk-seeking observees, separately. The risk contagion effect was and only was significant when observing the risk-averse observee in the Loss frame ($t(27) = 2.249$, $p = 0.033$, Cohen's $d = 0.43$, 95% CI = [0.03, 0.82]). (**b**) Risk contagion is plotted as a function of the risk distance. Each purple/light-purple point represents a participant observing risk-averse/risk-seeking observees. Participants whose risk distances are below zero are excluded from the regression analysis. Fitted regression lines are plotted. Error bars reflect 95% CIs. (**c**) Risk contagion ratio as a function of observees' risk-preference type (mean ± SEM across participants within each group). The risk contagion ratio is significantly larger when observing the risk-averse than risk-seeking observees ($F(1,51) = 9.209$, $p = 0.004$, $\eta_p^2 = 0.15$, 95% CI = [0.03, 1.00]). Gray dots linked by a line represent the risk contagion ratio of each participant when observing risk-averse and risk-seeking observees, separately. ***$p < 0.001$; **$p < 0.01$; *$p < 0.05$; n.s., not significant.

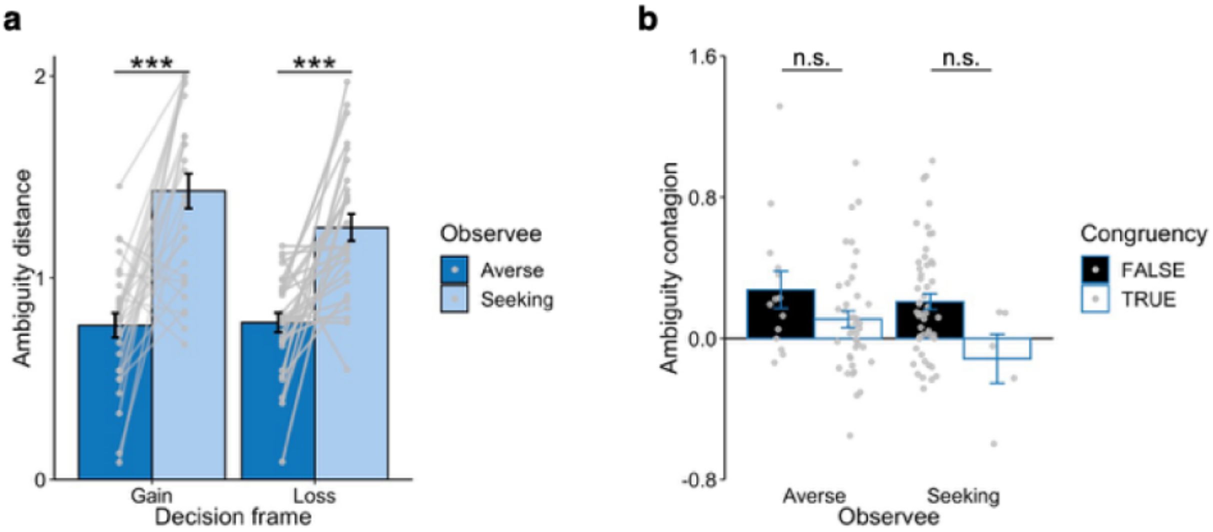

Figure S9. (**a**) Ambiguity distance as a function of decision frame and observees' ambiguity-preference type (mean ± SEM across participants within each group). Gray dots linked by a line represent the ambiguity contagion of each participant when observing ambiguity-averse (blue) and ambiguity-seeking (light blue) observees. (**b**) Ambiguity contagion as a function of observees' ambiguity preference type and the congruency between the participant and the observee (mean ± SEM across participants within each group). Gray dots represent the ambiguity contagion of each participant when their ambiguity preference is congruent (white) or incongruent (black) with the observees. ANOVA analysis revealed that the ambiguity contagion effect was also stronger when the preferences between participants and observees were incongruent (main effect of the congruency $F(1,53) = 4.29$, $p = 0.030$, $\eta_p^2 = 0.07$, 95% CI = [0.00, 1.00]), but the interaction effect was not significant ($F(1,53) = 0.149$, $p = 0.304$, $\eta_p^2 = 0.02$, 95% CI = [0.00, 1.00]). Post hoc analyses using the Wilcoxon rank sum test, given the unequal distribution of the data, revealed no effects of congruency when the observee was either ambiguity-averse ($W = 374$, $p = 0.134$) or ambiguity-seeking (W= 190, $p = 0.075$). ***$p < 0.001$; **$p < 0.01$; *$p < 0.05$; n.s., not significant.

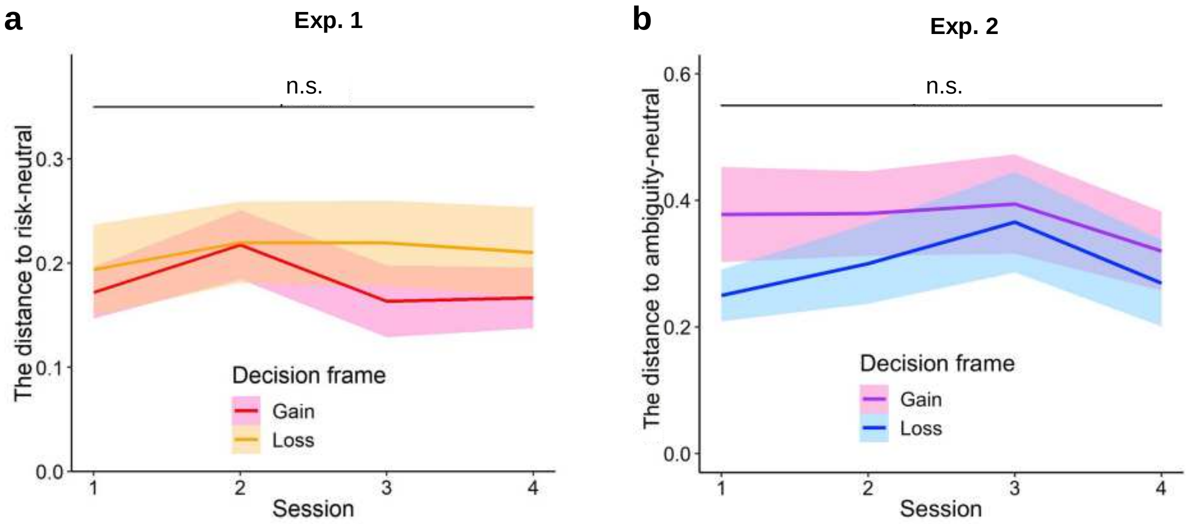

Figure S10. (**a**) Changes in the distance between participants' model-based risk preference and the hypothetical risk-neutral agent as a function of time in two decision frames (mean ± SEM across participants, Exp. 1). Participants didn't become more risk-neutral across sessions ($F(3,114) = 0.667$, $p = 0.574$, $\eta_p^2 = 0.02$, 95% CI = [0.00, 1.00]). (**b**) Changes in the distance between participants' model-based ambiguity-preference and the hypothetical ambiguity-neutral agent as a function of time in two decision frames (mean ± SEM across participants, Exp. 2). Participants didn't become more ambiguity-neutral across sessions ($F(3,162) = 0.661$, $p = 0.577$, $\eta_p^2 = 0.01$, 95% CI = [0.00, 1.00]).

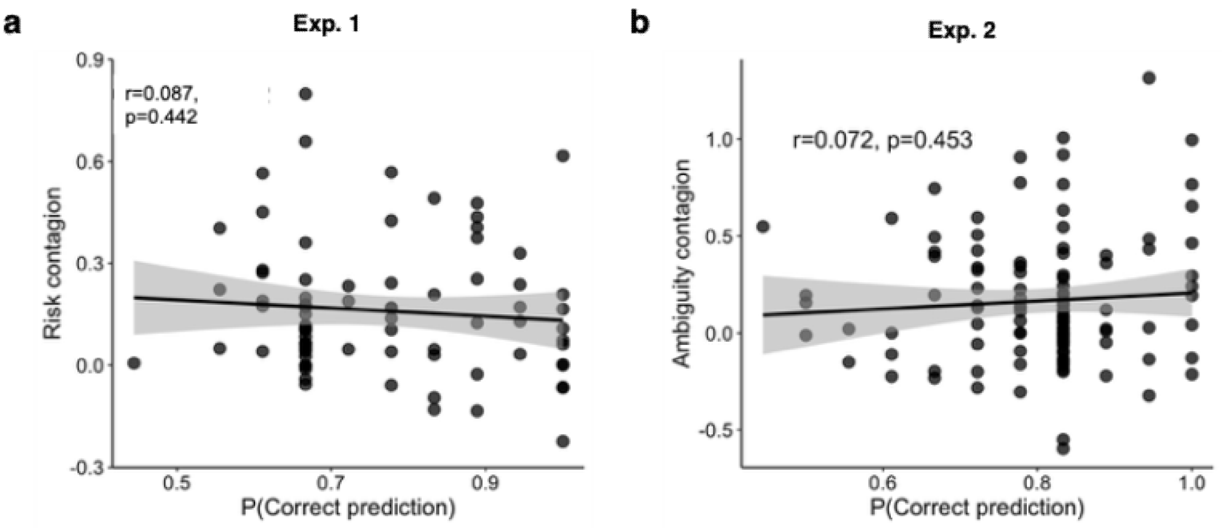

Figure S11. Scatter plots of model-based risk contagion (**a**) and ambiguity contagion (**b**) against the proportion of correct prediction in the *Predict* trials. The black lines are fitted using linear regression model (shaded areas indicate the 95% CIs). Each subject has two data points in each plot: one for observing the uncertainty-seeking observee and another for observing the uncertainty-averse observee.

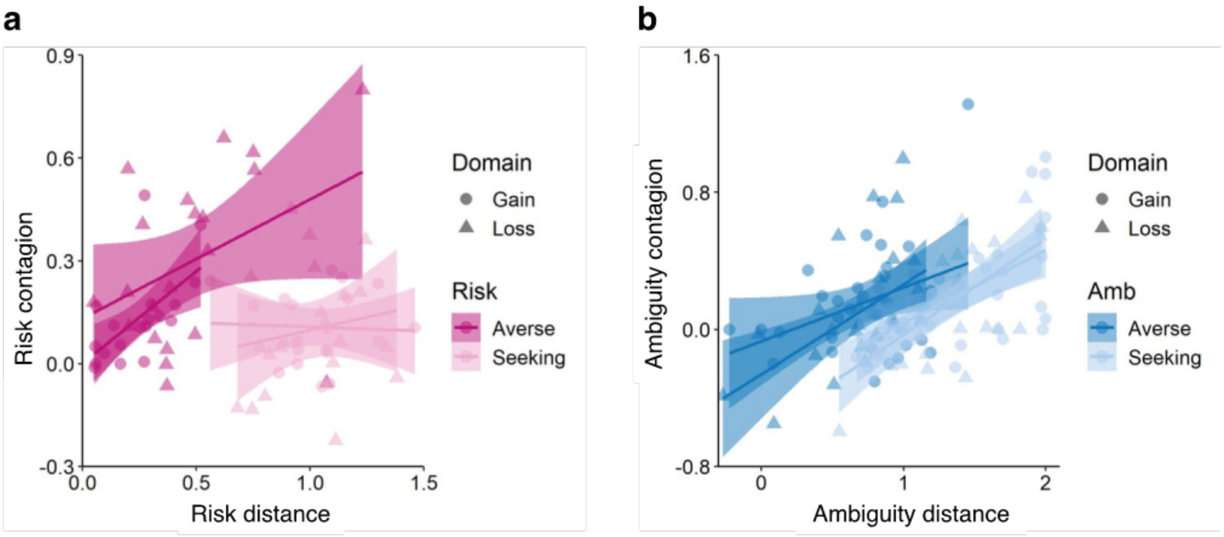

Figure S12. Model-based contagion effect as a function of preference distance, separated into different observee types and decision frames. (**a**) Risk contagion vs. risk distance in Exp. 1 and (**b**) ambiguity contagion vs. ambiguity distance in Exp. 2. Each point represents a participant observing uncertainty-averse/uncertainty-seeking observees in Gain/Loss frames. Different colors represent different observees, and different shapes represent different decision frames. Notably, there are no differences in regression results across decision frames in both subplots. Fitted regression lines are plotted. The shaded areas reflect 95% CIs.

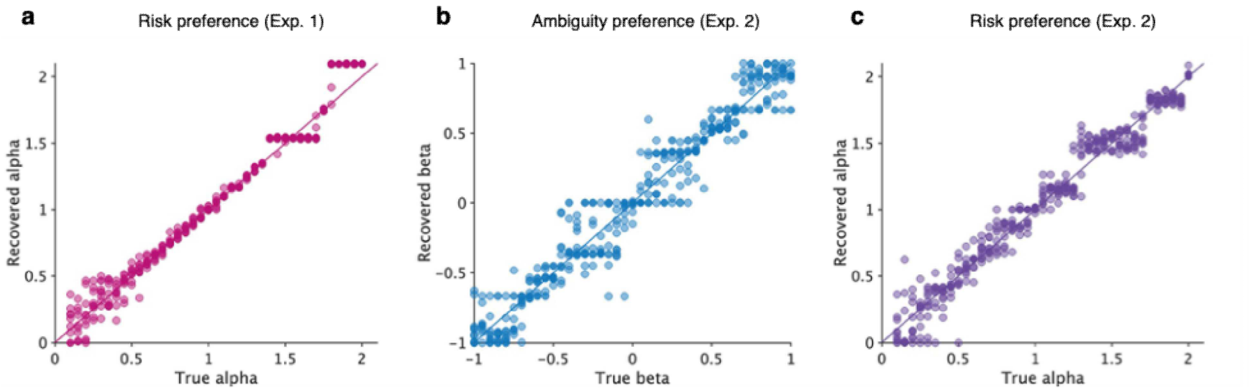

Figure S13. Parameter recovery. Recovered parameters from model fitting are plotted against the true parameters used to generate synthetic data. Each point represents one simulation. (**a**) Recovery of risk preference parameter α in Exp. 1. (**b**) Recovery of ambiguity preference parameter β in Exp. 2. (**c**) Recovery of the risk preference parameter α in Exp. 2. In the simulations of Exp. 2, risk preference was fixed at 1 (risk-neutral) when simulating ambiguity preference, and ambiguity preference was fixed at 0 (ambiguity-neutral) when simulating risk preference. For all simulations, inverse temperature values were randomly sampled between 1 and 10. In all cases, recovered values closely track the true values, demonstrating that the model reliably distinguishes between risk, ambiguity, and stochasticity parameters.

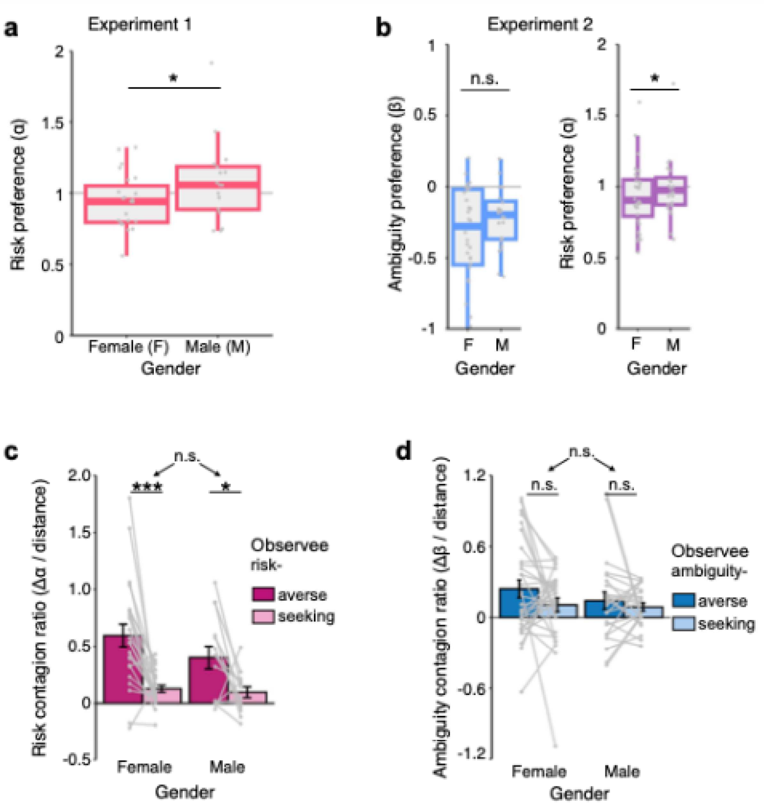

Figure S14. Gender difference in risk/ambiguity preferences and contagion effects. (**a**) Baseline of participants' risk preferences in Session 1 in Exp. 1. Female participants were initially more risk-averse than male participants (two-sample t-test: $t(37.712) = -2.439$, $p = 0.020$, Cohen's $d = -0.79$, 95% CI = [-1.45, -0.13]). Each gray dot indicates one individual's proportion of choosing the gamble option, and the box plot depicts the distribution among the group. The gray line in the middle indicates a hypothetical risk-neutral agent's risk preference. (**b**) Baseline of participants' ambiguity preferences (blue) and risk preferences (purple) in Session 1 in Exp. 2. While female participants were initially more risk-averse than male participants ($t(50.98) = -2.273$, $p = 0.031$, Cohen's $d = -0.64$, 95% CI = [-1.20, -0.07]), there was no gender difference in initial ambiguity preference ($t(50.98) = -0.314$, $p = 0.755$, Cohen's $d = -0.09$, 95% CI = [-0.64, 0.46]). (**c**) Risk contagion ratio is plotted as a function of the participant's gender and observees' risk preference. There was a consistent asymmetric risk contagion effect in both the male and female participants: participants were more aligned with risk-averse observees than with risk-seeking ones ($F(1,35) = 30.329$, $p < 0.001$, $\eta_p^2 = 0.46$, 95% CI = [0.26, 1.00]). Neither the main effect of the gender ($F(1,35) = 1.868$, $p = 0.180$, $\eta_p^2 = 0.05$, 95% CI = [0.00, 1.00]) nor the interaction effect ($F(1,35) = 1.813$, $p = 0.284$, $\eta_p^2 = 0.05$, 95% CI = [0.00, 1.00]) was statistically significant, suggesting no gender difference in risk contagion effects. Gray dots linked by a line represent the risk contagion ratio of each participant when observing risk-averse (magenta) and risk-seeking (pink) observees. Error bars represent the SEM. (**d**) Degree of ambiguity contagion ratio as a function of the participant's gender and the observee's ambiguity-preference type (mean ± SEM). Gray dots linked by a line

represent the ambiguity contagion ratio of each participant when observing ambiguity-averse and ambiguity-seeking observees. ANOVA analysis on ambiguity contagion ratio showed that neither the observee’s ambiguity preference ($F(1,52) = 0.73$, $p = 0.397$, $\eta_p^2 = 0.01$, 95% CI = [0.00, 1.00]), participants’ gender ($F(1,52) = 2.774$, $p = 0.102$, $\eta_p^2 = 0.05$, 95% CI = [0.00, 1.00]), nor the interaction effects affected the ambiguity contagion ratio ($F(1,52) = 0.462$, $p = 0.500$, $\eta_p^2 < 0.01$, 95% CI = [0.00, 1.00]). ***$p < 0.001$; *$p < 0.05$; n.s., not significant.

Supplementary Table 1. Task design and observees' choices in Experiment 1 (risk contagion).

Initial value	Gamble option	Sure option		Observees choosing gamble	
	Probability (keep all)	Keep (Gain Frame)	Lose (Loss Frame)	risk-averse	risk-seeking
20	0.3	10	10	0	0
20	0.4	10	10	0	1
20	0.5	10	10	0	1
20	0.6	10	10	0	1
20	1	10	10	1	1
30	0.3	10	20	0	1
30	0.4	10	20	0	1
30	0.4	20	10	0	0
30	0.5	10	20	1	1
30	0.5	20	10	0	0
30	0.6	10	20	1	1
30	0.6	20	10	0	0
30	0.7	20	10	0	1
40	0	30	10	0	0
40	0.4	20	20	0	1
40	0.4	30	10	0	0
40	0.5	20	20	0	1
40	0.5	30	10	0	0
40	0.6	20	20	0	1
40	0.6	30	10	0	1
40	0.7	20	20	1	1
40	0.7	30	10	0	1
50	0.4	30	20	0	1
50	0.5	20	30	0	1
50	0.5	30	20	0	1
50	0.6	20	30	1	1
50	0.6	30	20	0	1
50	0.7	30	20	0	1

Supplementary Table 2. Task design and observees' choices in Experiment 2 (ambiguity contagion).

Initial value	Gamble option		Sure option		Observees choosing gamble option	
	Probability (keep all)	Ambiguity	Keep (Gain)	Lose (Loss)	ambiguity-averse (slightly risk-seeking)	ambiguity-seeking (slightly risk-averse)
20	0.25	0	10	10	0	0
30	0.25	0	10	20	0	0
30	0.25	0	20	10	0	0
40	0.25	0	20	20	0	0
60	0.25	0	30	30	0	0
20	0.5	0	10	10	1	0
30	0.5	0	10	20	1	1
30	0.5	0	20	10	0	0
40	0.5	0	20	20	1	0
60	0.5	0	30	30	1	0
20	0.75	0	10	10	1	1
30	0.75	0	10	20	1	1
30	0.75	0	20	10	1	1
40	0.75	0	20	20	1	1
60	0.75	0	30	30	1	1
20	0.5	0.24	10	10	0	1
30	0.5	0.24	10	20	1	1
30	0.5	0.24	20	10	0	0
40	0.5	0.24	20	20	0	1
60	0.5	0.24	30	30	0	1
20	0.5	0.5	10	10	0	1
30	0.5	0.5	10	20	0	1
30	0.5	0.5	20	10	0	1
40	0.5	0.5	20	20	0	1
60	0.5	0.5	30	30	0	1
20	0.5	0.74	10	10	0	1
30	0.5	0.74	10	20	0	1
30	0.5	0.74	20	10	0	1
40	0.5	0.74	20	20	0	1
60	0.5	0.74	30	30	0	1
