# Supplementary material for: Distinct patterns of social contagion under risk and ambiguity
Source: Commun Psychol. 2026 Apr 10;4:94. doi: 10.1038/s44271-026-00452-5 (PMC13260373; doi:10.1038/s44271-026-00452-5)
Supplement: Supplementary file 3 — Reporting Summary [file 44271_2026_452_MOESM3_ESM.pdf]

nature portfolio

Corresponding author(s): Qingtian Mi (qingtian.mi@psy.ox.ac.uk)
Jian Li (li.jian@pku.edu.cn)

Last updated by author(s): Mar 9, 2026

Reporting Summary

Nature Portfolio wishes to improve the reproducibility of the work that we publish. This form provides structure for consistency and transparency in reporting. For further information on Nature Portfolio policies, see our Editorial Policies and the Editorial Policy Checklist.

Statistics

For all statistical analyses, confirm that the following items are present in the figure legend, table legend, main text, or Methods section.

n/a	Confirmed	
☐	☒	The exact sample size (*n*) for each experimental group/condition, given as a discrete number and unit of measurement
☐	☒	A statement on whether measurements were taken from distinct samples or whether the same sample was measured repeatedly
☐	☒	The statistical test(s) used AND whether they are one- or two-sided<br>*Only common tests should be described solely by name; describe more complex techniques in the Methods section.*
☐	☒	A description of all covariates tested
☐	☒	A description of any assumptions or corrections, such as tests of normality and adjustment for multiple comparisons
☐	☒	A full description of the statistical parameters including central tendency (e.g. means) or other basic estimates (e.g. regression coefficient) AND variation (e.g. standard deviation) or associated estimates of uncertainty (e.g. confidence intervals)
☐	☒	For null hypothesis testing, the test statistic (e.g. *F*, *t*, *r*) with confidence intervals, effect sizes, degrees of freedom and *P* value noted<br>*Give P values as exact values whenever suitable.*
☒	☐	For Bayesian analysis, information on the choice of priors and Markov chain Monte Carlo settings
☐	☒	For hierarchical and complex designs, identification of the appropriate level for tests and full reporting of outcomes
☐	☒	Estimates of effect sizes (e.g. Cohen's *d*, Pearson's *r*), indicating how they were calculated

Our web collection on statistics for biologists contains articles on many of the points above.

Software and code

Policy information about availability of computer code

Data collection	The behavioral data were collected in person at the School of Psychological and Cognitive Sciences, Peking University, using MATLAB-coded program. Experiment scripts are accessible on the Open Science Framework (https://osf.io/x2uwg/).
Data analysis	Data were analyzed using MATLAB (2017; The Mathworks, Natick, MA) and R (R Development Core Team, 2008). All scripts are accessible on the Open Science Framework (https://osf.io/x2uwg/).

For manuscripts utilizing custom algorithms or software that are central to the research but not yet described in published literature, software must be made available to editors and reviewers. We strongly encourage code deposition in a community repository (e.g. GitHub). See the Nature Portfolio guidelines for submitting code & software for further information.

Data

Policy information about availability of data

All manuscripts must include a data availability statement. This statement should provide the following information, where applicable:

- Accession codes, unique identifiers, or web links for publicly available datasets
- A description of any restrictions on data availability
- For clinical datasets or third party data, please ensure that the statement adheres to our policy

Data and materials are accessible on the Open Science Framework (https://osf.io/x2uwg/).

Research involving human participants, their data, or biological material

Policy information about studies with human participants or human data. See also policy information about sex, gender (identity/presentation), and sexual orientation and race, ethnicity and racism.

Reporting on sex and gender	Participants' sex was collected through self-report. We reported the proportions of female and male participants (please see Methods section for details). Neither sex nor gender was included as a variable in any analyses.
Reporting on race, ethnicity, or other socially relevant groupings	Participants' race was collected through self-report. All participants were Chinese students at Peking University. Neither race nor ethnicity was included as a variable in any analyses.
Population characteristics	Participants were students at Peking University. All participants were right-handed and reported having normal or corrected-to-normal eye vision, no colorblindness, and no history of neurological or psychiatric illnesses.<br><br>Original Study.<br>Experiment 1. Exp. 1 included 40 participants. 19 participants were randomly allocated to the Gain decision frame (11 females and 8 males; age:19.10 ± 1.44 y, mean ± SD), whereas the other 21 were in the Loss decision frame (9 females and 12 males; 19.05 ± 1.58 y).<br>Experiment 2. Exp. 2 included 56 participants. 28 participants were randomly assigned to the Gain decision frame (15 females and 13 males; 20.78 ± 2.40 y) and the other 28 participants were in the Loss decision frame (17 females and 11 males; 21.43 ± 2.11 y).<br><br>Participants in the replication study<br>Experiment 1. The replication of Exp. 1 included 61 participants. 31 participants were randomly allocated to the Gain decision frame (15 females and 16 males; 22.45 ± 2.46 y), whereas the other 30 were in the Loss decision frame (14 females and 16 males; 21.30 ± 2.56 y).<br>Experiment 2. The replication of Exp. 2 included 62 participants. 32 participants were randomly allocated to the Gain decision frame (15 females and 17 males; 21.84 ± 3.28 y), whereas the other 30 were in the Loss decision frame (16 females and 14 males; 22.40 ± 2.22 y).
Recruitment	Participants were recruited from the psychological experiments subject pool at the University via web advertisements.
Ethics oversight	Ethics Committee of the Peking University.

Note that full information on the approval of the study protocol must also be provided in the manuscript.

Field-specific reporting

Please select the one below that is the best fit for your research. If you are not sure, read the appropriate sections before making your selection.

☐ Life sciences ☒ Behavioural & social sciences ☐ Ecological, evolutionary & environmental sciences

For a reference copy of the document with all sections, see nature.com/documents/nr-reporting-summary-flat.pdf

Behavioural & social sciences study design

All studies must disclose on these points even when the disclosure is negative.

Study description	The study analyzed quantitative data, mainly from an experimental mixed design with between-subject factors (decision contexts) and within-subject factors (observees' uncertainty preferences).
Research sample	As described above, participants were Chinese students at Peking University.
Sampling strategy	Original Study.<br>The sample size in Experiment 1 was determined a priori using G*Power (Faul et al., 2009). Based on a one-sample t-test about the contagion effect in the previous study (Suzuki et al., 2016), we calculated a required minimum of 15 participants per condition, assuming a power of 0.8 and α = 0.05. As there are no prior study, in experiment 2, we doubled the minimum number of participants required for Experiment 1 to ensure adequate statistical power.<br><br>Replication Study.<br>Experiment 1. We first calculated the effect size of the asymmetric risk contagion effect observed in the original Exp. 1: Cohen's d = 0.9031. Based on this, we then used G*Power to estimate that the minimum number of participants required per condition is 10 in total, assuming a statistical power of 0.8 and α = 0.05. Although this analysis suggests a relatively small sample size, we planned to collect a larger sample due to concerns over replication reliability in studies with small sample sizes.<br>Experiment 2. To ensure that the absence of a statistically significant asymmetry for ambiguity contagion effectwas not due to insufficient statistical power, we calculated the effect size of the asymmetric ambiguity contagion effect (if it exists) observed in the original Exp. 2 (effect size: Cohen's d = 0.228). A sensitivity analysis (G*Power, two-tailed t-test, α = 0.05, power = 0.8) revealed that detecting an effect of this small magnitude would require approximately 154 participants per condition—a sample size beyond

practical feasibility. To further confirm this absence of asymmetric effect, we plan to recruit at least 30 valid participants per condition in the Replication of Exp. 2, after exclusion.

Data collection	Data were collected at the room 1315 Wang Kezhen Building, Peking University. Visual stimuli were coded in MATLAB using the Psychtoolbox extension (Brainard, 1997) on a computer with Windows 10 system. They were displayed on an LCD monitor (screen size 27 inches) with a resolution of 1280 × 1024 pixels at a frame rate of 144 Hz. Participants' responses were recorded by the input of keyboard connected to the computer. Participants were told that the observee's behaviors were made by another naive participant in the previous experiment. The observees' photos were taken from the back in the same experiment room. Besides, the experimenter also took a photo of the participant from the back at the beginning of the experiment and told them that future subjects might observe the record of their choices. By doing so, we tried to convince participants that the choices they observed were made by real people, and this was debriefed after the experiment.
Timing	Original Study: Experiment 1 (from September to October, 2019); Experiment 2 (from March to May, 2021)<br>Replicate Study: Experiment 1 (from April to May 2025); Experiment 2 (from May to June, 2025)
Data exclusions	Five participants from Experiment 1 were excluded because of their extreme choice patterns (e.g., never chose gamble options across four sessions) or predicted observees' choices poorly (the prediction accuracy failed to exceed the 50% chance level after observational learning in at least one session). Six participants from Experiment 2 were excluded because of their extreme choice patterns or ill performance in the prediction phases (similar to Exp. 1).<br><br>Pre-registered exclusion criteria for replication study. Participants were excluded from the whole data analyses if they met any of the following: (1) low prediction accuracy in Predict trials after the observation in at least one session (threshold = 50% chance level), (2) extremely biased or invariant choices, indicating a lack of meaningful engagement (e.g., always choosing gamble or always choosing sure option), (3) explicit disbelief in the experimental setting or suspicion about the manipulation, as revealed during debriefing, and (4) fail to pass the attention check (i.e., catch trials). Moreover, for the calculation of the contagion ratio (defined as the contagion effect divided by the preference distance between the participants and the observee), participants with zero or negative distance toward the observee (same preference as the observee or even more extreme) were excluded from the analyses. All exclusion criteria were pre-registered (https://osf.io/f4wuy). We excluded eight participants based on pre-registered exclusion criteria for Experiment 1 (valid sample size = 61). We excluded seven participants based on pre-registered exclusion criteria for Experiment 2 (valid sample size = 62).
Non-participation	No participants dropped out after the study started
Randomization	Participants were randomly assigned to different groups

Reporting for specific materials, systems and methods

We require information from authors about some types of materials, experimental systems and methods used in many studies. Here, indicate whether each material, system or method listed is relevant to your study. If you are not sure if a list item applies to your research, read the appropriate section before selecting a response.

Materials & experimental systems

n/a	Involved in the study
☒	☐ Antibodies
☒	☐ Eukaryotic cell lines
☒	☐ Palaeontology and archaeology
☒	☐ Animals and other organisms
☒	☐ Clinical data
☒	☐ Dual use research of concern
☒	☐ Plants

Methods

n/a	Involved in the study
☒	☐ ChIP-seq
☒	☐ Flow cytometry
☒	☐ MRI-based neuroimaging

Plants

Seed stocks	*Report on the source of all seed stocks or other plant material used. If applicable, state the seed stock centre and catalogue number. If plant specimens were collected from the field, describe the collection location, date and sampling procedures.*
Novel plant genotypes	*Describe the methods by which all novel plant genotypes were produced. This includes those generated by transgenic approaches, gene editing, chemical/radiation-based mutagenesis and hybridization. For transgenic lines, describe the transformation method, the number of independent lines analyzed and the generation upon which experiments were performed. For gene-edited lines, describe the editor used, the endogenous sequence targeted for editing, the targeting guide RNA sequence (if applicable) and how the editor was applied.*
Authentication	*Describe any authentication procedures for each seed stock used or novel genotype generated. Describe any experiments used to assess the effect of a mutation and, where applicable, how potential secondary effects (e.g. second site T-DNA insertions, mosiacism, off-target gene editing) were examined.*
