# Supplementary material for: Distinct patterns of social contagion under risk and ambiguity
Source: Commun Psychol. 2026 Apr 10;4:94. doi: 10.1038/s44271-026-00452-5 (PMC13260373; doi:10.1038/s44271-026-00452-5)
Supplement: Supplementary file 1 — Transparent Peer Review file [file 44271_2026_452_MOESM1_ESM.pdf]

nature portfolio

Peer Review File

Distinct patterns of social contagion under risk and ambiguity

Corresponding Author: Mr Deng Pan

This file contains all editorial decision letters in order by version, followed by all author rebuttals in order by version.

Version 0:

Decision Letter:

Dear Mr Pan,

Thank you for your patience during the peer-review process. Your manuscript titled "Asymmetric social influence on risk but not ambiguity preference in decision-making under uncertainty" has now been seen by 3 reviewers, whose comments are appended below. I have discussed the reports with my colleagues and I regret to inform you that we decided that in light of the referee reports, we cannot publish your manuscript in Communications Psychology.

You will see that the reviewers raise substantive concerns. Taking these points together with our editorial considerations, these reservations preclude publication of this study in Communications Psychology.

I am sorry that we cannot be more positive on this occasion and thank you for the opportunity to consider your work.

Best regards,

Yafeng Pan

Yafeng Pan, PhD
Editorial Board Member
Communications Psychology
orcid.org/0000-0002-5633-8313

REVIEWERS' EXPERTISE:
Reviewer #1: decision making, computational modelling
Reviewer #2: decision making, computational modelling
Reviewer #3: decision making, computational modelling

REVIEWER COMMENTS:

Reviewer #1 (Remarks to the Author):

Paper summary:

This paper investigates the impact of social contagion on individuals' risk and ambiguity preferences. Specifically, it examines how a person's attitude toward risk (or ambiguity) is influenced by observing someone else choosing either a risk-averse (or ambiguity-averse) or risk-seeking (or ambiguity-seeking) option. The findings provide evidence of a social contagion effect: participants adjusted their risk attitudes in the direction of the observed behavior. Notably, the shift was stronger in the risk-averse direction than in the risk-seeking direction. In contrast, the contagion effect on ambiguity attitudes was symmetric, with participants equally influenced by both ambiguity-averse and ambiguity-seeking behaviors.

Comments:

1. I can see the authors have done power calculations in the Appendix. But I still feel the sample size of the experiment is too

small. It would really be great if authors are able to do a new study with a sufficiently larger sample size and replicate the findings.

2. I can see that the subjects go through many trials in both the experiments. Why then collapse the data and do an ANOVA. Wouldn't a panel data analysis that treats each data point independently be better?

3. Is the asymmetric social contagion effect towards risk aversion due to the fact that risk seeking choices of the observee happen in the earlier block (block 2) while risk averse choices of the observee happen in the later block *block 4)? Could this also be explained by the reaction time data?

4. What does the reaction time data tell about the mechanism?

5. There is deception involved in the experiment where the authors tell the subjects that they observe the choice of a real subject, when they don't. I hope the subjects were debriefed about this at the end of the experiment. Could this be done in real by providing an actual choice of a risk averse subject ? See for example Narayanan et al. (2024), which has also found that decision makers tend to anchor on algorithmic advice that contrast with their own risk attitudes.

6. I am surprised that the asymmetric contagion effect of risk attitudes do not happen in the second experiment when the risk attitudes are disintegrated out in the model. What is the reason?

7. In Figure 4d, directionally the effects are very similar to that of the risk case. They are not significant though. Would it be that with higher sample size there could also be an asymmetric social contagion towards ambiguity aversion similar to the risk averse case? This is the reason why an experiment with larger sample size would be useful to test the robustness of the findings.

8. In Figure 3b, why is the correct prediction of risk behavior lower for the middle and end trials, when it should typically be the opposite?

9. Minor: In the introduction "For instance, when choosing between a small, sure reward and a larger reward
45 associated with uncertainty, an uncertainty-averse individual will typically select the sure one"

This is not necessarily the case, unless the expected value of both the options are the same.

10. Ambiguity attitudes in Experiment 2 is muddled with risk attitudes. To measure ambiguity attitudes more precisely, it might make sense to make subjects choose between a risky and an ambiguous prospect instead of an ambiguous prospect and sure outcome (see also Dimmock et al 2016).

References

1. Narayanan, P., Somasundaram, J., & Seifert, M. (2025). Risk-averse algorithmic support and inventory management. European Journal of Operational Research, 322(3), 993-1004.

2. Dimmock, S. G., Kouwenberg, R., & Wakker, P. P. (2016). Ambiguity attitudes in a large representative sample. Management Science, 62(5), 1363-1380.

Reviewer #2 (Remarks to the Author):

Summary

Previous studies have demonstrated the social contagion effect in risky decision-making. Yet it remains unclear how the effect is modulated by disparate others and forms of uncertainty. In the current study, the authors address this question using an experimental paradigm from Suzuki et al. (2016). Participants changed their risk and ambiguity preferences towards others after observation. The social contagion effect was larger after observing risk-averse others (Experiment 1). On the other hand, no such asymmetric patterns were found in ambiguous decisions (Experiment 2).

Basically, this study is a replication and extension of Suzuki et al. (2016): You extended the findings of contagion effects to framing and decision-making under ambiguity. While I found it interesting, I have some comments on the analyses and discussion.

Major comments

- You have added the gain/loss framing to Suzuki et al. (2016), which is the contribution of this study. However, the new setting introduces the problem of loss aversion and reference point (aka status quo), doesn’t it? Whereas you analyzed the data as if the task were gain-only gambling like Suzuki et al. (2016), such data including loss domain are usually analyzed considering loss aversion (= the cumulative prospect theory). I would suggest reanalyzing the data (see the following paper for your reference).

- Sokol-Hessner et al. (2019) The Psychological and Neural Basis of Loss Aversion. https://journals.sagepub.com/doi/full/10.1177/0963721418806510

- Including this study and Suzuki et al. (2016), many neuroscience-based studies of social influence only look at one-way influence (influence from an observee to an observer). This is methodologically straightforward, but there is still a significant gap in understanding actual social behavior. It would be better to discuss the potential importance of looking at bidirectional social interaction processes as a future direction. For example, the following references are a few examples that took such a bidirectional approach.

- Mahmoodi et al. (2018). Reciprocity of social influence. https://www.nature.com/articles/s41467-018-04925-y

- Kuroda et al. (2022). Behavioral and neuro-cognitive bases for emergence of norms and socially shared realities via dynamic interaction. https://www.nature.com/articles/s42003-022-04329-1

- I found the 2nd paragraph in the discussion somewhat speculative (p. 16, line 427). For example, such an asymmetric effect was also found in the aforementioned study by Kuroda et al. (2022). They didn't use a gambling task but dot-estimation task. However, they found that participants had underestimation biases and that participants showed a stronger contagion when paired with a partner with an underestimation bias than a partner with an overestimation bias (Fig. S12 in Kuroda et al.). This pattern seems quite similar to the current study, doesn't it? I think we can understand these results as a process where people become more similar to others who are already somewhat similar to themselves (Fig. 1d). Or, you could discuss it in the context of literature on confirmation (e.g., refs. 13–17 you cited).

- I know Gilboa & Schmeidler's maximin expected utility model, but I'm curious about its prediction accuracy. How well did it reproduce participants' behavior? I'd like to see the prediction accuracy index or visualization, which is something like posterior predictive checking in the context of Bayesian parameter estimation.

- I don't usually comment on gender differences, but risky decision-making usually has a large gender difference. It's not necessary to do detailed additional analyses, but for more transparency (and given that you used only male observees), it would be a good idea to show gender differences in risk preference, ambiguity preference, and contagion effect at least.

Minor comments

- For clarification: In p.4, line 112, "… chose less risky gambles than those in the Loss frame" should be "… chose gambles less often than in the Loss Frame"? "less risky gambles" sounds not accurate.

- In p.20, line 546: You write "his/her choice" in the line, but the observees were both male. This might be a gender-sensitive expression, but it could be a little confusing for readers.

- In the Experiment 2, did you instruct participants that "you (participants) will experience the same gambles that observees experienced"? I.e., did you tell participants that the ground truth (the true probability) in the ambiguous gambles are same between participants and observees? Please clarify. This point may have affected their decisions.

- Most of the figures in the supplementary information are somewhat blurred to me. They are still readable but could be improved.

Reviewer #3 (Remarks to the Author):

In this manuscript, the authors examine how decision makers are influenced by observing simulated choices in the domain of risk and ambiguity. As a result, participants are influenced by the observed choices in both domains. In addition, there is asymmetry in that participants are more influenced by simulated risk-averse choices than risk-seeking choices in the domain of risk, while ambiguity-averse and ambiguity-seeking simulated choices affect participants equally in the domain of ambiguity.

In sum, I think this is an interesting research question, the authors present two original experiments, and overall, the manuscript is well-written. However, I have some comments concerning potential limitation of the design and the analyses, which I outline in the following.

1. Within-design of asking participants to predict the choices of "others" and making personal decisions: I think a disadvantage of the current design is that blocks with predictions of what the "other" chose and blocks with personal decisions are presented to participants alternatingly and in close proximity. Therefore, I think a psychologically not very interesting alternative explanation other than social contagion, could be that participants just confused the personal decision-making with the prediction task. Can the authors provide any evidence to alleviate this concern through, for example, attention checks asking about the current task, or a design in which predictions and personal decisions are separated more rigorously? I think, it is important to at least discuss this alternative explanation.
2. Deception of participants: Relatedly, participants were deceived in that the observed choices stem from a computer

simulation rather than a real human participant. Can the authors justify why this was necessary? In my view, such deception can compromise the quality of the research data if participants inform future participants about the deceptions (during one experiment, but also across experiments as a lab reputation). In principle, it seems to be possible to collect data from participants in this task and then select some of them that differ in risk taking to participants.
3. Using a stochastic power utility model for main inference: The stochastic power utility model is known to produce parameter correlations and can have poor estimation accuracy in some choice problem sets (Krefeld-Schwalb et al., 2022; Olschewski et al., 2022). Therefore, I think it is important to build trust in the reported model-based analyses. I would suggest to either also present and interpret the model-free analyses in the main task. Alternatively, the authors could perform a model recovery to estimate the measurement accuracy of the utility curvature parameter in their data set and discuss what this means for the interpretation of the results.
4. Exclusions of participants without pre-registration: I noticed that in both studies some participants were excluded based on choices or predictions. As these exclusions are based on dependent variables and were not pre-registered, I think it is important to examine the robustness of the reported findings by examining whether the results hold when including those participants.
5. Interpretation of difference between risk and ambiguity: The authors strongly interpret a difference in social contagion between risk and ambiguity. Namely, that participants show more contagion toward risk aversion than toward risk seeking, while no such asymmetry was observed in the case of ambiguity. However, risk and ambiguity were manipulated in separate experiments. Could the authors rule out that either the manipulation or the participant pool in Experiment 2 could have led to more measurement noise or that the effect size of social contagion in ambiguity is just lower than in risk, so that the authors had not enough power to detect the asymmetry for ambiguity. Potentially, Bayes factors could clarify the evidence there is against the asymmetry of social contagion in ambiguity. Alternatively, the authors could discuss the limitation of interpreting a null finding within one experiment as well as comparing between risk and ambiguity across experiments.
6. Presenting all created choice problems in a table: I could not fully understand the created choice problems, although they were explained in detail in the methods and in some additional figures. Maybe it would be easiest to add a table with all choice problems and their characteristics?

References

Krefeld-Schwalb, A., Pachur, T., & Scheibehenne, B. (2022). Structural parameter interdependencies in computational models of cognition. Psychological Review, 129(2), 313.

Olschewski, S., Sirotkin, P., & Rieskamp, J. (2022). Empirical underidentification in estimating random utility models: The role of choice sets and standardizations. British Journal of Mathematical and Statistical Psychology, 75(2), 252-292.

Note on appeals: In exceptional circumstances, it is in authors’ interest to appeal an editorial decision. More information on appeals is available here: https://www.nature.com/commspsychol/submit/editorial-process#appeals

Version 1:

Decision Letter:

** Please ensure you delete the link to your author homepage in this e-mail if you wish to forward it to your coauthors **

Dear Mr Pan,

Thank you for your patience during the peer-review process. Your manuscript titled "Asymmetric social influence on risk but not ambiguity preference in decision-making under uncertainty" has now been seen by 3 reviewers, and I include their comments at the end of this message. They find your work of interest but raised some important points. We are interested in the possibility of publishing your study in Communications Psychology, but would like to consider your responses to these concerns and assess a revised manuscript before we make a final decision on publication.

We therefore invite you to revise and resubmit your manuscript, along with a point-by-point response to the reviewers. Please highlight all changes in the manuscript text file.

Editorially, we consider conceptually, you need a more detailed explanation of the human choice data's recording and selection process, as well as a precise description of the trial-type judgment task's implementation. Methodologically, please provide a clearer report of the model-free analysis and a discussion on whether the asymmetric contagion effects are merely smaller, rather than absent, under ambiguity.

Please ensure you follow our statistical guidelines when reporting statistics (https://www.nature.com/commspsychol/submit/submission-guidelines#statistical-guidelines). Please note in particular our requirements for the reporting and interpretation of null-results. Non-significant findings derived from null-hypotheses

significance tests should be reported in full, but may not be interpreted. Where you interpret null results, this interpretation must be based on Bayes Factors or equivalence tests.

Our journal uses the convention of interpreting Bayes Factors > 0.33 & < 3 as inconclusive. Please do not interpret a null effect when the Bayes Factor is larger than 0.33.

As you revise the manuscript in response to these issues, please also implement all requests in the attached Mandatory Revision Requests document. All requirements listed in this document need to be fully met, or the work will be returned to you for further revisions without peer review. This workflow is in place to increase the likelihood that the paper will be accepted for publication. It reduces the number of rounds of revision (and review) and ensures that the reviewers vet a version of the article that is compliant with journal policies. If you have any questions regarding the required revisions, please contact the journal prior to resubmission to avoid a negative outcome.

We are committed to providing a fair and constructive peer-review process. Please don't hesitate to contact us if you wish to discuss the revision in more detail.

Please submit the following items:

- Revised manuscript
- Point-by-point response to the referees' comments
- Mandatory Revision Requests Table (attached).
- Cover letter (as a separate document)

via this link: Link Redacted .

** This url links to your confidential home page and associated information about manuscripts you may have submitted or are reviewing for us. If you wish to forward this email to co-authors, please delete the link to your homepage first **

We hope to receive your revised paper within 8 weeks; please let us know if you aren't able to submit it within this time so that we can discuss how best to proceed. If we don't hear from you, and the revision process takes significantly longer, we may close your file. In this event, we will still be happy to reconsider your paper at a later date, provided it still presents a significant contribution to the literature at that stage.

We look forward to seeing the revised manuscript and thank you for the opportunity to review your work.

Best regards,

Troby Lui, on behalf of

Yafeng Pan

Troby Lui, PhD
Associate Editor
Communications Psychology

Yafeng Pan, PhD
Editorial Board Member
Communications Psychology
orcid.org/0000-0002-5633-8313

REVIEWER EXPERTISE:
Reviewer #1: decision-making, computational modeling
Reviewer #2: decision-making, computational modeling
Reviewer #3: decision-making, computational modeling

REVIEWER REPORTS:

Reviewer #1 (Remarks to the Author):

Dear Authors,

Thank you for the excellent revision and for replicating the studies — great work. I am also very happy with your responses to my earlier questions.

I have only a few minor comments:

(1) I suggest removing the word “robust” from the abstract. The sentence “The results were fully replicated...” should be sufficient.

(2) I recommend replacing the words “conservative” and “adventurous” with “risk-averse” and “risk-seeking,” respectively, wherever possible.

(3) Page 6, line 4: Could you please clarify what is meant by “inverse temperature”?

(4) Figures 2f and 2i currently lack a legend. I assume the darker line represents risk-averse participants and the lighter line represents risk-seeking participants—please confirm or clarify.

(5) I appreciated your response to my question on reaction time (R1-4). If possible, I would suggest adding a few lines in the Discussion about how the observed patterns of reaction time could help explain the main results. As a reader I am puzzled by how the reaction time data correlates with asymmetry in risk contagion.

Reviewer #2 (Remarks to the Author):

The authors conducted a preregistered replication study, performed additional model checks and loss-aversion analysis, and expanded the Discussion to connect their findings to the broader literature on social influence and decision-making. Overall, the authors have fully addressed all of my previous concerns, and the revision has significantly strengthened the paper both methodologically and conceptually. I therefore support acceptance of the manuscript in its current form.

Reviewer #3 (Remarks to the Author):

I thank the authors for carefully taking all my comments into account and in particular for taking the effort to collect new data with improved versions of their experiments. I think the additional (replication) experiments greatly improved the empirical evidence for many of the authors’ claims. These additional experiments satisfactorily addressed my original comments 1,2, and 4. The only things that were missing for me in the report of these experiments were (sorry if they were there and I just did not find them): First, a detailed explanation of how the human choice data were recorded and selected, which were used to introduce social contagion in the replication studies. And second, an exact description of how the trial-type judgment task was implemented and how participants had to answered it (how was the question framed? Was it multiple-choice? If yes, what were the options?).

Concerning Comment 3, I greatly appreciate the extensive parameter recovery, which looks indeed very convincing. I was a bit surprised about this accuracy given the papers I cited in my previous review, which found much worse recoveries. But given that the recovery is so good, the inference is fully warranted. I still think that the model-free analysis should be more prominently featured in the main manuscript as a robustness, but I leave this to the authors. In either case, the current report of the model-free analysis is not fully clear to me. In particular, from the supplementary Figure S4, I did not fully understand which exact analysis was used to show that the asymmetric (Exp 1) or symmetric (Exp 2) contagion effects in the model-free analyses are similar to those reported for the model-based results.

Finally, I still struggle with my concerns expressed in my original Comment 5. The authors argue that they have evidence for the absents of asymmetry in social contagion in the domain of ambiguity. However, the Bayes Factor they report in the response letter is 0.55 (and is not reported in the manuscript as far as I can see), which speaks in favor of the null hypothesis, but is indecisive evidence according to standard Bayesian interpretations. Also, the point estimate for an asymmetric effect of social contagion in ambiguity in the pooled data was reported in the response letter as Cohen’s d of 0.256. This is small but not negligible and in the range of behavioral effects often found in the field of judgment and decision making. Additionally, although the authors replicated both risk and ambiguity contagion effects independently, they still did not examine both within one experiment. However, the comparison between experiments without a random allocation of participants to the domains is weak causal evidence for a difference in social contagion between both domains. In sum, in my view the authors should be more careful with their conclusions and at least discuss the possibility that the asymmetric contagion effects are just smaller instead of absent in ambiguity.

* TRANSPARENT PEER REVIEW: Communications Psychology uses a transparent peer review system. This means that we publish the editorial decision letters including Reviewers' comments to the authors and the author rebuttal letters online as a supplementary peer review file. However, on author request, confidential information and data can be removed from the published reviewer reports and rebuttal letters prior to publication. If your manuscript has been previously reviewed at another journal, those Reviewers' comments would not form part of the published peer review file.

** Visit Nature Research's author and referees' website at www.nature.com/authors for information about policies, services and author benefits**

Communications Psychology is committed to improving transparency in authorship. As part of our efforts in this direction, we are now requesting that all authors identified as ‘corresponding author’ create and link their Open Researcher and Contributor Identifier (ORCID) with their account on the Manuscript Tracking System prior to acceptance. ORCID helps the scientific community achieve unambiguous attribution of all scholarly contributions. You can create and link your ORCID from the home page of the Manuscript Tracking System by clicking on ‘Modify my Springer Nature account’ and following the instructions in the link below. Please also inform all co-authors that they can add their ORCIDs to their accounts and that they must do so prior to acceptance.
https://www.springernature.com/gp/researchers/orcid/orcid-for-nature-research

For more information please visit http://www.springernature.com/orcid

If you experience problems in linking your ORCID, please contact the Platform Support Helpdesk.

Version 2:

Decision Letter:

** Please ensure you delete the link to your author homepage in this e-mail if you wish to forward it to your coauthors **

Dear Mr Pan,

Your manuscript titled "Distinct patterns of social contagion under risk and ambiguity" has now been seen by our reviewers, whose comments appear below. In light of their advice I am delighted to say that we are happy, in principle, to publish a suitably revised version in Communications Psychology.

We therefore invite you to revise your paper one last time to address the remaining concerns of our reviewers and a list of editorial requests. At the same time we ask that you edit your manuscript to comply with our format requirements and to maximise the accessibility and therefore the impact of your work.

EDITORIAL REQUESTS:

Please review our specific editorial comments and requests regarding your manuscript in the attached "Editorial Requests Table". Please outline your response to each request in the right hand column. Please upload the completed table with your manuscript files as a Related Manuscript file.

If you have any questions or concerns about any of our requests, please do not hesitate to contact me.

SUBMISSION INFORMATION:

In order to accept your paper, we require the files listed here https://www.nature.com/documents/commsj-file-checklist.pdf .

OPEN ACCESS:

Communications Psychology is a fully open access journal. Articles are made freely accessible on publication. For further information about article processing charges, open access funding, and advice and support from Nature Research, please visit https://www.nature.com/commspsychol/open-access

At acceptance, you will be provided with instructions for completing the open access licence agreement on behalf of all authors. This grants us the necessary permissions to publish your paper. Additionally, you will be asked to declare that all required third party permissions have been obtained, and to provide billing information in order to pay the article-processing charge (APC).

* TRANSPARENT PEER REVIEW: Communications Psychology uses a transparent peer review system. On author request, confidential information and data can be removed from the published reviewer reports and rebuttal letters prior to publication. If you are concerned about the release of confidential data, please let us know specifically what information you would like to have removed. Please note that we cannot incorporate redactions for any other reasons.

* CODE AVAILABILITY: All Communications Psychology manuscripts must include a section titled "Code Availability" at the end of the methods section. We require that the custom analysis code supporting your conclusions is made available in a publicly accessible repository at this stage; please choose a repository that generates a digital object identifier (DOI) for the code; the link to the repository and the DOI must be included in the Code Availability statement. Publication as Supplementary Information will not suffice.

* DATA AVAILABILITY:
All Communications Psychology manuscripts must include a section titled "Data Availability" at the end of the Methods section. More information on this policy, is available in the Editorial Requests Table and at http://www.nature.com/authors/policies/data/data-availability-statements-data-citations.pdf.

Please use the following link to submit the above items:
Link Redacted
** This url links to your confidential home page and associated information about manuscripts you may have submitted or be reviewing for us. If you wish to forward this email to co-authors, please delete the link to your homepage first **

We hope to hear from you within two weeks; please let us know if you need more time.

Best regards,

Troby Lui

Troby Lui, PhD
Associate Editor
Communications Psychology

Yafeng Pan, PhD
Editorial Board Member
Communications Psychology
orcid.org/0000-0002-5633-8313

REVIEWERS' COMMENTS:

Reviewer #3 (Remarks to the Author):

The authors have done an excellent job clarifying my remaining open points and revising the manuscript so that the conclusions more accurately reflect the experimental evidence.

** Visit Nature Research's author and referees' website at www.nature.com/authors for information about policies, services and author benefits**

Open Access This Peer Review File is licensed under a Creative Commons Attribution 4.0 International License, which permits use, sharing, adaptation, distribution and reproduction in any medium or format, as long as you give appropriate credit to the original author(s) and the source, provide a link to the Creative Commons license, and indicate if changes were made.
In cases where reviewers are anonymous, credit should be given to 'Anonymous Referee' and the source.
The images or other third party material in this Peer Review File are included in the article's Creative Commons license, unless indicated otherwise in a credit line to the material. If material is not included in the article's Creative Commons license and your intended use is not permitted by statutory regulation or exceeds the permitted use, you will need to obtain permission directly from the copyright holder.
To view a copy of this license, visit https://creativecommons.org/licenses/by/4.0/

Reviewer #1:

Paper summary: This paper investigates the impact of social contagion on individuals' risk and ambiguity preferences. Specifically, it examines how a person's attitude toward risk (or ambiguity) is influenced by observing someone else choosing either a risk-averse (or ambiguity-averse) or risk-seeking (or ambiguity-seeking) option. The findings provide evidence of a social contagion effect: participants adjusted their risk attitudes in the direction of the observed behavior. Notably, the shift was stronger in the risk-averse direction than in the risk-seeking direction. In contrast, the contagion effect on ambiguity attitudes was symmetric, with participants equally influenced by both ambiguity-averse and ambiguity-seeking behaviors.

Reply: We thank the reviewer for the accurate summary of our work. Please see our replies to the following comments. Below, reviewer comments are in regular text; our replies are in bold; blue indicates the corresponding revisions in the main text and SI.

1. I can see the authors have done power calculations in the Appendix. But I still feel the sample size of the experiment is too small. It would really be great if authors are able to do a new study with a sufficiently larger sample size and replicate the findings.

Reply: We thank the reviewer for pointing out that the sample size in our study is small and suggesting a replication study with a larger sample size. This question is closely related to Question 5 by Reviewer 3, we thus combine these questions and address them below.

We agree with the reviewer that while the sample size in the original study meets the minimum requirement to detect a significant effect based on our power analysis, replication with a larger sample would strengthen the conclusions. Therefore, we conducted a preregistered replication study (see https://osf.io/f4wuy for the preregistration report) with a substantially larger sample size (total *N* = 123). The replication study confirmed our original findings, revealing an asymmetric social contagion effect in risk preference and a symmetric contagion effect in ambiguity preference. We have added these replication results to the updated manuscript (see updated Figs. 2g-i, 4g-i, and 5b–c).

In addition to the increase of sample size, the replication study also incorporated design modifications to address other potential limitations of the original study. Specifically, it (i) avoided the involvement of deception, (ii) clearly preregistered participant-exclusion criteria, and (iii) excluded the possibility that the observed contagion effect was driven by confusion between different trial types. Despite these modifications, the replication study fully replicated the main findings of the original study, providing strong evidence for the robustness of the observed asymmetric/symmetric contagion effect in risk/ambiguity preference.

We have revised the manuscript to present the replication results in the Main Text and to describe the updated design in the Methods.

Methods of the replication study:
Participants in the replication study

Experiment 1. The replication of Exp. 1 included 61 participants after excluding 8 who belonged to our pre-registered exclusion criteria (see “Pre-registered exclusion criteria for replication study”), which is the same as in the original experiment. 31 participants were randomly allocated to the Gain decision frame (15 female participants; age: 22.45 ± 2.46 y, mean ± SD), whereas the other 30 were in the Loss decision frame (14 female participants; age: 21.30 ± 2.56 y). We first calculated the effect size of the asymmetric risk contagion effect observed in the original Experiment 1: Cohen’s d = 0.9031. Based on this, we then used G*Power to estimate that the minimum number of participants required per condition is 10 in total, assuming a statistical power of 0.8 and α = 0.05. Although this analysis suggests a relatively small sample size, we planned to collect a larger sample due to concerns over replication reliability in studies with small sample sizes. Specifically, we aimed to include at least 30 valid participants per condition after applying the exclusion criteria. The sample size for the Replication of Experiment 1 is pre-registered (https://osf.io/f4wuy).

Experiment 2. The replication of Exp. 2 included 62 participants after excluding 7 who belonged to our pre-registered exclusion criteria (see “Pre-registered exclusion criteria for replication study”), which is the same as in the original experiment. 32 participants were randomly allocated to the Gain decision frame (15 female participants; age: 21.84 ± 3.28 y, mean ± SD), whereas the other 30 were in the Loss decision frame (16 female participants; age: 22.40 ± 2.22 y). In the original Experiment 2, we found evidence for a symmetric ambiguity contagion effect, with participants adjusting their ambiguity preferences similarly toward both ambiguity-seeking and ambiguity-averse observees. To ensure that the absence of a statistically significant asymmetry was not due to insufficient statistical power, we calculated the effect size of the asymmetric ambiguity contagion effect (if it exists) observed in the original Experiment 2 (effect size: Cohen’s d = 0.2278). A sensitivity analysis (G*Power, two-tailed t-test, α = 0.05, power = 0.8) revealed that detecting an effect of this small magnitude would require approximately 154 participants per condition—a sample size beyond practical feasibility. To further confirm this absence of symmetric effect, we plan to recruit at least 30 valid participants per condition in the Replication of Experiment 2, after exclusions. Importantly, this sample size ensures sensitivity to asymmetry comparable in magnitude to the asymmetric risk contagion effect observed in Experiment 1. By recruiting this sample, we aimed to replicate the symmetric ambiguity contagion effect while rigorously testing the robustness of the null asymmetry finding. The sample size for the Replication of Experiment 2 is pre-registered (https://osf.io/f4wuy).

Experimental task in the replication study

Pre-registered exclusion criteria for replication study. Participants were excluded from the whole data analyses if they meet any of the following: (1) low prediction accuracy in Predict trials after the observation in at least one session (threshold = 50% chance level), (2) extremely biased or invariant choices, indicating a lack of meaningful engagement (e.g., always choosing gamble or always choosing sure option), (3) explicit disbelief in the experimental setting or suspicion about the manipulation, as revealed during debriefing, and (4) fail to pass the attention check (i.e., catch trials). Moreover, for the calculation of the contagion ratio (defined as the contagion effect divided by the preference distance between the observer and the observee), participants with zero or negative distance toward the observee (same preference as the observee or even more extreme) were excluded from the analysis. All exclusion criteria are pre-registered (https://osf.io/f4wuy).

Modifications in the replication study. The design of the replication study remained the same as the original study except for the following three modifications. First, to enhance ecological validity and avoid potential demand effects, we used recorded behavior from two actual human participants instead of using

simulated observees as in the original study. To be consistent with the original study as much as possible, the behavioral patterns of the chosen observees in the replication study closely matched those simulated observees used in the original study. Second, to protect the observees' privacy, we replaced the original back-view photographs with cartoon icons representing the observees. These cartoon photos can also mitigate the influence of observees' gender on the contagion effect. Third, we included a new trial-type judgment task at the end of each mini-block to help rule out the possibility that the observed behavioral changes are due to participants' confusion between Self and Prediction trials, rather than genuine social contagion effects. All these modifications were pre-registered (https://osf.io/f4wuy).

Data analyses in the replication study

The same data analysis methods were used in the replication study as in the original study, except otherwise specified.

Replication Results:

Replication of asymmetry in risk contagion. We replicated the asymmetric risk contagion effect with another independent sample of participants in a preregistered study (N = 61, see Methods for details). ANOVA analysis on contagion ratio with the replication sample again revealed a consistent asymmetric risk contagion effect in both the gain and loss frames: subjects were more aligned with risk-averse observees than with risk-seeking ones ($F(1,59) = 26.408$, $p < 0.001$, Fig. 2g). Neither the main effect of the decision frame ($F(1,59) = 0.000$, $p = 0.983$) nor the frame × preference interaction effect ($F(1,59) = 0.048$, $p = 0.828$) was statistically significant. In line with the contagion ratio analysis, linear regression analyses showed the regression slope for the risk-averse observee was also significantly steeper than that for the risk-seeking observee (regression coefficients: $coef_{\text{risk-averse}} = 0.426 \pm 0.070$, $p < 0.001$; $coef_{\text{risk-seeking}} = 0.118 \pm 0.105$, $p = 0.226$; $coef_{\text{risk-averse}} - coef_{\text{risk-seeking}} = 0.308 \pm 0.124$, $p = 0.015$; Fig. 2h). We also replicated the asymmetric effects found in RT results. Participants who observed a risk-seeking observee responded faster as the observer-observee risk preference distance increased, but not after the risk-averse observations ($coef_{\text{risk-seeking}} = -0.629 \pm 0.161$, $p < 0.001$; $coef_{\text{risk-averse}} = 0.183 \pm 0.169$, $p = 0.283$; $coef_{\text{risk-averse}} - coef_{\text{risk-seeking}} = 0.813 \pm 0.237$, $p < 0.001$; Fig. 2i). These replication results confirmed the robustness of the asymmetric risk contagion effect observed in the original experiment.

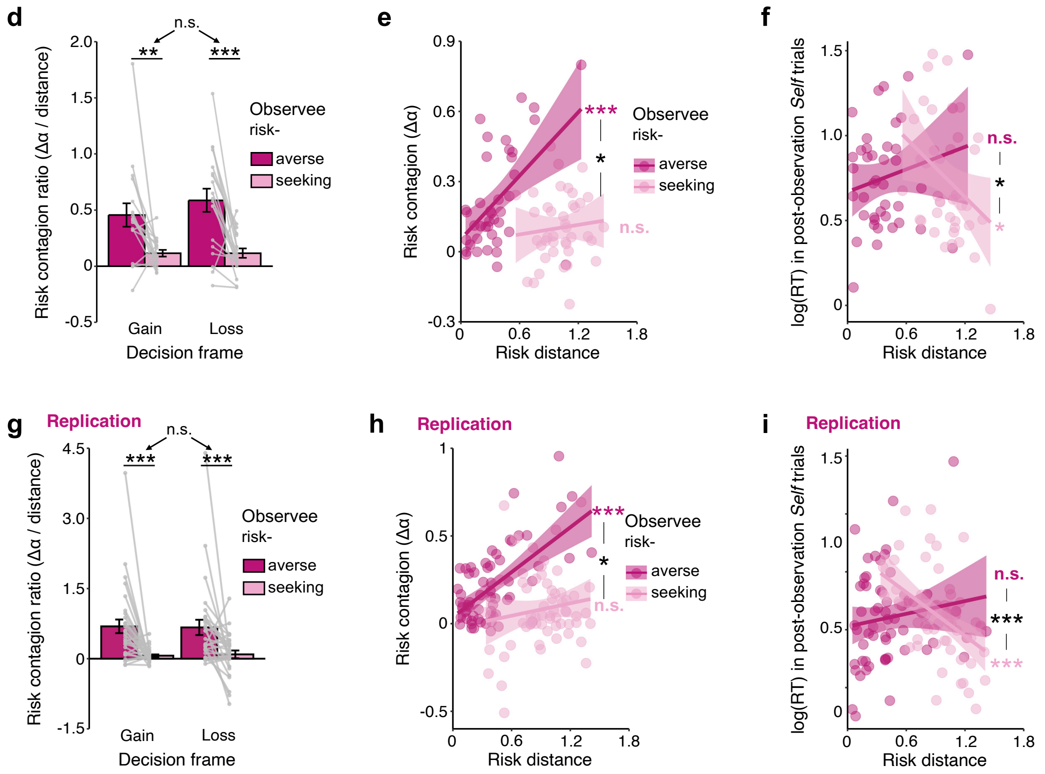

Fig. 2. Risk contagion effect. (**d**) Risk contagion ratio (defined as the ratio of risk contagion to risk distance) is plotted as a function of the decision frame and observees' risk preference. Gray dots linked by a line represent the risk contagion ratio of each participant when observing risk-averse (magenta) and risk-seeking (pink) observees. Error bars represent the SEM. (**e**) Risk contagion as a function of risk distance. (**f**) The log-transformed reaction time in post-observation Self trials, as a function of risk distance. In (**e**) and (**f**), each magenta/pink point represents a participant when observing risk-averse/risk-seeking observees. Fitted regression lines are plotted, and the shaded area around the fitted line reflects 95% confidence intervals (CIs). (**g-i**) The replication results for panels (**d**), (**e**), and (**f**), respectively, derived from the replication sample. ***$p < 0.001$; **$p < 0.01$; *$p < 0.05$; n.s., not significant.

Replication of symmetry in ambiguity contagion. We replicated the symmetric ambiguity contagion effect with another independent sample of participants in a preregistered study ($N = 62$, see Methods for details). ANOVA analysis on contagion ratio with the replication sample again revealed a consistent symmetric ambiguity contagion effect in both the gain and loss frames: neither the observee's ambiguity preference ($F(1,60) = 1.699$, $p = 0.197$), the decision frame ($F(1,60) = 1.142$, $p = 0.290$), nor the interaction of these two variables affected the ambiguity contagion ratio ($F(1,60) = 0.244$, $p = 0.623$, Fig. 4g), suggesting a consistent influence of observees' ambiguity preferences on participants' ambiguity contagion regardless of the decision frames and the observees' ambiguity preferences. In line with the contagion ratio analysis, linear regression analyses showed the ambiguity contagion effect was positively correlated with ambiguity distance, no matter when the observee was ambiguity-averse or ambiguity-seeking, showing no asymmetry ($coef_{\text{ambiguity-averse}} = 0.678 \pm 0.156$, $p < 0.001$; $coef_{\text{ambiguity-seeking}} = 0.469 \pm 0.106$, $p < 0.001$; $coef_{\text{ambiguity-averse}} - coef_{\text{ambguity-seeking}} = 0.209 \pm 0.186$, $p = 0.263$; Fig. 4h). We also replicated the symmetry in RT and found that RT did not change with the ambiguity distance when the observees was either ambiguity-averse or ambiguity-seeking ($coef_{\text{ambiguity-averse}} = 0.254 \pm 0.147$, $p = 0.084$; $coef_{\text{ambiguity-seeking}} = 0.192 \pm 0.129$, $p = 0.141$; $coef_{\text{ambiguity-averse}} - coef_{\text{ambguity-seeking}} = 0.062 \pm 0.199$, $p = 0.756$;

Fig. 4i). These replication results confirmed the robustness of the symmetric ambiguity contagion effect discovered in the original study.

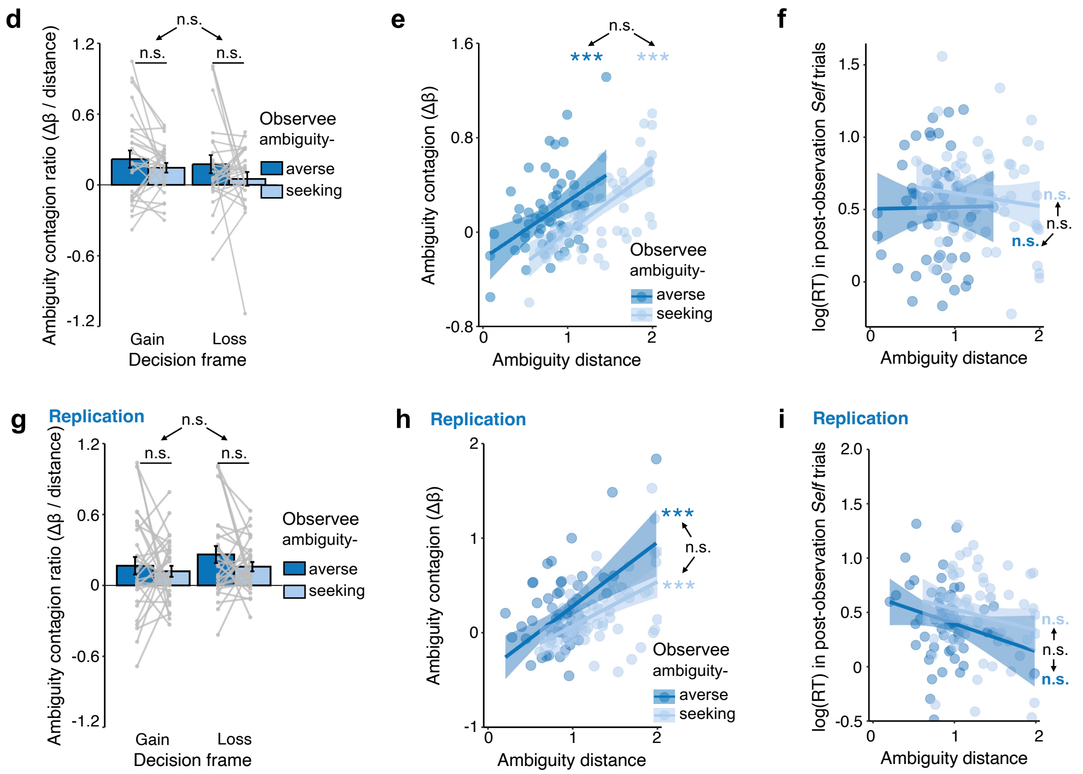

Fig. 4. Ambiguity contagion effect. (**d**) Degree of ambiguity contagion ratio as a function of the decision frame and the observee's ambiguity-preference type (mean ± SEM). Gray dots linked by a line represent the ambiguity contagion ratio of each participant when observing ambiguity-averse and ambiguity-seeking observees. (**e**) Ambiguity contagion as a function of ambiguity distance. (**f**) The log-transformed reaction time in post-observation Self trials, as a function of ambiguity distance. In (**e**) and (**f**), each blue/light-blue point represents a participant when observing the ambiguity-averse/ambiguity-seeking observee, respectively. Fitted regression lines are plotted, and the shaded area around the fitted line reflects 95% CIs. (**g-i**) The replication results for panels (**d**), (**e**), and (**f**), respectively, derived from the replication sample. ***$p < 0.001$; **$p < 0.01$; *$p < 0.05$; n.s., not significant.

Comparing contagion effects of risk and ambiguity preferences across replication experiments. The same analysis on our replication sample (N = 123) generated consistent results of asymmetric risk contagion and symmetric ambiguity contagion effects (uncertainty context [risk > ambiguity]: $F(1,121) = 9.715$, $p = 0.002$); observee type [averse > seeking]: $F(1,121) = 27.12$, $p < 0.001$; interaction: $F(1,121) = 16.80$, $p < 0.001$; post hoc t-test in risk context: $t_{averse>seeking}(121) = 6.577$, $p < 0.001$; post hoc t-test in ambiguity context: $t_{averse>seeking}(121) = 0.810$, $p = 0.419$; Fig. 5b). After pooling the data from both original and replication experiments (N = 219), the results remained the same (uncertainty context [risk > ambiguity]: $F(1,217) = 18.38$, $p < 0.001$; observee type [averse > seeking]: $F(1,217) = 47.41$, $p < 0.001$; interaction: $F(1,217) = 26.43$, $p < 0.001$; post hoc t-test in risk context: $t_{averse>seeking}(217) = 8.464$, $p < 0.001$; post hoc t-test in ambiguity context: $t_{averse>seeking}(217) = 1.486$, $p = 0.139$; Fig. 5c), demonstrating the robustness of the results.

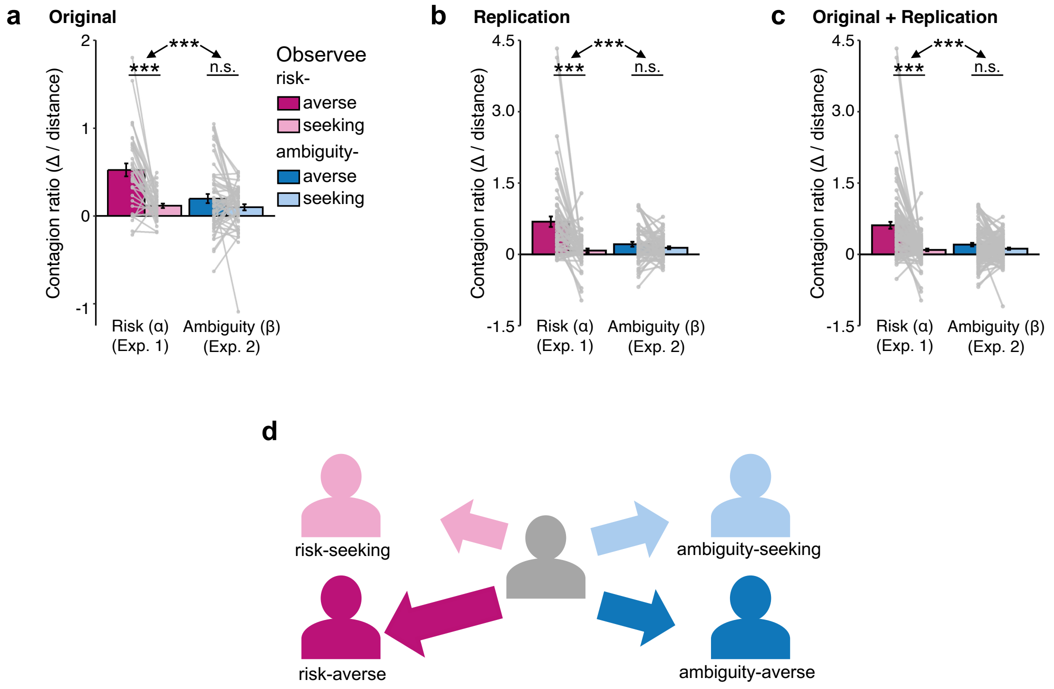

Fig. 5. Asymmetric risk and symmetric ambiguity contagion effects across two experiments. (**a**) Model-based contagion ratio of risk preference (*α* in Exp. 1) and ambiguity-preference (*β* in Exp. 2) as a function of the observees' risk/ambiguity preferences (mean ± SEM). Gray dots linked by a line represent the risk/ambiguity contagion of each participant when observing risk-averse/seeking and ambiguity-averse/seeking observees. The uppermost asterisk indicates that the interaction effect between uncertainty type (risk/ambiguity) and observees' uncertainty preference is significant. (**b**) The replication results from the replication sample. (**c**) The results after pooling the data from both original and replication studies. (**d**) Summary illustration of an asymmetric risk contagion effect (stronger risk-averse contagion than risk-seeking contagion) and symmetric ambiguity contagion effect (comparable ambiguity-averse and ambiguity-seeking contagion). ***$p < 0.001$; **$p < 0.01$; *$p < 0.05$; n.s., not significant.

2. I can see that the subjects go through many trials in both the experiments. Why then collapse the data and do an ANOVA. Wouldn't a panel data analysis that treats each data point independently be better?

   Reply: We appreciate the suggestion. The primary aim of the current work was to examine changes in latent uncertainty preference after observing others' decisions. Critically, estimating a person's risk or ambiguity preference requires model fitting using data aggregated across multiple trials; single decisions are noisy and influenced by multiple factors (e.g. risk preference, ambiguity preference, and random choice variability). If we treat each trial as an independent data point in a regression analysis, we cannot attribute any observed changes specifically to shifts in participants' uncertainty preferences. Our model-based approach, utilizing standard power-utility/maxmin models, aggregates across trials to extract stable, interpretable parameters of risk and ambiguity preferences. As described in the Main Text and Methods for both experiments, this approach enables rigorous tests of whether these latent preferences shift after observing others' decisions.

For example, in the ambiguity condition, participants' choices depend on at least three parameters: risk preference, ambiguity preference, and choice stochasticity. These individual parameters, however, could be differentiated using a formal model-fitting process. Our analytic approach strictly follows that of prior studies using similar experimental paradigms (Suzuki et al. 2016). In addition, we also present model-free analyses in the Supplementary Information (Fig. S4 and Fig. S7), focusing on the proportion of risky or ambiguous choices. These model-free results are highly correlated with the model-based parameter estimates, suggesting that our model-based approach indeed captured the psychological processes in social contagion.

Per the reviewer's suggestion, we also conducted panel-style trial-level analyses as a robustness check for Experiment 1 in which choices of risky gambles primarily index risk preference. For the reasons outlined above, we did not apply the same approach to Experiment 2 since trial-level choices of the ambiguous option jointly reflect risk and ambiguity preferences, making changes in ambiguity preference unidentifiable without model-based estimation. We fit logistic mixed-effects models to the raw choices (choice of gamble ~ gamble value + probability + sure value + post vs. pre; random intercepts by subject). For clarity, we first estimated separate models within each of the four cells (Decision frame × Observee type). As expected, the post–pre coefficient is negative after observing a risk-averse observee and positive after observing a risk-seeking observee (Table a-d). These results correspond to the four bars in Fig. 2b, which are all significantly higher than zero, showing a contagion effect towards risk-averse and risk-seeking observees in both the gain and loss frames. Moreover, consistent with the main results, the degree of the post–pre shift is larger after observing a risk-averse than a risk-seeking observee, showing a tendency of the asymmetric risk-contagion effect.

Table a. **Gain frame | risk-averse** observee

Independent Variables and Covariates	*β*	*SE*	*p*-value
(Intercept)	-0.217	0.082	0.017*
Value of the gamble option (the initial endowment)	0.027	0.003	<0.001***
Probability of the gamble option	1.137	0.062	<0.001***
Value of the sure option	-0.046	0.003	<0.001***
**Post-observation – pre-observation**	**-0.091**	**0.023**	**<0.001*** **

Table b. **Gain frame | risk-seeking** observee

Independent Variables and Covariates	*β*	*SE*	*p*-value
(Intercept)	-0.287	0.062	<0.001***
Value of the gamble option (the initial endowment)	0.026	0.002	<0.001***
Probability of the gamble option	1.156	0.073	<0.001***
Value of the sure option	-0.044	0.004	<0.001***
**Post-observation – pre-observation**	**0.055**	**0.019**	**0.005** **

Table c. **Loss frame | risk-averse** observee

Independent Variables and Covariates	*β*	*SE*	*p*-value
(Intercept)	-0.167	0.075	0.037*
Value of the gamble option (the initial endowment)	0.022	0.002	<0.001***
Probability of the gamble option	1.247	0.067	<0.001***
Value of the sure option	-0.037	0.004	<0.001***
**Post-observation – pre-observation**	**-0.151**	**0.025**	**<0.001*** **

Table d. **Loss frame | risk-seeking** observee

Independent Variables and Covariates	*β*	*SE*	*p*-value
(Intercept)	-0.279	0.067	<0.001***
Value of the gamble option (the initial endowment)	0.023	0.003	<0.001***
Probability of the gamble option	1.224	0.070	<0.001***
Value of the sure option	-0.040	0.004	<0.001***
**Post-observation – pre-observation**	**0.056**	**0.022**	**0.018***

To directly compare the size of contagion after observing risk-averse versus risk-seeking observees within a single model, we pooled all conditions and re-coded sessions to represent absolute preference change. For risk-seeking observees, the pre-observation session was coded as low (0) and the post-observation session as high (1); for risk-averse observees, the coding was reversed (pre = high [1], post = low [0]). This alignment puts all changes on the same "absolute" scale. We then tested the interaction between Absolute preference change (high–low) and Observee type. The interaction was significant, indicating larger absolute shifts after observing risk-averse than risk-seeking observees (risk-averse – risk-seeking: β = 0.067 ± 0.022, p = 0.003; Table e), consistent with the results from our ANOVA analysis in Fig. 2b.

Table e. All conditions | comparison of contagion magnitudes between observe type

Independent Variables and Covariates	*β*	*SE*	*p*-value
(Intercept)	-0.413	0.058	<0.001***
Value of the gamble option (the initial endowment)	0.024	0.002	<0.001***
Probability of the gamble option	1.193	0.042	<0.001***
Value of the sure option	-0.042	0.002	<0.001***
Decision frame	0.083	0.024	<0.001***
Absolute preference change (high–low)	0.055	0.014	<0.001***
Observee type	-0.024	0.016	0.152
**Absolute preference change × Observee type**	**0.067**	**0.022**	**0.003****

In summary, panel data analyses are valuable robustness checks (and they reproduce the key effects), but our primary question concerns shifts in latent preferences, which are most cleanly identified by session-level model fitting followed by group-level tests. Accordingly, our primary analyses rely on model-derived parameters and group-level tests (ANOVA), with the panel regressions reported as complementary robustness checks.

- **Suzuki, S., Jensen, E. L. S., Bossaerts, P. & O'Doherty, J. P. Behavioral contagion during learning about another agent's risk-preferences acts on the neural representation of decision-risk. *Proc Natl Acad Sci U S A* 113, 3755–3760 (2016).**

3. Is the asymmetric social contagion effect towards risk aversion due to the fact that risk seeking choices of the observee happen in the earlier block (block 2) while risk averse choices of the observee happen in the later block *block 4)? Could this also be explained by the reaction time data?

Reply: We thank the reviewer for raising this question about the potential influence of observee order. In our design, the order of observees counterbalanced across participants so it's unlikely that the asymmetric social congation effect stems from session orders. Specifically, half of the participants observed a risk-seeking observee in Session 2 and a risk-averse observee in Session 4, while the other half observed a risk-averse observee in Session 2 and risk-seeking observee in Session 4. The original wording—"The session order of these two observees (Sessions 2 and 4) was counterbalanced across participants" in the Main Text and "Observees' order

counterbalanced" in Fig. 1a—might not have highlighted this fact sufficiently. In the revised Main Text, we now add a detailed explanation that the order of different observees appearing was counterbalanced across participants, now reading:

"The session order of these two observees (Sessions 2 and 4) was counterbalanced across participants. Specifically, half of the participants observed a risk-seeking observee in block 2 and a risk-averse observee in block 4, while the other half observed a risk-averse observee in block 2 and risk-seeking observee in block 4."

To further rule out the order effect, we split the data according to the observee order. Both groups showed the same asymmetric contagion effect: participants who observed the risk-averse observee first (Session 2) still showed a significant asymmetry ($F(1,17) = 8.948$, $p = 0.008$), as did participants who observed the risk-seeking observee first ($F(1,16) = 23.555$, $p < 0.001$; see the figure below). These results allow us to rule out the potential confound of observee order on the contagion effects we observed.

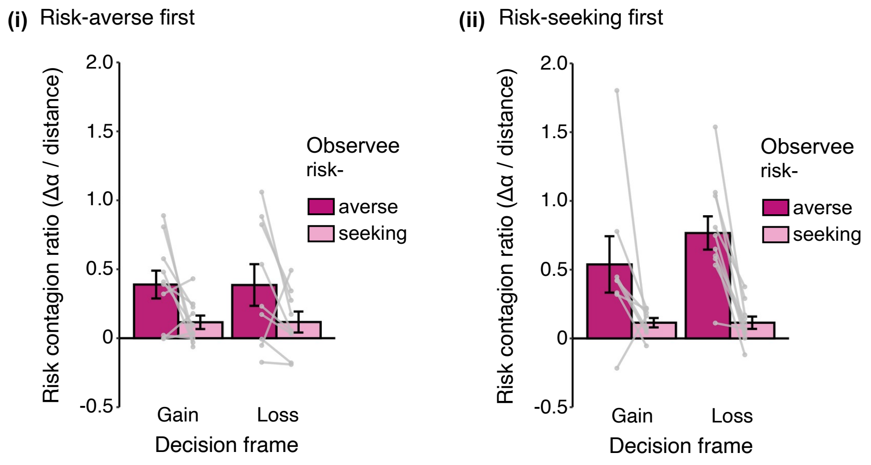

4. What does the reaction time data tell about the mechanism?

Reply: We thank the reviewer for this insightful question, which prompts us to elaborate on the potential mechanism reflected in the reaction time (RT) data. In our view, RT and choice capture complementary aspects of the same underlying decision process, so we examined whether the choice-contagion patterns are mirrored in RT. To explore this possibility, we conducted an exploratory RT analysis to examine whether the effect of uncertainty distance on RT is asymmetric or symmetric in the risk versus ambiguity contexts. Consistent with the choice data, we found an asymmetric relationship between RT and uncertainty distance in the risk context, but not in the ambiguity context (Fig. 2f,i & 4f,i).

A parsimonious account is that RT indexes the time needed to integrate others' choices with one's own preferences—longer RTs reflect greater deliberation, whereas shorter RTs reflect rapid disengagement or rejection (Gold & Shadlen, 2007; Ratcliff et al., 2016; Toelch & Dolan, 2015; Germar et al., 2016). Greater distance from a risk-seeking observee appears to prompt quicker rejection of the social influence, resulting in faster responses as distance increases. In contrast, greater distance from a risk-averse observee is not related to faster RT, suggesting continued

consideration/adoption of cautious strategies. Thus, the RT pattern mirrors the choice results, suggesting that the asymmetry in risk contagion might be driven by differences in cognitive conflict or effort when adopting versus rejecting an observed strategy. Consistent with this interpretation, RTs do not covary with ambiguity-preference distance—indicating comparable integration of influence from ambiguity-seeking and ambiguity-averse observees and aligning well with the symmetric pattern in the ambiguity domain.

However, it should also be noted that the current task is not specifically designed to tackle the RT related mechanisms. Therefore, we here only make some speculations about the mechanisms reflected by the RT based on the current findings. Definitive adjudication between process accounts will require future work using RT-optimized designs with formal process models. Nonetheless, the current RT pattern provides convergent behavioral evidence for the proposed mechanism behind the contagion effects.

- **Gold, J. I., & Shadlen, M. N. (2007). The neural basis of decision making. *Annu. Rev. Neurosci.*, 30(1), 535-574.**
- **Ratcliff, R., Smith, P. L., Brown, S. D., & McKoon, G. (2016). Diffusion decision model: Current issues and history. *Trends in cognitive sciences, 20*(4), 260-281.**
- **Toelch, U., & Dolan, R. J. (2015). Informational and normative influences in conformity from a neurocomputational perspective. *Trends in cognitive sciences*, *19*(10), 579-589.**
- **Germar, M., Albrecht, T., Voss, A., & Mojzisch, A. (2016). Social conformity is due to biased stimulus processing: Electrophysiological and diffusion analyses. *Social Cognitive and Affective Neuroscience*, *11*(9), 1449-1459.**

5. There is deception involved in the experiment where the authors tell the subjects that they observe the choice of a real subject, when they don't. I hope the subjects were debriefed about this at the end of the experiment. Could this be done in real by providing an actual choice of a risk averse subject ? See for example Narayanan et al. (2024), which has also found that decision makers tend to anchor on algorithmic advice that contrast with their own risk attitudes.

Reply: We thank the reviewer for raising this important point and for providing relevant references. The design of our original study followed previous work, including the use of simulated observee choices. All participants were fully debriefed at the end of the experiment.

We agree that avoiding deception is preferable whenever possible. In a preregistered replication study, we replaced simulated observee choices with recorded choices from real human participants and replicated the main findings of the original study. This confirms that the observed social contagion effects are not dependent on the use of simulated choices.

Modifications in replication study. The design of the replication study remained the same as the original study except for the following three modifications. First, to enhance ecological validity and avoid potential demand effects, we used recorded behavior from two actual human participants instead of using simulated observees as in the original study. To be consistent with the original study as much as possible, the behavioral patterns of the chosen observees in the replication study closely matched those simulated observees used in the original study. Second, to protect the observees' privacy, we replaced the original back-view photographs with cartoon icons representing the observees. These

cartoon photos can also mitigate the influence of observees' gender on the contagion effect. Third, we included a new trial-type judgment task at the end of each mini-block to help rule out the possibility that the observed behavioral changes are due to participants' confusion between Self and Prediction trials, rather than genuine social contagion effects. All these modifications were pre-registered (https://osf.io/f4wuy).

Furthermore, Suzuki et al. (2016) found that the risk contagion effect does not differ when participants observe a human versus a computer agent, a result consistent with our findings and those of Narayanan et al. (2025), who reported that risk-averse recommendations have similar effects regardless of whether the advice comes from a human or an algorithm. We have incorporated these points into the Discussion to provide additional context and strengthen the interpretation of our results:

"Furthermore, a preregistered replication study using recorded choices from real participants yielded similar effects, consistent with previous studies that suggest social contagion operates similarly regardless of whether the source is a human or an algorithm[5,49]."

References:

5. Suzuki, S., Jensen, E. L. S., Bossaerts, P. & O'Doherty, J. P. Behavioral contagion during learning about another agent's risk-preferences acts on the neural representation of decision-risk. *Proc Natl Acad Sci U S A* 113, 3755–3760 (2016).
49. Narayanan, P., Somasundaram, J. & Seifert, M. Risk-averse algorithmic support and inventory management. *Eur J Oper Res* 322, 993–1004 (2025).

6. I am surprised that the asymmetric contagion effect of risk attitudes do not happen in the second experiment when the risk attitudes are disintegrated out in the model. What is the reason?

   Reply: Thank you for raising this point. For clarity, Experiment 2 also shows an asymmetric risk-contagion effect. Because Experiment 2 emphasized ambiguity contagion, the risk contagion effect was reported in the Supplementary Information (Fig. S8). We have revised the Results to highlight this finding more explicitly and to cite the SI directly.

   "Besides, despite not being the focus of Exp. 2, we still replicated a similar pattern of asymmetric risk contagion, with a smaller but significant effect (Fig. S8c), where participants showed a stronger contagion ratio when observing risk-averse observees than risk-seeking ones. Although risk preferences changed little here, linear regressions still showed that risk distance exerted a more significant influence on risk-averse than risk-seeking contagion (Fig. S8b), consistent with Exp. 1. Together, these findings showed that participants' risk and ambiguity preferences change independently, implying that they might be associated with distinct psychological bases. Moreover, such intrinsic differences could be explicitly revealed and amplified by observing others' decisions in similar decision contexts."

7. In Figure 4d, directionally the effects are very similar to that of the risk case. They are not significant though. Would it be that with higher sample size there could also be an asymmetric social contagion towards ambiguity aversion similar to the risk averse case? This is the reason why an experiment with larger sample size would be useful to test the robustness of the findings.

Reply: We thank the reviewer for pointing out the directional similarity in risk and ambiguity contagion and for suggesting a replication study with a larger sample size. Following the reviewer's suggestion, we conducted a preregistered replication study with 62 additional participants in the ambiguity decision context. The results replicated our original findings in both the contagion ratio and regression analyses (Fig. 4g-h). We again found no significant asymmetric social contagion toward ambiguity aversion, demonstrating the robustness of our original results. Here are the replication results we added to the Main Text (also see the reply to Question 1 of Reviewer 1):

Replication of symmetry in ambiguity contagion. We replicated the symmetric ambiguity contagion effect with another independent sample of participants (N = 62, see Methods for details). ANOVA analysis on contagion ratio with the replication sample again revealed a consistent symmetric ambiguity contagion effect in both the gain and loss frames: neither the observee's ambiguity preference ($F(1,60) = 1.699$, $p = 0.197$), the decision frame ($F(1,60) = 1.142$, $p = 0.290$), nor the interaction of these two variables affected the ambiguity contagion ratio ($F(1,60) = 0.244$, $p = 0.623$, Fig. 4g), suggesting a consistent influence of observees' ambiguity preferences on participants' ambiguity contagion regardless of the decision frames and the observees' ambiguity preferences. In line with the contagion ratio analysis, linear regression analyses showed the ambiguity contagion effect was positively correlated with ambiguity distance, no matter when the observee was ambiguity-averse or ambiguity-seeking, showing no asymmetry ($coef_{\text{ambiguity-averse}} = 0.678 \pm 0.156$, $p < 0.001$; $coef_{\text{ambiguity-seeking}} = 0.469 \pm 0.106$, $p < 0.001$; $coef_{\text{ambiguity-averse}} - coef_{\text{ambguity-seeking}} = 0.209 \pm 0.186$, $p = 0.263$; Fig. 4h).

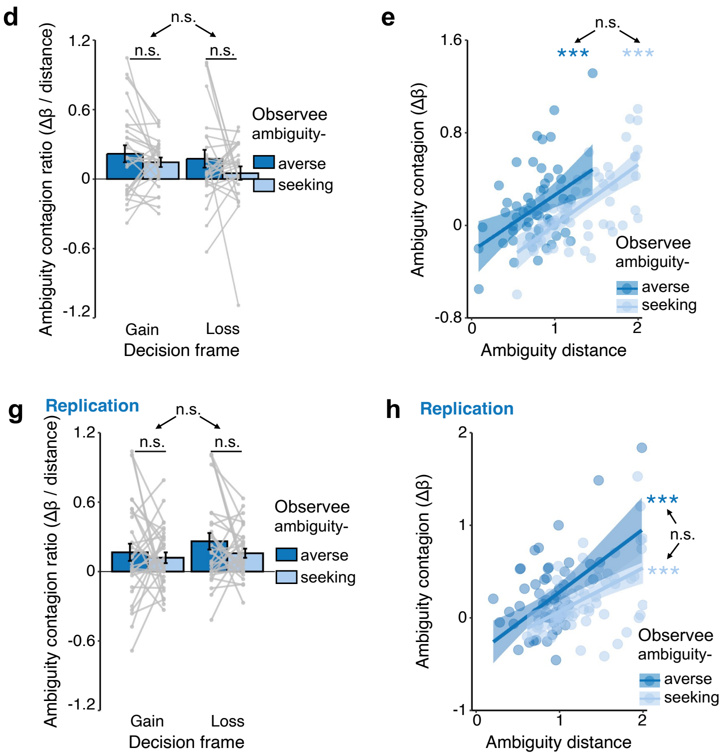

Importantly, when pooling the data from the original and replication studies (*N* = 118), ambiguity contagion remained symmetric (Fig. 5c). Together, these findings indicate that the absence of

asymmetry in ambiguity contagion is robust and replicable (effect size: Cohen's d = 0.256; also see the reply to Question 1 of Reviewer 1):

After pooling the data from both original and replication experiments ($N = 219$), the results remained the same (uncertainty context [risk > ambiguity]: $F(1,217) = 18.38$, $p < 0.001$; observee type [averse > seeking]: $F(1,217) = 47.41$, $p < 0.001$; interaction: $F(1,217) = 26.43$, $p < 0.001$; post hoc t-test in risk context: $t_{averse>seeking}(217) = 8.464$, $p < 0.001$; post hoc t-test in ambiguity context: $t_{averse>seeking}(217) = 1.486$, $p = 0.139$; Fig. 5c), demonstrating the robustness of the results.

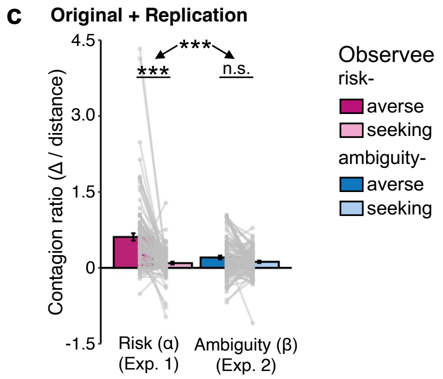

Moreover, the focus of our study is the difference between risk and ambiguity contagion, i.e., the interaction between uncertainty type (risk vs. ambiguity) and the observee's preference (seeking vs. averse). When comparing across two experiments, we found that risk contagion shows a significantly stronger asymmetry than ambiguity contagion, as reflected in the significant interaction effect (Fig. 5).

Additionally, when we examine the results of the regression analyses, this asymmetric trend in ambiguity contagion is not evident (see Fig. 4e; see also Fig. 2e for the corresponding risk contagion results). Specifically, regardless of whether the observee is ambiguity-seeking or ambiguity-averse, ambiguity contagion was significantly correlated with ambiguity distance, with comparable regression coefficients ($coef_{ambiguity\text{-}averse} = 0.486 \pm 0.135$, $p < 0.001$; $coef_{ambiguity\text{-}seeking} = 0.522 \pm 0.082$, $p < 0.001$).

In summary, the replication confirms that ambiguity contagion remains symmetric, whereas the asymmetry is specific to risk contagion; increasing sample size does not change this conclusion.

8. In Figure 3b, why is the correct prediction of risk behavior lower for the middle and end trials, when it should typically be the opposite?

   Reply: We thank the reviewer for raising this point. In Experiment 2, the "Start" predictions occur immediately after participants observed only risky choices, so risk predictions at "Start" reflect pure learning of the observee's risk preference and are expected to be near ceiling (Fig. 3b). By contrast, the "Middle"/"End" predictions follow blocks in which participants also observed ambiguity trials. By design, the observees' risk and ambiguity preferences were opposed (ambiguity-averse observee slightly risk-seeking; ambiguity-seeking observee slightly risk-

averse). This design aimed to help participants differentiate between the two preferences. However, because risk preference was not the primary focus of Experiment 2, the observees' risk tendencies were intentionally subtle—slightly risk-seeking or slightly risk-averse—making it hard for participants to identify. Therefore, when participants first encounter the observee's opposed risk vs. ambiguity preferences in the "Middle"/"End" block, this new ambiguity information inevitably influences risk-prediction accuracy thereafter, making the Start–Middle–End trajectory for risk predictions not interpretable as a simple learning curve for risk preference. Importantly, accuracy remained significantly above chance throughout, indicating that participants could distinguish between the observees' risk and ambiguity preferences, even though learning about one could implicitly affect predictions about the other.

9. Minor: In the introduction "For instance, when choosing between a small, sure reward and a larger reward associated with uncertainty, an uncertainty-averse individual will typically select the sure one" This is not necessarily the case, unless the expected value of both the options are the same.

   Reply: We thank the reviewer for pointing out this inaccurate description. It should be "For instance, when choosing between a small, sure reward and a larger reward associated with uncertainty, an uncertainty-averse individual will typically select the sure option when the expected value of the two options is equal." To maintain precision—and because the sentence was nonessential—we deleted it from the Introduction and adjusted the surrounding text accordingly.

10. Ambiguity attitudes in Experiment 2 is muddled with risk attitudes. To measure ambiguity attitudes more precisely, it might make sense to make subjects choose between a risky and an ambiguous prospect instead of an ambiguous prospect and sure outcome (see also Dimmock et al 2016).

    Reply: We thank the reviewer for pointing to the relevant literature. We agree that choices in ambiguous decision contexts can be influenced by both risk and ambiguity preferences. However, we believe our design allows these two preferences to be disentangled for several reasons.

    First, our design closely follows established approaches in decision science studies (Hsu et al. 2005; Huettel et al. 2006; Levy et al. 2010), which have successfully delineated individual risk and ambiguity preference under a similar experimental setup using computational modelling. Consistent with these studies, we found in our study the estimated risk preference is not significantly correlated with the ambiguity preference (r = -0.22, p = 0.09; Fig. S7a).

    Second, parameter recovery analyses in our study showed that the recovered parameters for risk and ambiguity closely matched the true parameters used to generate the data, indicating that the two can be distinguished in our task (see newly added Fig. S13). Lastly, by putting risky and ambiguous prospects together, we still need to evaluate subjects' risk and ambiguity preferences for each individual from the modelling perspective.

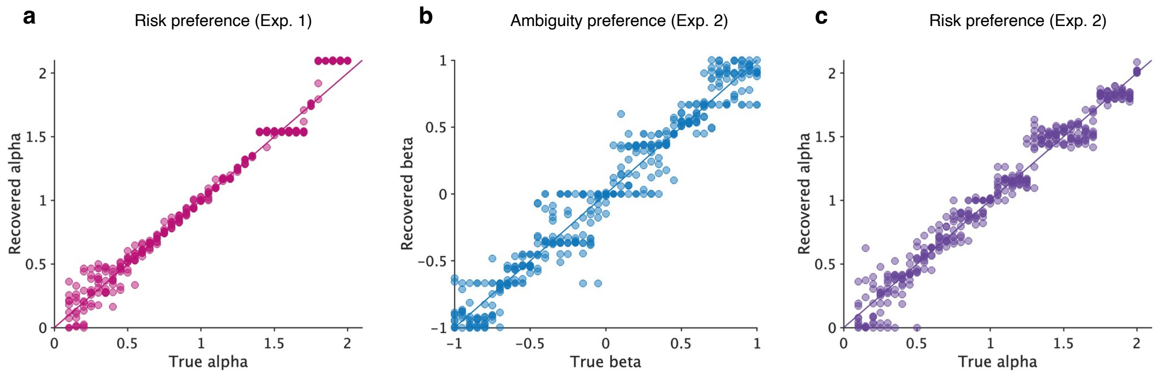

Fig. S13. Parameter recovery. Recovered parameters from model fitting are plotted against the true parameters used to generate synthetic data. Each point represents one simulation. (**a**) Recovery of risk preference parameter α in Experiment 1. (**b**) Recovery of ambiguity preference parameter β in Experiment 2. (**c**) Recovery of the risk preference parameter α in Experiment 2. In the simulations of Experiment 2, risk preference was fixed at 1 (risk-neutral) when simulating ambiguity preference, and ambiguity preference was fixed at 0 (ambiguity-neutral) when simulating risk preference. For all simulations, inverse temperature values were randomly sampled between 1 and 10. In all cases, recovered values closely track the true values, demonstrating that the model reliably distinguishes between risk, ambiguity, and stochasticity parameters.

Taken together, we believe our estimation of ambiguity preference in the second experiment is both accurate and separable from risk preference.

- **Hsu, M., Bhatt, M., Adolphs, R., Tranel, D. & Camerer, C. F. Neural systems responding to degrees of uncertainty in human decision-making. *Science (1979)* 310, 1680–1683 (2005).**
- **Huettel, S. A., Stowe, C. J., Gordon, E. M., Warner, B. T. & Platt, M. L. Neural signatures of economic preferences for risk and ambiguity. *Neuron* 49, 765–775 (2006).**
- **Levy, I., Snell, J., Nelson, A. J., Rustichini, A. & Glimcher, P. W. Neural representation of subjective value under risk and ambiguity. *J Neurophysiol* 103, 1036–1047 (2010).**

Reviewer #2:

0. Summary: Previous studies have demonstrated the social contagion effect in risky decision-making. Yet it remains unclear how the effect is modulated by disparate others and forms of uncertainty. In the current study, the authors address this question using an experimental paradigm from Suzuki et al. (2016). Participants changed their risk and ambiguity preferences towards others after observation. The social contagion effect was larger after observing risk-averse others (Experiment 1). On the other hand, no such asymmetric patterns were found in ambiguous decisions (Experiment 2).

   Basically, this study is a replication and extension of Suzuki et al. (2016): You extended the findings of contagion effects to framing and decision-making under ambiguity. While I found it interesting, I have some comments on the analyses and discussion.

   Reply: We thank the reviewer for the positive assessment of our work. Please see our replies to the following comments. Below, reviewer comments are in regular text; our replies are in bold; blue indicates the corresponding revisions in the main text and SI.

Major comments

1. You have added the gain/loss framing to Suzuki et al. (2016), which is the contribution of this study. However, the new setting introduces the problem of loss aversion and reference point (aka status quo), doesn't it? Whereas you analyzed the data as if the task were gain-only gambling like Suzuki et al. (2016), such data including loss domain are usually analyzed considering loss aversion (= the cumulative prospect theory). I would suggest reanalyzing the data (see the following paper for your reference).

   Reply: We thank the reviewer for raising this important point and for the constructive suggestion. We agree that in the loss frame, both loss aversion and uncertainty (risk/ambiguity) preference can influence participants' decisions. Following the reviewer's advice, we reanalyzed the data by introducing the loss aversion parameter into the gamble option utility calculation in the loss condition. This analysis showed that, under our current task design, including loss aversion does not alter the estimation of uncertainty preference in the loss frame. However, because the gamble options in the loss frame contained only losses (i.e., no mixed gains/losses), the loss aversion parameter and the inverse-temperature parameter cannot be estimated independently (please see explanations below and see also Huizenga et al., 2023).

   In our model, participants choose between a sure option and a gamble based on the difference in their subjective utilities, as formalized through a Softmax choice rule:

$$P(gamble) = \frac{1}{1 + e^{-\gamma(U_g - U_s)}}$$

   where U_g and U_s represent the utility of the gamble and sure option respectively. γ (inverse temperature) captures the degree of choice determinism, with γ = 0 indicating random choice and larger positive values indicating more deterministic choice toward the higher-utility option.

   Take the risk decision making as an example, we computed utilities in the gain and loss frames following cumulative prospect theory (Tversky & Kahneman, 1992; Sokol-Hessner et al., 2019; Huizenga et al., 2023):

$$U = \begin{cases} P \times R^{\alpha}, & if\ R \geq 0 \\ -\lambda \times P \times (-R)^{\alpha}, & if\ R < 0 \end{cases}$$

where P is outcome probability, R is outcome magnitude, and α captures risk preference ($\alpha = 1$ for risk-neutral, $\alpha < 1$ for risk-averse, $\alpha > 1$ for risk-seeking). The parameter λ represents the relative weighting of losses to gains ($\lambda = 1$ means gain–loss neutrality; $\lambda > 1$ means loss aversion; $\lambda < 1$ means loss seeking).

For the gain frame, the choice function becomes:

$$P(gamble) = \frac{1}{1 + e^{-\gamma(P_g R_g^{\alpha} - P_s R_s^{\alpha})}}$$

For the loss frame:

$$P(gamble) = \frac{1}{1 + e^{-\gamma \cdot (-\lambda) \cdot (P_g (-R_g)^{\alpha} - P_s (-R_s)^{\alpha})}}$$

where P_g and P_s indicate the outcome probability of the gamble and sure option respectively, and R_g and R_s represent the outcome magnitude of the gamble and sure option, respectively.

Because in our loss frame both options involve only losses, λ and γ are mathematically confounded — changes in λ can be offset by changes in γ. This confounding prevents independent estimation of λ, but does not affect the estimation of α, the risk preference parameter. If we want to separate γ and λ, we could either ask the same participant to complete both the gain and loss frame tasks or change the design to include both gain and loss in the gamble option (e.g., sure option: lose 5 for sure, and gamble option: gain 5 for 50% and lose 15 for 50%).

The same can also be applied to ambiguity preference. In particular, the utility of an option in the loss frame incorporating the loss aversion becomes:

$$U = -\lambda \times (P + \beta \times \frac{A}{2}) \times (-R)^{\alpha}$$

where P is the objective probability (0.5 for ambiguous gambles) and A represents the ambiguity level (0 means risky gambles). β is a free parameter reflecting the ambiguity preference. In the current design, the ambiguity level A varies on a trial-by-trial basis, allowing us to estimate the ambiguity preference without confounding with other parameters (see also parameter recovery for α and β in Fig. S13).

Therefore, given that loss aversion was not the primary focus of the current study, we report this consideration in the Discussion and highlight that examining the contagion effect of loss aversion, and its interaction with risk and ambiguity preferences, is an interesting direction for future work.

"In addition, future studies could directly examine how social contagion interacts with loss aversion by incorporating mixed gambles that allow independent estimation of both uncertainty (risk and ambiguity) and valence (loss aversion) preferences[29,58,59]."

References:

29. Tversky, A., & Kahneman, D. (1992). Advances in prospect theory: Cumulative representation of uncertainty. Journal of Risk and Uncertainty, 5(4), 297–323.
58. Sokol-Hessner, P., & Rutledge, R. B. (2019). The Psychological and Neural Basis of Loss Aversion. Current Directions in Psychological Science, 28(1), 20–27.

59. Huizenga, H. M., Zadelaar, J. N., Jansen, B. R. J., Olthof, M. C., Steingroever, H., Dekkers, L. M. S., Van Duijvenvoorde, A. C. K., Figner, B., & Agelink Van Rentergem, J. (2023). Formal models of differential framing effects in decision making under risk. Decision, 10(3), 197–234.

2. Including this study and Suzuki et al. (2016), many neuroscience-based studies of social influence only look at one-way influence (influence from an observee to an observer). This is methodologically straightforward, but there is still a significant gap in understanding actual social behavior. It would be better to discuss the potential importance of looking at bidirectional social interaction processes as a future direction. For example, the following references are a few examples that took such a bidirectional approach.

   - Mahmoodi et al. (2018). Reciprocity of social influence.
   - Kuroda et al. (2022). Behavioral and neuro-cognitive bases for emergence of norms and socially shared realities via dynamic interaction.

   Reply: We thank the reviewer for highlighting this limitation in the current and other related studies, and for pointing us toward relevant literature. We agree that our focus on one-way interactions represents only a subset of the broader and more complex bidirectional processes that characterize real-world social behavior. Studying unidirectional influence, as in the present work, provides a methodologically tractable starting point for isolating key mechanisms, but it does not capture the full reciprocity and dynamic feedback inherent in social decision-making.

   We concur that investigating bidirectional influence is a critical next step, as it more closely reflects naturalistic social exchanges. Indeed, recent studies, including our own (Jiang, Mi & Zhu, 2023), have begun adopting such approaches to examine the emergence of shared norms, mutual adaptation, and the dynamic interplay of influence (e.g., Mahmoodi et al., 2018; Kuroda et al., 2022). We have added a paragraph in the Discussion to emphasize this important direction for future research.

   "Since our study adopted a one-way observation experimental design, it may not capture the full complexity of social influence as real-life scenarios often involve bidirectional social communication. Bidirectional social interactions with reciprocal feedback, as shown by Mahmoodi et al. (2018), could involve mutual behavioral adjustment and Kuroda et al. (2022) demonstrated that such dynamics could yield convergence toward stable, shared norms[50,55]. Incorporating such two-way paradigms in future research could increase the ecological validity of the social contagion effect of uncertainty preferences and shed light on how reciprocal influence shapes group decision-making and collective intelligence[56,57]."

   References:
50. Kuroda, K. *et al.* Behavioral and neuro-cognitive bases for emergence of norms and socially shared realities via dynamic interaction. *Commun Biol* **5**, 1–13 (2022).
55. Mahmoodi, A., Bahrami, B. & Mehring, C. Reciprocity of social influence. *Nat Commun* **9**, 1–9 (2018).
56. Jiang, Y., Mi, Q. & Zhu, L. Neurocomputational mechanism of real-time distributed learning on social networks. *Nature Neuroscience 2023 26:3* **26**, 506–516 (2023).
57. Kameda, T., Toyokawa, W. & Tindale, R. S. Information aggregation and collective intelligence beyond the wisdom of crowds. *Nature Reviews Psychology 2022 1:6* **1**, 345–357 (2022).

3. I found the 2nd paragraph in the discussion somewhat speculative (p. 16, line 427). For example, such an asymmetric effect was also found in the aforementioned study by Kuroda et al. (2022). They didn't use a gambling task but dot-estimation task. However, they found that participants had underestimation biases and that participants showed a stronger contagion when paired with a partner with an underestimation bias than a partner with an overestimation bias (Fig. S12 in Kuroda et al.). This pattern seems quite similar to the current study, doesn't it? I think we can understand these results as a process where people become more similar to others who are already somewhat similar to themselves (Fig. 1d). Or, you could discuss it in the context of literature on confirmation (e.g., refs. 13–17 you cited).

   Reply: We thank the reviewer for highlighting the potential similarity between our findings and those of Kuroda et al. (2022), which used a dot-estimation task to investigate the neurocognitive mechanisms of bidirectional social influence. While there are some parallels, we believe the behavioral patterns they observed differ in important ways from the asymmetric contagion effect reported in our study.

   In the online experiment of Kuroda et al., participants estimated the number of dots on a screen before and after interacting with another agent. Importantly, during interaction, participants could see the agent's estimate in addition to making their own. Two types of agents were used: "Asch-type" agents, who never changed their estimates (one-way influence), and "Sherif-type" agents, who gradually adjusted their estimates toward the participant's (bidirectional influence). Because the observees in our study functionally resemble Asch-type agents, we restrict our comparison to that condition. When paired with an Asch-type partner, participants decreased their estimation weights after interacting with under-estimators and increased them after interacting with over-estimators—consistent with the social-influence pattern we observe. Although Kuroda et al.'s data appear to show a larger shift toward Asch-type under-estimators than over-estimators, the statistical comparison of the contagion effects between the under- and over-estimators were not reported.

   Moreover, our asymmetric contagion effect in risk preference cannot solely be explained by confirmation bias. In our congruence–incongruence analysis (Fig. S6b), we divided participants based on whether their initial uncertainty preference matched that of the observee (e.g., both risk-averse or both risk-seeking). If confirmation bias were driving the effect, contagion should be stronger when preferences are congruent than when they are incongruent. However, we found no significant difference in contagion between congruent and incongruent preferences, nor did we observe a similar asymmetry in ambiguity preference, which would be expected if confirmation bias were at play.

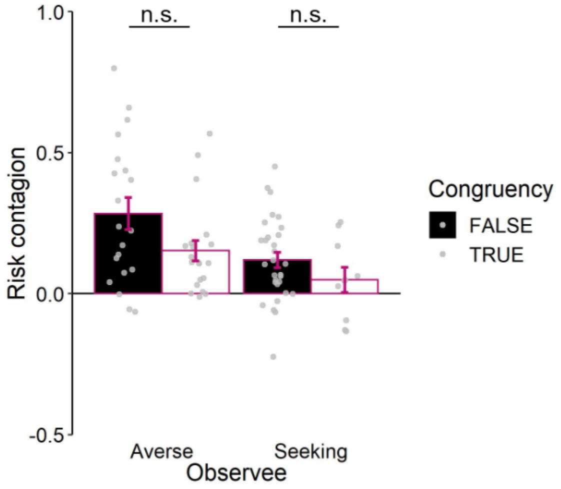

Risk contagion as a function of observees' risk-preference type and the congruency between the participant and the observee (Mean ± SEM across participants within each group). Gray dots represent the risk contagion of each participant when their risk preference is either congruent (white) or incongruent (black) with the observees. ANOVA analysis revealed neither the main effect of the congruency between the participants' and the observees' risk preference ($F(1,38) = 1.22$, $p = 0.276$) nor the interaction effect ($F(1,38) = 0.08$, $p = 0.784$) was significant. Post hoc analyses using the Wilcoxon rank sum test, given the unequal distribution of the data, revealed no effects of congruency when the observee was either risk-averse ($W = 255.5$, $p = 0.081$) or risk-seeking ($W = 237.5$, $p = 0.075$). ***$p < 0.001$; **$p < 0.01$; *$p < 0.05$; n.s., not significant.

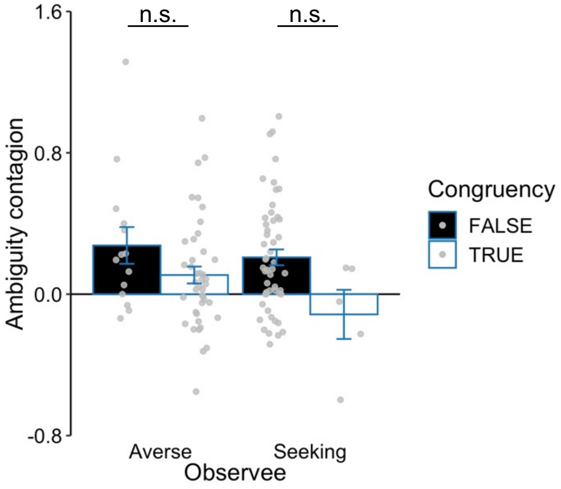

Ambiguity contagion as a function of observees' ambiguity preference type and the congruency between the participant and the observee (mean ± SEM across participants within each group). Gray dots represent the ambiguity contagion of each participant when their ambiguity preference is congruent (white) or incongruent (black) with the observees. ANOVA analysis revealed that the ambiguity contagion effect was also stronger when the preferences between participants and observees were incongruent (main effect of the congruency $F(1,53) = 4.29$, $p = 0.030$), but the interaction effect (main effect of the congruency was not significant ($F(1,53) = 0.149$, $p = 0.304$). Post hoc analyses using the Wilcoxon rank sum test, given the unequal distribution of the data, revealed no effects of congruency when the observee was

either ambiguity-averse ($W = 374$, $p = 0.134$) or ambiguity-seeking (W= 190, $p = 0.075$). ***$p < 0.001$; **$p < 0.01$; *$p < 0.05$; n.s., not significant.

Although we agree that confirmation bias is a potential driving force for most social influences, we suggest that the asymmetric contagion effect for risk preference may reflect a conservative bias, similar to the effect reported by Ihssen et al. (2016), which found that participants in an observational learning task weighted others' conservative choices more heavily than impulsive ones when making their own decisions. Notably, this conservative bias did not extend to ambiguity preference in our study, highlighting an intrinsic difference between how risk and ambiguity preferences are socially transmitted.

While we do not yet have a definitive explanation for these differences, we speculate that distinct neurocomputational mechanisms may underlie risk and ambiguity contagion. We believe further research into these differences will provide valuable insights into the neurocomputational basis of decision-making under risk and ambiguity. We have revised our discussion to incorporate the reviewer's insightful points discussed above.

"Previous studies have shown that individuals are strongly influenced by others perceived as similar, such as those sharing common hobbies, preferences or perceptual bias[15,50]. This theory would predict participants adjusting their behaviors more closely to observees with similar behavioral preferences, i.e., a confirmation bias. However, our findings do not fully support this explanation. Participants tended to shift their preferences more towards individuals with dissimilar preferences (Fig. S6b and S9b), consistent with previous studies[5]. We propose that this asymmetric contagion effect in risk preference may reflect a conservative bias. This interpretation aligns with Ihssen et al. (2016), who found that participants in an observational learning task gave greater weight to others' cautious choices than to impulsive ones when forming their own decisions[19]. Importantly, this conservative bias did not extend to ambiguity preferences in our study, suggesting a fundamental difference in how risk and ambiguity preferences are socially transmitted."

5. Suzuki, S., Jensen, E. L. S., Bossaerts, P. & O'Doherty, J. P. Behavioral contagion during learning about another agent's risk-preferences acts on the neural representation of decision-risk. Proc Natl Acad Sci U S A 113, 3755–3760 (2016).
15. Kappes, A., Harvey, A. H., Lohrenz, T., Montague, P. R. & Sharot, T. Confirmation bias in the utilization of others' opinion strength. *Nat Neurosci* **23**, 130–137 (2020).
19. Ihssen, N., Mussweiler, T. & Linden, D. E. J. Observing others stay or switch – How social prediction errors are integrated into reward reversal learning. Cognition 153, 19–32 (2016).
50. Kuroda, K. *et al.* Behavioral and neuro-cognitive bases for emergence of norms and socially shared realities via dynamic interaction. *Commun Biol* 5, 1–13 (2022).

4. I know Gilboa & Schmeidler's maximin expected utility model, but I'm curious about its prediction accuracy. How well did it reproduce participants' behavior? I'd like to see the prediction accuracy index or visualization, which is something like posterior predictive checking in the context of Bayesian parameter estimation.

Reply: We thank the reviewer for raising the question regarding the reliability of Gilboa & Schmeidler's maxmin utility model in capturing participants' choices in ambiguity-based decision-making. While our approach follows prior studies that have used this model to

simultaneously estimate risk and ambiguity preferences, it is indeed important to demonstrate its predictive accuracy and parameter separability in the current dataset.

To address this, we conducted the following complementary analyses:

(i) Model fitting — The maxmin utility model predicted 85.71% of participants' choices of each session in the original experiment (see the boxplot below).

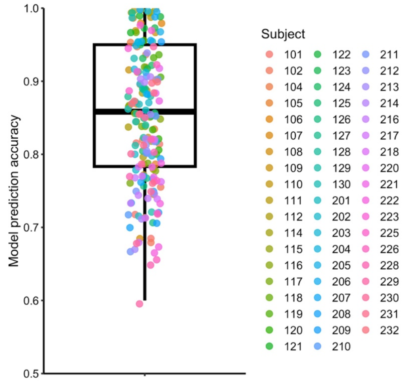

(ii) Parameter recovery — The recovered parameters (risk preference and ambiguity preference) showed strong correlations with the parameters used to generate the data, confirming they are separable and estimated reliably (also see Question 10 of Reviewer 1).

We have added the following sentences into the main text:

"Under the current design, 85.71% of participants' choices in the ambiguous decision contexts could be accurately predicted by the maxmin expected utility model and no correlation were observed between the estimated preferences (Fig. S7a; see also Fig. S13 for parameter recovery)."

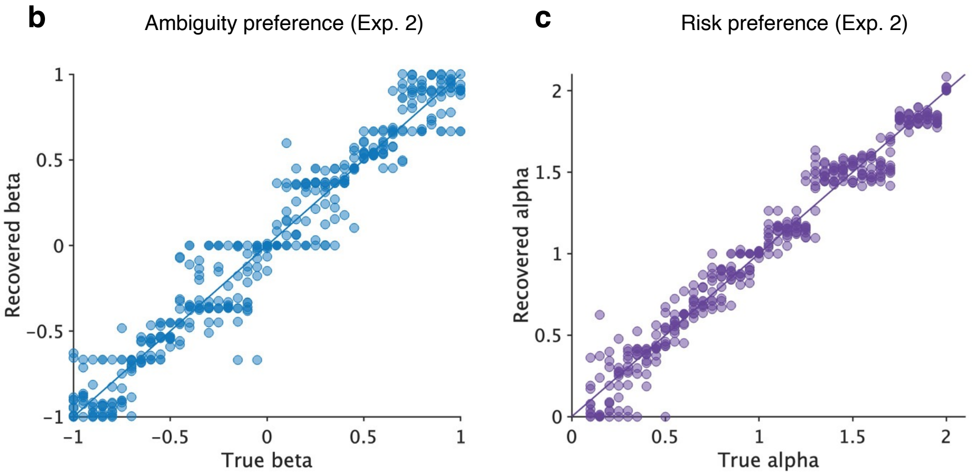

Fig. S13. Parameter recovery. Recovered parameters from model fitting are plotted against the true parameters used to generate synthetic data. Each point represents one simulation. (**b**) Recovery of ambiguity preference parameter β in Experiment 2. (**c**) Recovery of the risk preference parameter α in

Experiment 2. In the simulations of Experiment 2, risk preference was fixed at 1 (risk-neutral) when simulating ambiguity preference, and ambiguity preference was fixed at 0 (ambiguity-neutral) when simulating risk preference. For all simulations, inverse temperature values were randomly sampled between 1 and 10. In all cases, recovered values closely track the true values, demonstrating that the model reliably distinguishes between risk, ambiguity, and stochasticity parameters.

5. I don't usually comment on gender differences, but risky decision-making usually has a large gender difference. It's not necessary to do detailed additional analyses, but for more transparency (and given that you used only male observees), it would be a good idea to show gender differences in risk preference, ambiguity preference, and contagion effect at least.

Reply: Following the reviewer's suggestions, we further split the participants based on their reported genders.

Initial risk preference (Session 1, before observation): In Experiment 1, female participants are more risk-averse than male participants (two-sample t-test: *t*(37.712) = -2.439, *p* = 0.020; panel a). In Experiment 2, female participants are also more risk-averse than male participants (*t*(50.98) = -2.273, *p* = 0.031; panel b right), consistent with previous research (Khor et al., 2020; Rai & Kimmel, 2015; Sarin & Wieland, 2012). There is no gender difference between male and female participants' ambiguity preferences (*t*(50.98) = -0.314, *p* = 0.755; panel b left).

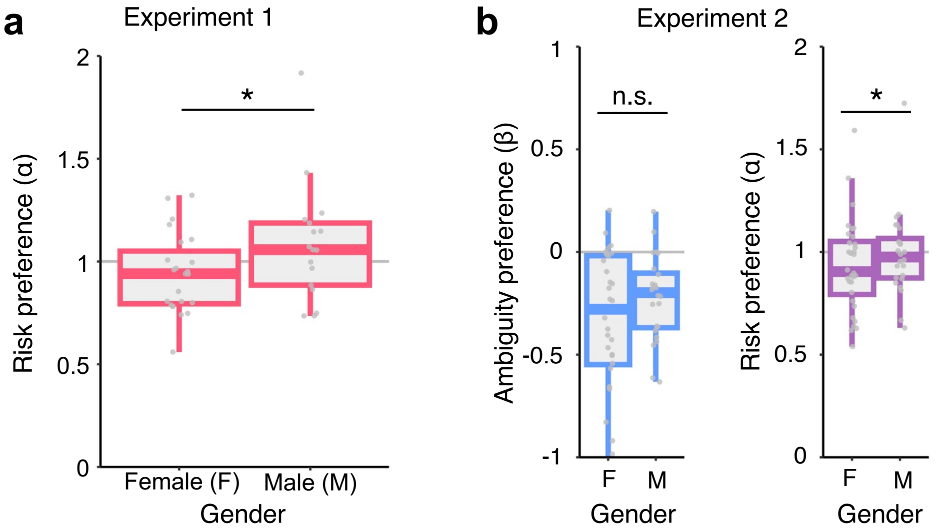

- **Khor, L. Y., Sariyev, O., & Loos, T. (2020). Gender differences in risk behavior and the link to household effects and individual wealth. *Journal of Economic Psychology*, *80*, 102266.**
- **Rai, J., & Kimmel, J. (2015). Gender Differences in Risk Preferences: An Empirical Study using Attitudinal and Behavioral Specifications of Risk Aversion. *Research in Labor Economics*, *42*, 61–91.**
- **Sarin, R. K., & Wieland, A. M. (2012). Gender Differences in Risk Aversion: A Theory of When and Why. *SSRN Electronic Journal*.**

Risk contagion effect (Experiment 1): A two-way mixed ANOVA revealed a consistent asymmetric risk contagion effect in both the male and female participants: subjects were more aligned with risk-averse observees than with risk-seeking ones ($F(1,35) = 30.329$, $p < 0.001$). Neither the main effect of the gender ($F(1,35) = 1.868$, $p = 0.180$) nor the interaction effect ($F(1,35) = 1.813$, $p = 0.284$) was statistically significant.

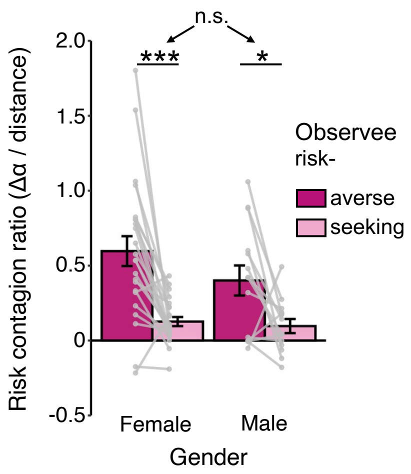

Ambiguity contagion effect (Experiment 2): Analysis on ambiguity contagion ratio showed that neither the observee's ambiguity preference ($F(1,52) = 0.73$, $p = 0.397$), participants' gender ($F(1,52) = 2.774$, $p = 0.102$), nor the interaction effects affected the ambiguity contagion ratio ($F(1,52) = 0.462$, $p = 0.500$).

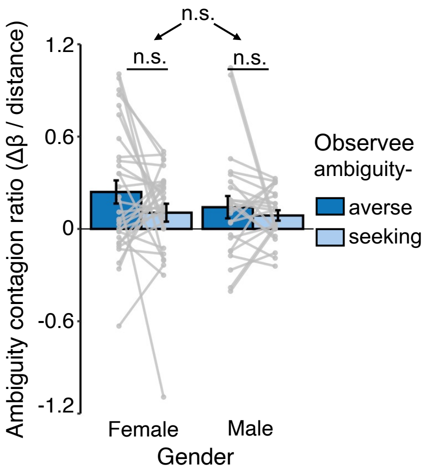

These results suggest that the contagion patterns we observed in both risk and ambiguity are consistent across male and female participants. We have added these results into the supplementary figures (Fig. S14).

Minor comments

6. For clarification: In p.4, line 112, "… chose less risky gambles than those in the Loss frame" should be "… chose gambles less often than in the Loss Frame"? "less risky gambles" sounds not accurate.

Reply: We thank the reviewer for pointing out the inaccurate wording. We have revised the sentence to read:

"We found that participants in the Gain frame initially chose risky gambles less often than those in the Loss frame."

7. In p.20, line 546: You write "his/her choice" in the line, but the observees were both male. This might be a gender-sensitive expression, but it could be a little confusing for readers. In the Experiment 2, did you instruct participants that "you (participants) will experience the same gambles that observees experienced"? I.e., did you tell participants that the ground truth (the true probability) in the ambiguous gambles are same between participants and observers? Please clarify. This point may have affected their decisions.

   Reply: The reviewer is correct that both observees were photos of males. To avoid confusion, we have revised the text to read "observe their choices" to maintain gender neutrality. In experiment 2, we instruct subjects that observees completed the same task as the participants did in the Self trials (in reality, the observees were simulated agents and subjects were debriefed after the end of the experiments). This information allowed participants to infer that the objective probabilities for the ambiguous gambles was identical for both themselves and the observees. In addition, following suggestions from other reviewers, we performed a preregistered replication study in which we used cartoon images for the observee instead of photographs of real individuals, and fully replicated our main findings in the original study.

8. Most of the figures in the supplementary information are somewhat blurred to me. They are still readable but could be improved.

   Reply: We thank the reviewer for noting the issue with figure clarity in the Supplementary Information. The blurriness was caused by a formatting issue during the conversion from Word to PDF. We have corrected this problem and updated the supplementary file with higher-resolution figures.

Reviewer #3:

0. In this manuscript, the authors examine how decision makers are influenced by observing simulated choices in the domain of risk and ambiguity. As a result, participants are influenced by the observed choices in both domains. In addition, there is asymmetry in that participants are more influenced by simulated risk-averse choices than risk-seeking choices in the domain of risk, while ambiguity-averse and ambiguity-seeking simulated choices affect participants equally in the domain of ambiguity.

   In sum, I think this is an interesting research question, the authors present two original experiments, and overall, the manuscript is well-written. However, I have some comments concerning potential limitation of the design and the analyses, which I outline in the following.

   Reply: We thank the reviewer for the positive words. Please see our replies to the following comments. Below, reviewer comments are in regular text; our replies are in bold; blue indicates the corresponding revisions in the main text and SI.

1. Within-design of asking participants to predict the choices of "others" and making personal decisions: I think a disadvantage of the current design is that blocks with predictions of what the "other" chose and blocks with personal decisions are presented to participants alternatingly and in close proximity. Therefore, I think a psychologically not very interesting alternative explanation other than social contagion, could be that participants just confused the personal decision-making with the prediction task. Can the authors provide any evidence to alleviate this concern through, for example, attention checks asking about the current task, or a design in which predictions and personal decisions are separated more rigorously? I think, it is important to at least discuss this alternative explanation.

   Reply: We thank the reviewer for raising this alternative explanation regarding our main findings. In line with the reviewer's suggestion, we conducted a preregistered replication study in which we (i) added attention checks at the end of each mini-block, asking participants to identify the task they had just completed, and (ii) replaced the simulated choices of the observees with choices made by real individuals, thereby removing any deception.

   Modifications in replication study. The design of the replication study remained the same as the original study except for the following three modifications. First, to enhance ecological validity and avoid potential demand effects, we used recorded behavior from two actual human participants instead of using simulated observees as in the original study. To be consistent with the original study as much as possible, the behavioral patterns of the chosen observees in the replication study closely matched those simulated observees used in the original study. Second, to protect the observees' privacy, we replaced the original back-view photographs with cartoon icons representing the observees. These cartoon photos can also mitigate the influence of observees' gender on the contagion effect. Third, we included a new trial-type judgment task at the end of each mini-block to help rule out the possibility that the observed behavioral changes are due to participants' confusion between Self and Prediction trials, rather than genuine social contagion effects. All these modifications were pre-registered (https://osf.io/f4wuy).

The replication study reproduced the main results of the original study, with asymmetric contagion for risk preferences and symmetric contagion for ambiguity preferences (Fig. 2g-i and Fig. 4g-i). Importantly, participants demonstrated near-perfect accuracy in identifying the task they had just completed (risk contagion experiment: 99.4%; ambiguity contagion experiment: 99.8%), indicating that they could reliably distinguish between block types. Furthermore, attention-check accuracy was not correlated with the magnitude of the contagion effect.

Taken together, these findings suggest that the observed contagion effects are unlikely caused by the confusion between the prediction and personal decision-making tasks.

2. Deception of participants: Relatedly, participants were deceived in that the observed choices stem from a computer simulation rather than a real human participant. Can the authors justify why this was necessary? In my view, such deception can compromise the quality of the research data if participants inform future participants about the deceptions (during one experiment, but also across experiments as a lab reputation). In principle, it seems to be possible to collect data from participants in this task and then select some of them that differ in risk taking to participants.

   Reply: We appreciate the reviewer's thoughtful comments on this issue (see also Question 5 from Reviewer 1 for a similar concern). The original design followed established protocols used in prior studies (Suzuki et al. 2016), which employed simulated observee choices rather than data from real participants. They showed that participants display similar behavioural patterns regardless of whether the observee is a simulated agent or a real person, suggesting that data quality is unlikely to be compromised significantly by this approach.

   - **Suzuki, S., Jensen, E. L. S., Bossaerts, P. & O'Doherty, J. P. Behavioral contagion during learning about another agent's risk-preferences acts on the neural representation of decision-risk. Proc Natl Acad Sci U S A 113, 3755–3760 (2016).**

   That said, we agree that avoiding deception is preferable whenever possible. Following the reviewer's suggestion, we conducted a preregistered replication study in which the observees' choices were drawn from actual human participants rather than from simulation (also see Modifications in Replication Experiments for more details). The replication study fully replicated the original findings, confirming the robustness of the observed asymmetric contagion for risk and symmetric contagion for ambiguity preferences. Further discussion of this issue can be found in our response to Question 5 of Reviewer 1.

   "**Modifications in replication study.** The design of the replication study remained the same as the original study except for the following three modifications. First, to enhance ecological validity and avoid potential demand effects, we used recorded behavior from two actual human participants instead of using simulated observees as in the original study."

3. Using a stochastic power utility model for main inference: The stochastic power utility model is known to produce parameter correlations and can have poor estimation accuracy in some choice problem sets (Krefeld-Schwalb et al., 2022; Olschewski et al., 2022). Therefore, I think it is important to build trust in the reported model-based analyses. I would suggest to either also present and interpret the model-free analyses in the main task. Alternatively, the authors could perform a model recovery to estimate the

measurement accuracy of the utility curvature parameter in their data set and discuss what this means for the interpretation of the results.

Reply: We thank the reviewer for highlighting the importance of establishing the reliability of the power utility model (see also Question 4 from Reviewer 2 for a similar suggestion). We agree that it is important to verify model estimates, even though stochastic power utility models have been widely applied in previous research.

For Experiment 1 (risk preference), we compared the results between model-based approach using power utility model and a model-free analysis (Fig. 2 and S4). Both approaches produced consistent results, confirming that the main findings of the first experiment are robust across analysis methods. We report the power utility model in the Main Text to maintain consistency with Experiment 2, where this model is used to dissociate risk and ambiguity preferences.

Following the reviewer's suggestions, we further evaluated the reliability of the power utility model by (i) examining correlations between different parameters, and (ii) conducting parameter recovery analyses. These results (see also our reply to Question 4 by Reviewer 2) confirm that the model accurately estimates risk and ambiguity preferences and that the parameters are distinguishable.

We found that there are no correlations between different parameters. In Experiment 1, participants' estimated initial risk preferences and inverse temperatures (from softmax function) are not correlated with each other (Pearson's r = -0.069, p = 0.647). In Experiment 2, participants' estimated initial risk preferences and ambiguity preferences are not correlated (Pearson's r = -0.217, p = 0.089; Fig. S7a); nor were correlations between initial risk preferences and inverse temperature (Pearson's r = 0.040, p = 0.759); or between initial ambiguity preferences and inverse temperature (Pearson's r = -0.049, p = 0.703).

The recovered parameters (risk preference and ambiguity preference) showed strong correlations with the parameters used to generate the data, confirming they are separable and estimated reliably (also see Question 10 of Reviewer 1).

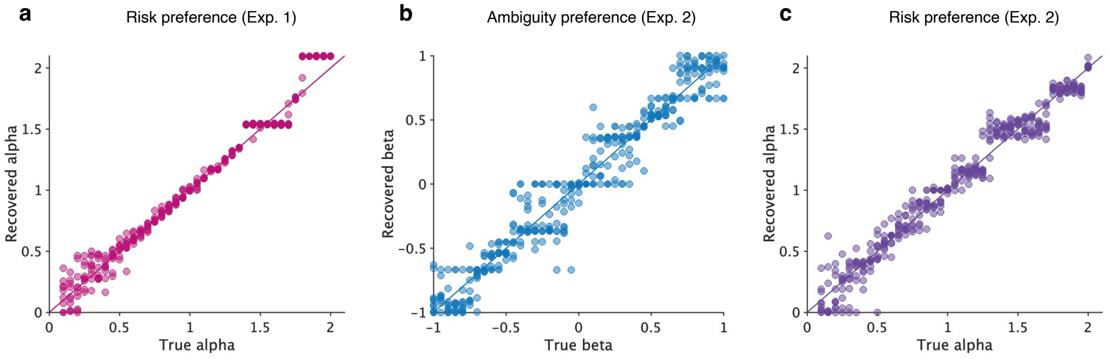

Fig. S13. Parameter recovery. Recovered parameters from model fitting are plotted against the true parameters used to generate synthetic data. Each point represents one simulation. (**a**) Recovery of risk preference parameter α in Experiment 1. (**b**) Recovery of ambiguity preference parameter β in Experiment 2. (**c**) Recovery of the risk preference parameter α in Experiment 2. In the simulations of Experiment 2, risk preference was fixed at 1 (risk-neutral) when simulating ambiguity preference, and ambiguity

preference was fixed at 0 (ambiguity-neutral) when simulating risk preference. For all simulations, inverse temperature values were randomly sampled between 1 and 10. In all cases, recovered values closely track the true values, demonstrating that the model reliably distinguishes between risk, ambiguity, and stochasticity parameters.

Taken together, these results support the robustness and reliability of our model-based analyses in the current study.

4. Exclusions of participants without pre-registration: I noticed that in both studies some participants were excluded based on choices or predictions. As these exclusions are based on dependent variables and were not pre-registered, I think it is important to examine the robustness of the reported findings by examining whether the results hold when including those participants.

   Reply: We thank the reviewer for bringing this concern to our attention. In response, we explicitly preregistered the criteria for participant exclusions in our replication study (see https://osf.io/f4wuy for the preregistration report). Using the same exclusion criteria as the original study, the replication fully reproduces the main findings, supporting the robustness of our results.

   Pre-registered exclusion criteria for replication study. Participants were excluded from the whole data analyses if they meet any of the following: (1) low prediction accuracy in Predict trials after the observation in at least one session (threshold = 50% chance level), (2) extremely biased or invariant choices, indicating a lack of meaningful engagement (e.g., always choosing gamble or always choosing sure option), (3) explicit disbelief in the experimental setting or suspicion about the manipulation, as revealed during debriefing, and (4) fail to pass the attention check (i.e., catch trials). Moreover, for the calculation of the contagion ratio (defined as the contagion effect divided by the preference distance between the observer and the observee), participants with zero or negative distance toward the observee (same preference as the observee or even more extreme) were excluded from the analysis. All exclusion criteria are pre-registered (https://osf.io/f4wuy).

5. Interpretation of difference between risk and ambiguity: The authors strongly interpret a difference in social contagion between risk and ambiguity. Namely, that participants show more contagion toward risk aversion than toward risk seeking, while no such asymmetry was observed in the case of ambiguity. However, risk and ambiguity were manipulated in separate experiments. Could the authors rule out that either the manipulation or the participant pool in Experiment 2 could have led to more measurement noise or that the effect size of social contagion in ambiguity is just lower than in risk, so that the authors had not enough power to detect the asymmetry for ambiguity. Potentially, Bayes factors could clarify the evidence there is against the asymmetry of social contagion in ambiguity. Alternatively, the authors could discuss the limitation of interpreting a null finding within one experiment as well as comparing between risk and ambiguity across experiments.

   Reply: We thank the reviewer for these helpful suggestions regarding the interpretation of differences between risk and ambiguity contagion (see also Question 7 by Reviewer 1 for similar comment).

   First, per the reviewer's suggestion, we calculated the Bayes factor to further support this null effect, providing evidence against an asymmetry in ambiguity contagion (Bayes Factor: *BF*=0.55).

Second, to further address this, we conducted a preregistered replication study with 62 additional participants in the ambiguity decision context. The results replicated our original findings in both the contagion ratio and regression analyses (Fig. 4g-h). We again found no significant asymmetric social contagion toward ambiguity aversion, demonstrating the robustness of our original results. Here are the replication results we added to the Main Text (also see the reply to Questions 1 and 7 of Reviewer 1):

Replication of symmetry in ambiguity contagion. We replicated the symmetric ambiguity contagion effect with another independent sample of participants in a preregistered study (N = 62, see Methods for details). ANOVA analysis on contagion ratio with the replication sample again revealed a consistent symmetric ambiguity contagion effect in both the gain and loss frames: neither the observee's ambiguity preference ($F(1,60) = 1.699$, $p = 0.197$), the decision frame ($F(1,60) = 1.142$, $p = 0.290$), nor the interaction of these two variables affected the ambiguity contagion ratio ($F(1,60) = 0.244$, $p = 0.623$, Fig. 4g), suggesting a consistent influence of observees' ambiguity preferences on participants' ambiguity contagion regardless of the decision frames and the observees' ambiguity preferences. In line with the contagion ratio analysis, linear regression analyses showed the ambiguity contagion effect was positively correlated with ambiguity distance, no matter when the observee was ambiguity-averse or ambiguity-seeking, showing no asymmetry ($coef_{\text{ambiguity-averse}} = 0.678 \pm 0.156$, $p < 0.001$; $coef_{\text{ambiguity-seeking}} = 0.469 \pm 0.106$, $p < 0.001$; $coef_{\text{ambiguity-averse}} - coef_{\text{ambguity-seeking}} = 0.209 \pm 0.186$, $p = 0.263$; Fig. 4h). We also replicated the symmetry in RT and found that RT did not change with the ambiguity distance when the observees was either ambiguity-averse or ambiguity-seeking ($coef_{\text{ambiguity-averse}} = 0.254 \pm 0.147$, $p = 0.084$; $coef_{\text{ambiguity-seeking}} = 0.192 \pm 0.129$, $p = 0.141$; $coef_{\text{ambiguity-averse}} - coef_{\text{ambguity-seeking}} = 0.062 \pm 0.199$, $p = 0.756$; Fig. 4i). These replication results confirmed the robustness of the symmetric ambiguity contagion effect discovered in the original study.

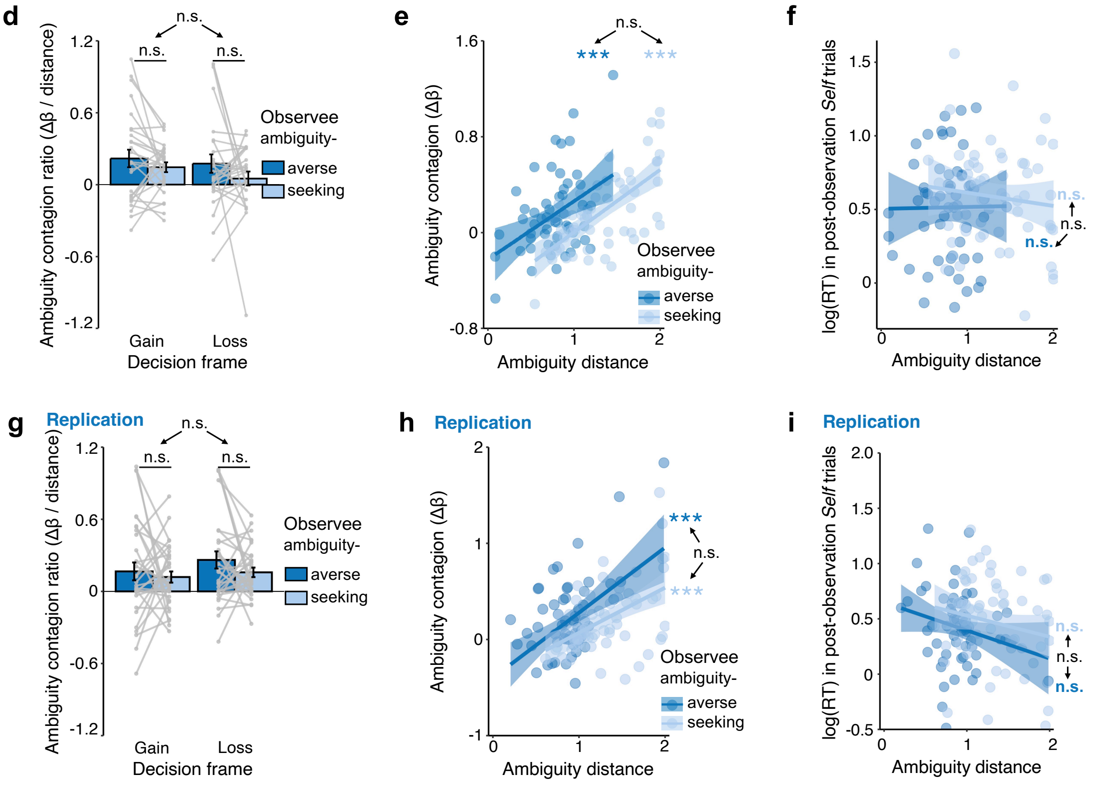

Fig. 4. Ambiguity contagion effect. (**d**) Degree of ambiguity contagion ratio as a function of the decision frame and the observee's ambiguity-preference type (mean ± SEM). Gray dots linked by a line represent the ambiguity contagion ratio of each participant when observing ambiguity-averse and ambiguity-seeking observees. (**e**) Ambiguity contagion as a function of ambiguity distance. (**f**) The log-transformed reaction time in post-observation Self trials, as a function of ambiguity distance. In (**e**) and (**f**), each blue/light-blue point represents a participant when observing the ambiguity-averse/ambiguity-seeking observee, respectively. Fitted regression lines are plotted, and the shaded area around the fitted line reflects 95% CIs. (**g-i**) The replication results for panels (**d**), (**e**), and (**f**), respectively, derived from the replication sample. ***$p < 0.001$; **$p < 0.01$; *$p < 0.05$; n.s., not significant.

Importantly, when pooling the data from the original and replication studies (*N* = 118), ambiguity contagion remained symmetric (Fig. 5c). Even with this larger sample, we still observed no statistically significant difference between ambiguity-averse and ambiguity-seeking contagion (effect size: Cohen's d = 0.256), demonstrating the absence of asymmetry in ambiguity contagion is robust and replicable.

Comparing contagion effects of risk and ambiguity preferences across replication experiments. The same analysis on our replication sample (N = 123) generated consistent results of asymmetric risk contagion and symmetric ambiguity contagion effects (uncertainty context [risk > ambiguity]: $F(1,121) = 9.715$, $p = 0.002$); observee type [averse > seeking]: $F(1,121) = 27.12$, $p < 0.001$; interaction: $F(1,121) = 16.80$, $p < 0.001$; post hoc t-test in risk context: $t_{averse>seeking}(121) = 6.577$, $p < 0.001$; post hoc t-test in ambiguity context: $t_{averse>seeking}(121) = 0.810$, $p = 0.419$; Fig. 5b). After pooling the data from both original and replication experiments (N = 219), the results remained the same (uncertainty context [risk > ambiguity]: $F(1,217) = 18.38$, $p < 0.001$; observee type [averse > seeking]: $F(1,217) = 47.41$, $p < 0.001$; interaction: $F(1,217) = 26.43$, $p < 0.001$; post hoc t-test in risk context: $t_{averse>seeking}(217) = 8.464$, $p < 0.001$;

post hoc *t*-test in ambiguity context: $t_{averse>seeking}(217) = 1.486$, $p = 0.139$; Fig. 5c), demonstrating the robustness of the results.

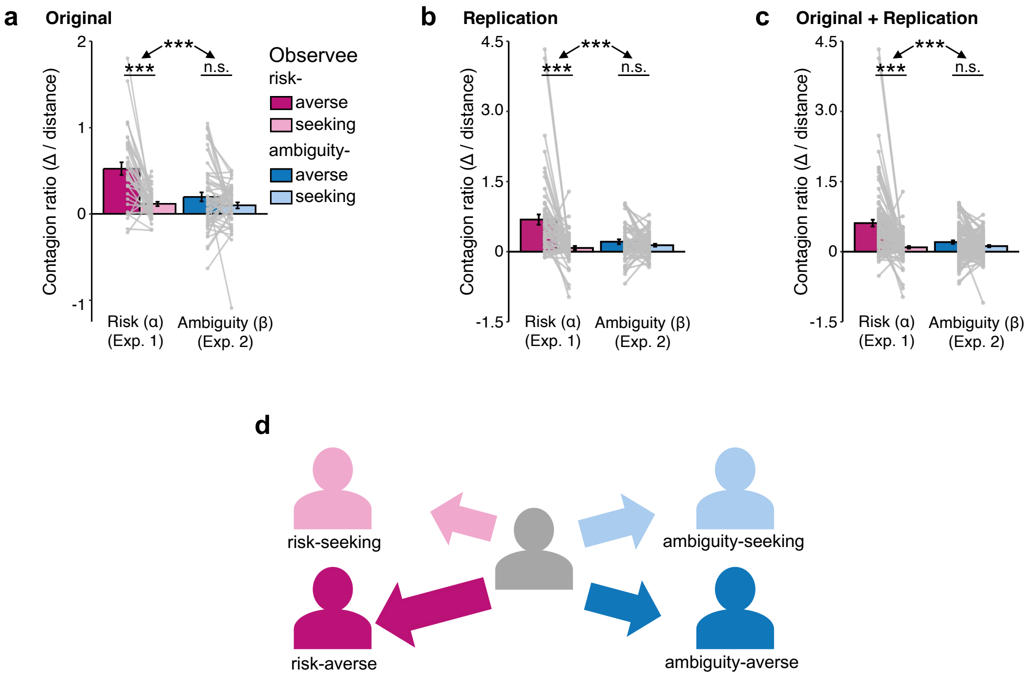

Fig. 5. Asymmetric risk and symmetric ambiguity contagion effects across two experiments. (**a**) Model-based contagion ratio of risk preference (*α* in Exp. 1) and ambiguity-preference (*β* in Exp. 2) as a function of the observees' risk/ambiguity preferences (mean ± SEM). Gray dots linked by a line represent the risk/ambiguity contagion of each participant when observing risk-averse/seeking and ambiguity-averse/seeking observees. The uppermost asterisk indicates that the interaction effect between uncertainty type (risk/ambiguity) and observees' uncertainty preference is significant. (**b**) The replication results from the replication sample. (**c**) The results after pooling the data from both original and replication studies. (**d**) Summary illustration of an asymmetric risk contagion effect (stronger risk-averse contagion than risk-seeking contagion) and symmetric ambiguity contagion effect (comparable ambiguity-averse and ambiguity-seeking contagion). ***$p < 0.001$; **$p < 0.01$; *$p < 0.05$; n.s., not significant.

Third, in Experiment 2, regression analyses show that ambiguity contagion increases with ambiguity distance for both observee types, with comparable slopes (beta coefficients: $coef_{ambiguity\text{-}averse} = 0.486 \pm 0.135$, $p < 0.001$; $coef_{ambiguity\text{-}seeking} = 0.522 \pm 0.082$, $p < 0.001$). These effects are similar in magnitude to the risk-averse slope in Experiment 1 ($coef_{risk\text{-}averse} = 0.453 \pm 0.106$, $p < 0.001$) and clearly larger than the non-significant risk-seeking slope ($coef_{risk\text{-}seeking} = 0.068 \pm 0.135$, $p = 0.613$). Thus, this complementary analysis indicates that ambiguity contagion is equally strong when observing ambiguity-averse and ambiguity-seeking observees, with no detectable difference—confirming the absence of asymmetry effect.

Additionally, even though Exp. 2 was primarily designed to investigate ambiguity contagion, we still replicated a similar pattern of asymmetric risk contagion, with a smaller but significant effect (Fig. S8c), where participants showed a stronger contagion ratio when observing risk-averse observees than risk-seeking ones. This demonstrates that asymmetric risk contagion and

symmetric ambiguity contagion can co-occur within the same experiment, not only across experiments.

Taken together, these results indicate that the symmetric ambiguity contagion effect is robust, replicable, and not due to lower effect size or experimental limitations. Therefore, the observed difference between risk and ambiguity contagion likely reflects genuine differences in how participants are influenced under risk versus ambiguity.

6. Presenting all created choice problems in a table: I could not fully understand the created choice problems, although they were explained in detail in the methods and in some additional figures. Maybe it would be easiest to add a table with all choice problems and their characteristics?

Reply: We thank the reviewer for highlighting the need for a clearer description of our choice problem design and for the constructive suggestion. We apologize for any confusion caused by the previous description. Following the reviewer's recommendation, we have now included two tables in the supplementary information that list all choice problems along with their relevant details (see Supplementary Tables 1 and 2 for details). This should allow readers to fully understand the design of the options used in our study.

Supplementary Table 1. Task design and observees' choices in Experiment 1 (risk contagion).

Initial value	Gamble option	Sure option		Obervees choosing gamble	
	Probability (keep all)	Keep (Gain Frame)	Lose (Loss Frame)	risk-averse	risk-seeking
20	0.3	10	10	0	0
20	0.4	10	10	0	1
20	0.5	10	10	0	1
20	0.6	10	10	0	1
20	1	10	10	1	1
30	0.3	10	20	0	1
30	0.4	10	20	0	1
30	0.4	20	10	0	0
30	0.5	10	20	1	1
30	0.5	20	10	0	0
30	0.6	10	20	1	1
30	0.6	20	10	0	0
30	0.7	20	10	0	1
40	0	30	10	0	0
40	0.4	20	20	0	1
40	0.4	30	10	0	0
40	0.5	20	20	0	1
40	0.5	30	10	0	0
40	0.6	20	20	0	1
40	0.6	30	10	0	1
40	0.7	20	20	1	1

40	0.7	30	10	0	1
50	0.4	30	20	0	1
50	0.5	20	30	0	1
50	0.5	30	20	0	1
50	0.6	20	30	1	1
50	0.6	30	20	0	1
50	0.7	30	20	0	1

Supplementary Table 2. Task design and observees' choices in Experiment 2 (ambiguity contagion).

Initial value	Gamble option		Sure option		Obervees choosing gamble option	
	Probability (keep all)	Ambiguity	Keep (Gain)	Lose (Loss)	ambiguity-averse (slightly risk-seeking)	ambguity-seeking (slightly risk-averse)
20	0.25	0	10	10	0	0
30	0.25	0	10	20	0	0
30	0.25	0	20	10	0	0
40	0.25	0	20	20	0	0
60	0.25	0	30	30	0	0
20	0.5	0	10	10	1	0
30	0.5	0	10	20	1	1
30	0.5	0	20	10	0	0
40	0.5	0	20	20	1	0
60	0.5	0	30	30	1	0
20	0.75	0	10	10	1	1
30	0.75	0	10	20	1	1
30	0.75	0	20	10	1	1
40	0.75	0	20	20	1	1
60	0.75	0	30	30	1	1
20	0.5	0.24	10	10	0	1
30	0.5	0.24	10	20	1	1
30	0.5	0.24	20	10	0	0
40	0.5	0.24	20	20	0	1
60	0.5	0.24	30	30	0	1
20	0.5	0.5	10	10	0	1
30	0.5	0.5	10	20	0	1
30	0.5	0.5	20	10	0	1
40	0.5	0.5	20	20	0	1
60	0.5	0.5	30	30	0	1
20	0.5	0.74	10	10	0	1
30	0.5	0.74	10	20	0	1
30	0.5	0.74	20	10	0	1

40	0.5	0.74	20	20	0	1
60	0.5	0.74	30	30	0	1

Reviewer #1:

Dear Authors,

0. Thank you for the excellent revision and for replicating the studies — great work. I am also very happy with your responses to my earlier questions.

   Reply: We sincerely thank the reviewer for the very positive evaluation of our revision and for the constructive feedback throughout the review process.

I have only a few minor comments:

1. I suggest removing the word “robust” from the abstract. The sentence “The results were fully replicated...” should be sufficient.

   Reply: Thank you for this suggestion. We have removed the word “robust” from the abstract and now simply state that the results were fully replicated, as suggested.

   Revised Abstract:

   > **…**Results from both experiments were fully replicated in a preregistered replication study (N_1 = 61 and N_2 = 62).

2. I recommend replacing the words “conservative” and “adventurous” with “risk-averse” and “risk-seeking,” respectively, wherever possible.

   Reply: We appreciate this recommendation and agree that “risk-averse” and “risk-seeking” provide clearer and more standard terminology when referring specifically to risk preference. Accordingly, we have replaced “conservative” and “adventurous” with “risk-averse” and “risk-seeking” int the manuscript when referring to risk preference.

   However, in contexts where “conservative” and “adventurous” are used more broadly (e.g., to describe ambiguity-related preferences or general behavioral tendencies not reducible to risk preference alone), we have retained the original wording to avoid confusion.

3. Page 6, line 4: Could you please clarify what is meant by “inverse temperature”?

   Reply: We have added a sentence in the main text explaining that the inverse temperature parameter reflects choice stochasticity (i.e., the sensitivity of choices to subjective value differences), with higher inverse temperature indicating more deterministic choice behavior.

   Revised Results:

   > …and the estimated risk preference was not correlated with the inverse temperature—which captures choice stochasticity, with higher values indicating more deterministic, value-sensitive choices—under the design of the current study…

4. Figures 2f and 2i currently lack a legend. I assume the darker line represents risk-averse participants and the lighter line represents risk-seeking participants—please confirm or clarify.

   Reply: The reviewer's assumption is correct: the darker line corresponds to the risk-averse observee condition, and the lighter line corresponds to the risk-seeking observee condition. We are sorry about the oversight and have now added explicit clarification to the figure captions for Figures 2f and 2i, as well as parallel clarifications to the captions of Figures 4f and 4i.

   Revised Caption of Figure 2:

   > …In (**e**), (**f**), (**h**), and (**i**), each magenta/pink point represents a participant when observing risk-averse/risk-seeking observee, respectively.

   Revised Caption of Figure 4:

   > …In (**e**), (**f**), (**h**), and (**i**), each dark-blue/light-blue point represents a participant when observing the ambiguity-averse/ambiguity-seeking observee, respectively.

5. I appreciated your response to my question on reaction time (R1-4). If possible, I would suggest adding a few lines in the Discussion about how the observed patterns of reaction time could help explain the main results. As a reader I am puzzled by how the reaction time data correlates with asymmetry in risk contagion.

   Reply: Thank you for this comment. Per the reviewer's suggestion, we have added a brief discussion of the reaction time results at the end of the second paragraph of the Discussion, linking the differential RT–distance relationships following risk-averse versus risk-seeking observations to asymmetric cognitive processing underlying risk contagion.

   Revised Discussion:

   > …Consistent with this account, reaction times decreased with increasing observer–observee distance after observing risk-seeking—but not risk-averse—choices, suggesting faster disengagement from risk-seeking social information but continued deliberative integration of risk-averse information.

Reviewer #2 (Remarks to the Author):

The authors conducted a preregistered replication study, performed additional model checks and loss-aversion analysis, and expanded the Discussion to connect their findings to the broader literature on social influence and decision-making. Overall, the authors have fully addressed all of my previous concerns, and the revision has significantly strengthened the paper both methodologically and conceptually. I therefore support acceptance of the manuscript in its current form.

Reply: We thank the reviewer for the positive assessment.

Reviewer #3 (Remarks to the Author):

1. I thank the authors for carefully taking all my comments into account and in particular for taking the effort to collect new data with improved versions of their experiments. I think the additional (replication) experiments greatly improved the empirical evidence for many of the authors' claims. These additional experiments satisfactorily addressed my original comments 1,2, and 4. The only things that were missing for me in the report of these experiments were (sorry if they were there and I just did not find them): First, a detailed explanation of how the human choice data were recorded and selected, which were used to introduce social contagion in the replication studies. And second, an exact description of how the trial-type judgment task was implemented and how participants had to answered it (how was the question framed? Was it multiple-choice? If yes, what were the options?).

   Reply: We have now revised the Methods section of the replication study to clarify both points raised by the reviewer. First, we explain how the human choice data used to generate social information were selected. In the replication study, the observed choices were drawn from pre-recorded decisions of participants in our earlier experiments (i.e., Study 1). Because participants in Blocks 1 and 3 completed the same task as the simulated observees, their choices could be reused as observational input. We thus selected data from participants whose behavior closely matched the intended observee as we designed in the original study.

   Revised Methods 1:

   > …Specifically, the observed choices in the replication study were drawn from pre-recorded choices of participants in our earlier experiments, selected from Sessions 1 and 3 in which participants completed the task individually (the "Self" trials) and therefore could be used as observees' choices in the replication study. We selected data from participants whose behavior closely matched the intended observee's preferences we designed earlier (in the original study).

   Second, we now provide a clearer description of the trial-type judgment task. This task was presented at the end of each mini-block as an attention check.

   Revised Methods 2:

   > …In particular, participants needed to answer a multiple-choice question asking them to indicate which type of block they had just completed—*Self*, *Observe*, or *Predict*—by selecting one of these three options.

2. Concerning Comment 3, I greatly appreciate the extensive parameter recovery, which looks indeed very convincing. I was a bit surprised about this accuracy given the papers I cited in my previous review, which found much worse recoveries. But given that the recovery is so good, the inference is fully warranted. I still think that the model-free analysis should be more prominently featured in the main manuscript as a robustness, but I leave this to the authors. In either case, the current report of the model-free analysis is not fully clear to me. In particular, from the supplementary Figure S4, I did not fully understand which exact analysis was used to show that the asymmetric (Exp 1) or symmetric (Exp 2) contagion effects in the model-free analyses are similar to those reported for the model-based results.

Reply: We have revised the manuscript and Supplementary Information to clarify the model-free analyses and their relation to the model-based results. We now explicitly describe the analytical steps used in the model-free approach and explain how these results parallel those of the model-based analysis.

Specifically, both approaches rely on the same logic: we examine changes in social preference before and after social exposure and relate these changes to the distance between the participant's initial preference and the observees' preference. The key distinction is that the model-free analysis relies directly on observed choice probabilities, whereas the model-based analysis relies on preference measures estimated from the model. For example, in the risk task, the model-free analysis uses the probability of choosing the gamble option, while the model-based analysis uses the estimated risk preference parameter α. Apart from this difference in the dependent variable (DV), all other procedures are identical across the two approaches. This distinction is now clearly stated in the revised manuscript. We also included a detailed explanation of the model-free approach of calculating the risk and ambiguity preferences in the Methods section.

Revised Methods 1:

> In a model-free approach, participants' risk preferences are defined as the proportion of choosing a gamble option relative to the (hypothetical) proportion accepted by a risk-neutral baseline agent: Risk-averse/seeking if less/greater than 0. Apart from this difference in the dependent measures, all other procedures are identical across model-free and model-based approaches.

Revised Methods 2:

> We used a hybrid model-free approach where participants' ambiguity preferences are defined as the proportion of choosing a gamble option relative to the (hypothetical) proportion accepted by an ambiguity-neutral ($\beta_{hypothetical}$ = 0) agent with the same risk preference as the participant ($\alpha_{hypothetical} = \alpha_{self}$). In this approach, positive and negative values indicate ambiguity-seeking and ambiguity-averse preferences, respectively. Apart from this difference in the dependent measures, all other procedures are identical across model-free and model-based approaches.

Crucially, the model-free analyses lead to the same qualitative conclusions as the model-based results. They show asymmetric social influence for risk in Exp. 1 and the lack thereof for ambiguity preference in Exp. 2. More specifically, in **Figure S4e** (corresponding to **Figure 2d**), the model-free risk contagion ratio is larger when participants observe risk-averse behavior than when they observe risk-seeking behavior. The same pattern is also evident in the corresponding correlation analysis shown in **Figure S4f** (corresponding to **Figure 2e**). For ambiguity contagion, the model-free analyses similarly show no reliable evidence for an asymmetric effect: both the contagion-ratio analysis (**Figure S7d**) and the distance-based regression (**Figure S7e**) indicate comparable influence from ambiguity-averse and ambiguity-seeking observees, closely mirroring the model-based results shown in **Figures 4d** and **4e**.

In addition, we have added and highlighted the statistical results of model-free analyses for risk contagion effect (**Figure S4**) and ambiguity contagion effect (**Figure S7**) in the relevant figure captions.

Revised Figure S4 (see Supplementary Information):

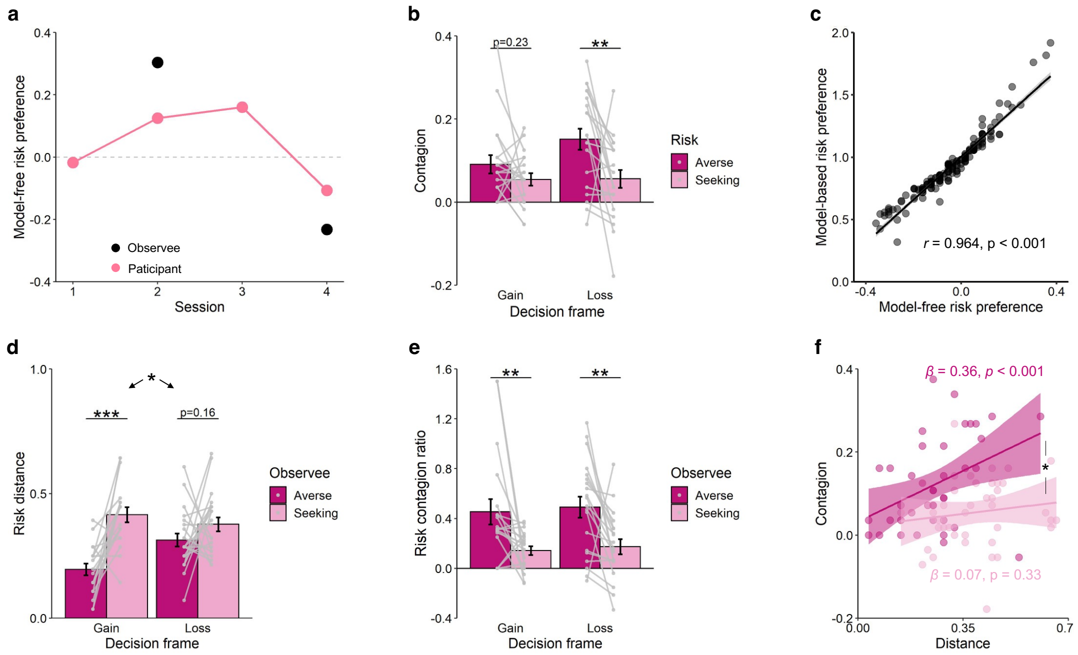

Figure S4. Model-free analysis of risk contagion effect. … In (**e**), an ANOVA analysis revealed a consistent asymmetric risk contagion effect in both the gain and loss frames: subjects were more aligned with risk-averse observees than with risk-seeking ones ($F(1,37) = 17.257$, $p < 0.001$, $\eta_p^2 = 0.32$, 95% CI = [0.13, 1.00]). Neither the main effect of the decision frame ($F(1,37) = 0.218$, $p = 0.644$, $\eta_p^2 < 0.01$, 95% CI = [0.00, 1.00]) nor the frame × preference interaction effect ($F(1,37) = 0.001$, $p = 0.970$, $\eta_p^2 < 0.01$, 95% CI = [0.00, 1.00]) was statistically significant. (**f**) Model-free risk contagion effect as a function of risk distance. Each magenta/pink point represents a participant when observing risk-averse/risk-seeking observees. Fitted regression lines are plotted, and the shaded areas around the fitted lines reflect the 95% CIs. The degree of contagion effect was also significantly correlated with the risk distance when the observee was risk-averse ($coef_{\text{risk-averse}} = 0.363 \pm 0.101$, $p < 0.001$), but not when the observee was risk-seeking ($coef_{\text{risk-averse}} = 0.070 \pm 0.072$, $p = 0.338$). ***$p < 0.001$; **$p < 0.01$; *$p < 0.05$; n.s., not significant.

Revised Figure S7 (see Supplementary Information):

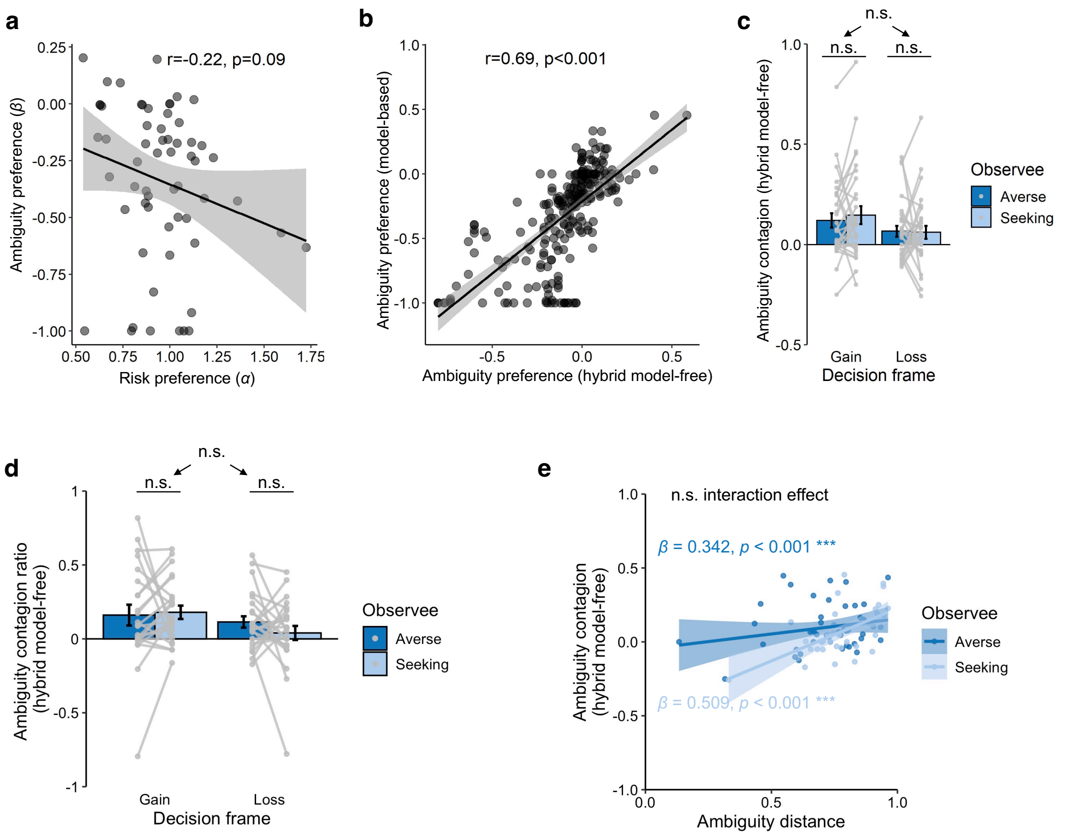

Figure S7. … (**d**) Degree of hybrid-model-free ambiguity contagion ratio as a function of decision frame and observees' ambiguity-preference type. Extreme ratio values falling outside of three standard deviations were excluded. An ANOVA analysis on ambiguity contagion ratio showed that neither the observee's ambiguity preference ($F(1,46) = 0.449$, $p = 0.506$, $\eta_p^2 < 0.01$, 95% CI = [0.00, 1.00]), the decision frame ($F(1,46) = 2.618$, $p = 0.113$, $\eta_p^2 = 0.05$, 95% CI = [0.00, 1.00]), nor the interaction of these two variables affected the ambiguity contagion ratio ($F(1,46) = 1.114$, $p = 0.297$, $\eta_p^2 = 0.02$, 95% CI = [0.00, 1.00]), suggesting no reliable evidence for an asymmetric influence of observees' ambiguity preferences on participants' ambiguity contagion. (**e**) Regression analysis with hybrid model-free ambiguity contagion effect as a function of ambiguity distance. Each blue/light-blue point represents a participant when observing ambiguity-averse/ambiguity-seeking observees. Fitted regression lines are plotted, and the shaded areas around the fitted lines reflect the 95% CIs. Specifically, the ambiguity contagion effect was positively correlated with ambiguity distance no matter when the observee was ambiguity-averse or ambiguity-seeking ($coef_{\text{ambiguity-averse}} = 0.342 \pm 0.095$, $p < 0.001$; $coef_{\text{ambiguity-seeking}} = 0.509 \pm 0.099$, $p < 0.001$; with no significant interaction effect between ambiguity distance and the observee's ambiguity-preference type: $coef_{\text{ambiguity-averse}} - coef_{\text{ambiguity-seeking}} = -0.168 \pm 0.138$, $p = 0.226$), indicating no reliable evidence for an asymmetric effect.

3. Finally, I still struggle with my concerns expressed in my original Comment 5. The authors argue that they have evidence for the absents of asymmetry in social contagion in the domain of ambiguity. However, the Bayes Factor they report in the response letter is 0.55 (and is not reported in the manuscript as far as I can see), which speaks in favor of the null hypothesis, but is indecisive evidence according to standard Bayesian interpretations. Also, the point estimate for an asymmetric effect of social contagion in ambiguity in the pooled data was reported in the response letter as Cohen's d of 0.256. This is small but not negligible and in the range of behavioral effects often found in the field of judgment and decision making. Additionally, although the authors replicated both risk and ambiguity contagion effects independently, they still did not examine both within one experiment. However, the comparison between experiments without a random allocation of participants to the domains is weak causal evidence for a difference in social contagion between both domains. In sum, in my view the authors should be more careful with their conclusions and at least discuss the possibility that the asymmetric contagion effects are just smaller instead of absent in ambiguity.

    Reply: We appreciate the reviewer's concern regarding the interpretation of ambiguity contagion. We have revised the manuscript to clarify that the primary aim of the study is to demonstrate a difference in the degree of asymmetric social influence between risk and ambiguity, rather than to claim a definitive absence of asymmetry in ambiguity preference. In the original, replication studies and pooled subjects, the Bayes Factors for the post-hoc t-test of the asymmetric ambiguity contagion effect were indeed weak but not negligible (BF_{10} = 0.55, BF_{10} = 0.31 and BF_{10} = 0.86, respectively, see Results section in the main manuscript). Accordingly, we have taken the reviewer's suggestion and weakened statements describing ambiguity contagion as "symmetric" and now state that the data provide no reliable evidence for asymmetry in ambiguity contagion.

    We further expand the Discussion to explicitly acknowledge the alternative possibility that asymmetry in ambiguity contagion may be smaller rather than absent, and we note that the Bayes factor provides inconclusive evidence, warranting cautious interpretation. Taken together, the results support a robust asymmetric contagion effect in risk preference and a comparatively weaker, non-conclusive asymmetry in ambiguity preference, highlighting differential social contagion effects across uncertainty types as the central contribution of this study.

    Revised Discussion:

    > **Limitations.** First, although we observed robust asymmetry in risk contagion, evidence regarding asymmetry in ambiguity contagion was inconclusive: Bayesian analyses provided indecisive support for the null hypothesis, and the observed effect size suggests that any asymmetry in ambiguity contagion, if it exists, may be small. Accordingly, the present findings highlight the difference of social influence across different types of uncertainty, with risk contagion showing stronger asymmetry than ambiguity contagion. Future work should further clarify the magnitude and boundary conditions of ambiguity contagion.

    Consistent with this reframing, we revised the title to "***Distinct patterns of social contagion under risk and ambiguity***"
